# Single-cell mapping of chromosome breaks identifies multiple fragile site classes with distinct DNA replication timing landscapes

**DOI:** 10.1038/s41467-026-76451-1

**Published:** 2026-09-23

**Authors:** Jothivanan Elumalai, Ichiro Hiratani

**Affiliations:** 1 Laboratory for Developmental Epigenetics, RIKEN Center for Biosystems Dynamics Research (BDR), Kobe, Japan; 2 Division of Developmental Biology and Regenerative Medicine, Department of Physiology and Cell Biology, Graduate School of Medicine, Kobe University, Kobe, Japan

**Keywords:** Fragile sites, DNA replication, Genomic instability

## Abstract

Common fragile sites (CFSs) are genomic regions susceptible to chromosome breaks. However, because they were identified using cell populations, low-frequency fragile sites (non-CFSs) could have been overlooked. Here we perform single-cell genome-wide mapping and classification of aphidicolin-induced chromosome breaks. In human osteosarcoma cells, 42% of breaks exhibit features of CFSs. The remaining 58% are non-CFSs that are ‘rare’ and replicate throughout the S-phase. Among non-CFSs, we identify early-S replicating breaks associated with transcription-replication conflicts, as well as two additional break classes. One is dependent on transcription and coincides with early-to-late-S replication timing (RT) transition regions, while another class exhibits late-S RT with its frequency increasing upon transcription inhibition. Intriguingly, we find that different RT landscapes are associated with distinct aphidicolin-induced RT behavior and break classes. Our data reveal the frequencies, RT landscapes, and mechanistic differences among distinct break classes, providing a comprehensive view of genomic regions prone to breakage under replication stress.

## Abstract (web summary)

Common Fragile Sites are genomic regions prone to breakage. However, other fragile sites remain underexplored. Here, the authors used single-cell genomics to map replication stress-induced breaks, identifying multiple fragile site classes with distinct features and DNA replication timing landscapes.

## Introduction

Genomic instability is a potential driving force of cancer development and aging^1,2^. Mammalian genomes harbor regions that are inherently susceptible to genomic instability under various cellular stresses. Among these, common fragile sites (CFSs) have been extensively investigated^3,4^. CFSs were originally identified as breaks or gaps in metaphase chromosomes that repeatedly occur when cells are subjected to aphidicolin-induced replication stress^5^. At low doses, aphidicolin, an inhibitor of eukaryotic DNA polymerases^6^, slows down DNA replication fork progression without significantly affecting cell division^5^, a condition often used to mimic replication stress in cancer cells.

Low-dose aphidicolin induces persistent DNA replication in G2/M phase^7^. Recent population-based mitotic DNA synthesis sequencing (MiDAS-seq) studies have revealed that preferential G2/M DNA synthesis sites often coincide with known CFSs^8,9^. In addition, early-replicating fragile sites (ERFSs) were identified through genome-wide mapping of DNA repair protein-bound sites after cytokine-mediated activation of murine B lymphocytes and subsequent treatment with hydroxyurea^10^. However, CFS and ERFS were examined under different experimental conditions, and their relative frequency has remained unexplored.

To date, fragile sites have been identified largely based on population-level analyses, meaning that rare non-CFS sites are probably overlooked. Several years ago, we and others developed a copy number-based single-cell genome-wide DNA replication sequencing (scRepli-seq) technology^11,12^. In addition to studying DNA replication timing (RT) regulation in single S-phase cells, this technology can also perform copy-number variation (CNV) analysis if one uses single G1-phase cells. Recently, using single-cell whole genome sequencing (scWGS), large (>20 Mb) and small (<20 Mb) replication stress-induced CNVs were identified in single mammalian cells^13^. However, only large CNVs were found to display features of CFSs, suggesting there could be other yet-to-be-defined categories of fragile sites.

To perform an unbiased, comprehensive mapping of fragile sites, we employed scWGS from the scRepli-seq framework to identify aphidicolin-induced chromosome breaks in single mammalian cells. By categorizing the breaks based on RT and MiDAS, we identified distinct classes of fragile sites alongside known ones, estimated their relative frequency along with potential mechanisms of breaks, and provided a comprehensive view of the features of different fragile sites prone to genomic instability under mild replication stress. Moreover, we showed that different RT landscapes in untreated cells were associated with distinct aphidicolin-induced RT behavior and chromosome break classes.

## Results

### Copy number-based karyotyping in single mammalian cells

To identify chromosome breaks induced by replication stress, we used human osteosarcoma U2OS cells, as they have been extensively used for population-based CFS mapping, and various genome-wide datasets are publicly available^8,9,14–16^. To determine the cell-cycle duration of U2OS, cells were released from a double thymidine block with or without low-dose (0.4 μM) aphidicolin, collected at 8, 16, and 24 h post-release, and analyzed by DNA staining followed by flow cytometry. From this analysis, the cell cycle duration of untreated U2OS cells was approximately 16 h. In the presence of low-dose aphidicolin, the cell cycle extended to approximately 24 h with the S-phase duration extending from 8 to 16 h (Supplementary Fig. 1A).

**Fig. 1:**
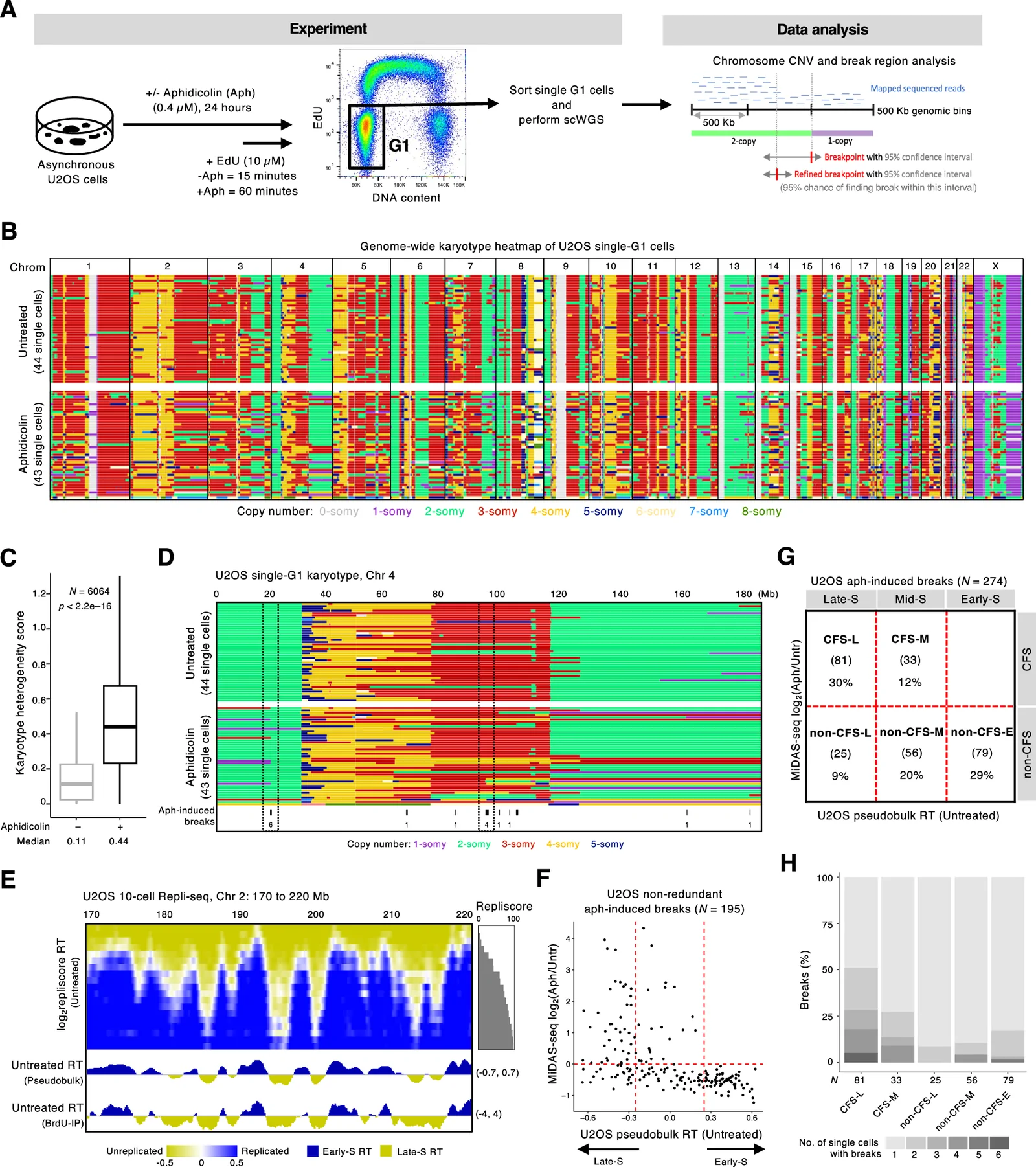
Identification and classification of aphidicolin-induced chromosome breaks. **A** Steps in experiment and data analysis for chromosome copy number and chromosome break analysis. **B** Genome-wide copy number (karyotype) heatmap of single U2OS G1-cells treated with or without low-dose aphidicolin. Copy number calls were performed at 500-kb resolution, and each color represents a unique copy number. **C** Boxplots comparing the karyotype heterogeneity score of each 500-kb genomic bin with or without aphidicolin treatment. Exact *P*-values are shown from two-sided paired Wilcoxon test; values below machine precision are reported as *p* < 2.2e–16. *N*, number of 500-kb genomic bins. **D** Representative human chromosome showing aphidicolin-induced chromosome break regions of U2OS (black bars at the bottom) along with single-cell copy-number heatmap. Numbers below the black bars represent the number of aphidicolin-treated single cells with breaks. Break regions within dotted black lines represent recurrent breaks. **E** Comparison of untreated 10-cell log_2_repliscore RT heatmap, pseudobulk RT, and conventional early/late BrdU-IP RT (average of two replicates) of U2OS cells for a representative genomic region. log_2_repliscore RT heatmap is ordered by the percentage replication score of each 10-cell fraction (gray bars on right). **F** U2OS non-redundant aphidicolin-induced chromosome break regions categorized using U2OS’s untreated pseudobulk RT and U2OS’s aphidicolin-induced MiDAS-seq signals. Vertical red dotted lines categorize the break regions into late, mid, and early S RT. The horizontal dotted red line categorizes the break regions into CFS and non-CFS. **G** Percentage of U2OS’s aphidicolin-induced break regions in each class as defined in (**F**). Number within the bracket in each class is the total number of aphidicolin-induced breaks that belonged to each non-redundant break regions in (**F**). **H** Stacked bar charts showing the percentage of breaks that are recurrent in each class. *N*, number of redundant break regions. For all box plots in this figure, the center line indicates the median, the box limits indicate the 25th and 75th percentiles, and the whiskers extend to the minimum and maximum values excluding outliers (1.5 × interquartile range). Source data are provided as a Source Data file.

After treating U2OS cells with low-dose aphidicolin for a single cell cycle, we used a 2-dimensional (2D) cell-cycle FACS based on DNA content and EdU incorporation for efficient collection of single G1 cells. Because aphidicolin treatment influenced the level of EdU incorporation, 15 and 60 min EdU labeling durations were employed for untreated and aphidicolin-treated conditions, respectively (Supplementary Fig. 1B). Based on the 2D cell-cycle FACS, single G1 cells were sorted, and scWGS from the scRepli-seq framework was performed^17^ (Fig. 1A, Experiment, G1 gate is shown). Briefly, single cells were lysed, genomic DNA was isolated, and whole genome amplification was performed, followed by library preparation for sequencing. After mapping sequenced reads to the hg19 reference genome, chromosome CNV analysis was performed at 500-kb resolution using the AneuFinder program^18^ (Fig. 1A, Data analysis; see Supplementary Note 1 and Supplementary Fig. 1C, D, see also “Methods”).

Untreated U2OS cells displayed an abnormal karyotype with the majority of the chromosomes having three copies (Fig. 1B, top panel; and Supplementary Fig. 1D), consistent with U2OS being hyper-triploid. Upon aphidicolin treatment, karyotype heterogeneity increased as judged by visual inspection (Fig. 1B, compare top and bottom panels), which was statistically confirmed by analyzing the degree of cell-to-cell karyotype heterogeneity (Fig. 1C and see “Methods”).

### Identification of chromosome breaks induced by low-dose aphidicolin

In each single cell treated with or without aphidicolin, a junction between adjacent 500-kb bins with different copy number calls can be regarded as a chromosome breakpoint. As breakpoints need not necessarily be precisely at 500-kb junctions, we refined their positions using uniquely mapped reads and calculated a 95% confidence interval (CI) for each refined breakpoint. We defined this genomic interval (95% CI) as a chromosome break region (or break region for short) (Fig. 1A, Data analysis, and see also “Methods”). Because U2OS cells are karyotypically abnormal, they possess many breaks shared between aphidicolin-treated and untreated conditions (defined as inherent breaks). To eliminate these inherent breaks and focus on the actual breakpoints induced by aphidicolin, we defined aphidicolin-induced break regions as those that neither overlapped with nor were located within ±1 Mb of untreated break regions (see “Methods”). With this approach, we successfully identified 274 aphidicolin-induced break regions from 43 single U2OS cells (Supplementary Data 1). A snapshot of a representative chromosome with aphidicolin-induced break regions is shown in Fig. 1D. Aphidicolin-induced break regions had a median size of 0.27 Mb and 76% of the break regions were less than 0.5 Mb in size (Supplementary Fig. 1E).

To check if and to what extent our break regions overlap with CFSs, we compared our 274 break regions with a database of 324 CFSs identified as sites of G2/M DNA synthesis by aphidicolin-induced MiDAS-seq^9^. We found that 38% (103/274) of all break regions corresponded to CFSs (Supplementary Fig. 1F), which was statistically significant (*p* = 0.001; vs. 1000 randomized control regions). Conversely, 16% (53/324) of MiDAS-seq-based CFSs corresponded to our break regions (Supplementary Fig. 1F and see also Supplementary Note 2). Since some breaks were “recurrent” (i.e., observed within ±100 kb in more than one cell) (Fig. 1D, within dotted lines), we could identify 195 unique break regions out of the 274 aphidicolin-induced break regions. Recurrent break regions that overlapped with CFSs exhibited higher aphidicolin-induced MiDAS-seq signals (Supplementary Fig. 1G). As the 324 CFSs were identified manually based on the peak height^9^, we reanalyzed the MiDAS-seq data (Supplementary Data 2 and 3) and defined break regions with aphidicolin-induced MiDAS-seq signals [i.e., log_2_(aphidicolin/untreated) > 0] as “CFS break regions.” Even for this comparison, 39% (107/274) of our chromosome break regions corresponded to CFS break regions.

While sites of G2/M DNA synthesis identified by MiDAS-seq have been shown to correspond to CFSs^8,9^, historically, CFSs have been defined as cytogenetically identified gaps or breaks observed in mitotic chromosomes^5^. Thus, we utilized the list of 86 cytogenetically-identified aphidicolin-induced CFSs in human fibroblasts^19^ and tested whether our 274 break regions overlap with these sites. We found that 45% (122/274) of our break regions overlapped with the cytogenetically identified CFSs (Supplementary Fig. 1F and see also Supplementary Note 2), which was statistically significant (*p* = 0.001; vs. 1000 randomized control regions). Conversely, 54% (46/86) of cytogenetically identified CFSs corresponded to our break regions (Supplementary Fig. 1F). Taken together, these results indicate that our experimental and analytical setup safely captures known bona fide CFSs.

### Identification of five classes of chromosome breaks based on CFS and RT

A classic feature of known CFSs is their mid-late-S RT^4,20^. Thus, we set out to explore the relationship between RT and chromosome break regions. To this end, 10 cells each from multiple fractions throughout the S-phase (14 fractions in total) were sorted, subjected to scRepli-seq protocol, and the 10-cell Repli-seq log_2_repliscore RT values were calculated genome-wide for each fraction (Supplementary Note 3 and Supplementary Fig. 2A–C). The 10-cell Repli-seq log_2_repliscore RT values of all 14 S-phase fractions were averaged to obtain a “pseudobulk” genome-wide RT profile (Fig. 1E and Supplementary Data 2), which showed a high correlation with log2(early/late) RT profile generated by conventional population Repli-seq based on BrdU-IP [immunoprecipitation of bromodeoxyuridine (BrdU)-substituted DNA] (Fig. 1E; Pearson’s *R* = 0.91, and Supplementary Data 2).

Unexpectedly, we noticed that aphidicolin-induced RT-delayed regions determined by pseudobulk RT comparison frequently corresponded to RT-advanced regions based on BrdU-IP RT comparison (Supplementary Fig. 3A, within dotted lines). Such discrepancy was frequently observed at the 324 CFSs identified as sites of G2/M DNA synthesis by aphidicolin-induced MiDAS-seq, especially late-S replicating CFSs (Supplementary Fig. 3B). Aphidicolin-induced RT advance at CFSs was also reported recently using the conventional two-fraction BrdU-IP Repli-seq^21^. This was puzzling, as CFSs are known to remain under-replicated at the end of S-phase and undergo continued DNA synthesis in G2/M. Consistent with this, CFSs had fewer BrdU-IP reads in late S-phase of aphidicolin-treated cells compared to untreated cells (Supplementary Fig. 3C). This could bias the early-versus-late BrdU-IP ratio and lead to an artificial RT advance rather than a true biological advance.

To test this possibility, we performed BrdU-IP of the very-late-S/G2/M fraction of U2OS cells treated with or without low-dose aphidicolin (Supplementary Fig. 3D, Supplementary Data 2 and see also Supplementary Note 4). Consistent with the pseudobulk RT profile, CFSs frequently showed increased BrdU incorporation in the very-late-S/G2/M fraction upon aphidicolin treatment (Supplementary Fig. 3C, E), indicating that CFSs actually delay their RT, remain under-replicated at the end of S-phase, and continue to replicate during G2/M. This finding highlights the technical limitations of the early versus late S BrdU-IP RT profiling method, as well as the superior temporal resolution of the 10-cell Repli-seq pseudobulk RT profiling approach.

Using the 10-cell Repli-seq pseudobulk RT profile, we explored the RT regulation of CFS and non-CFS break regions. CFS break regions [i.e., aphidicolin-induced MiDAS-seq signals (log_2_(aphidicolin/untreated)) greater than 0] were replicated in mid to late S-phase in untreated U2OS cells, which was expected. By contrast, non-CFS break regions [58% (160/274) of all break regions] showed a wide range of RT throughout the S-phase (Fig. 1F). Therefore, we categorized the break regions based on a combination of untreated pseudobulk RT [early (RT > 0.25), mid (−0.25 < = RT < = 0.25), and late-S (RT < − 0.25)] and “CFS vs non-CFS” axes. We refer to this two-dimensional framework as the “RT-MiDAS axes.” This resulted in the identification of five distinct classes of chromosomal break regions (Fig. 1G): late-S replicating CFS (CFS-L, 30% = 81/274); Mid-S replicating CFS (CFS-M, 12% = 33/274); late-S replicating non-CFS (non-CFS-L, 9% =  25/274); mid-S replicating non-CFS (non-CFS-M, 20% = 56/274); and early-S replicating non-CFS (non-CFS-E, 29% =  79/274). Although six classes are in principle possible with this method of categorization, we did not observe early-replicating CFS-type (CFS-E) breaks. Similar to aphidicolin-induced breaks, categorization of U2OS inherent breaks on the RT-MiDAS axes identified five break classes, suggesting that the exclusion of inherent breaks did not introduce any potential bias (Supplementary Fig. 3F–H and see also “Methods”).

Among the five aphidicolin-induced break classes, CFS-L breaks were more frequently observed across multiple single cells than CFS-M and non-CFS break classes (Fig. 1H and Supplementary Data 4). Because non-CFS breaks were rare, the absence of a break region in untreated cells does not necessarily demonstrate that the region is uniquely induced by aphidicolin, as low-frequency break events may remain undetected in untreated control cells due to finite sampling. We therefore quantified break frequencies at all break regions detected in aphidicolin-treated cells and found that, for all five break classes including non-CFS classes, break frequencies increased following aphidicolin treatment (Supplementary Fig. 4A–D; details in Supplementary Note 5). In addition, we compared the proportions of our five break classes with those obtained from 1000 sets of randomized controls that were first matched for break number and size and subsequently classified according to their positions on the RT-MiDAS axes (see “Methods”). The distributions of our break classes were significantly different from those of the randomized controls, with recurrent CFS-L breaks markedly overrepresented (Supplementary Fig. 3H and Supplementary Data 4).

Thus, the systematic and quantitative classification of aphidicolin-induced breaks reveals the presence of genomic regions that are intrinsically prone to replication stress-induced breakage, which were nonrandomly distributed across the genome. In the sections that follow, we characterize each of these five break classes in detail.

### CFS-L and CFS-M breaks show classic CFS features

**Fig. 2:**
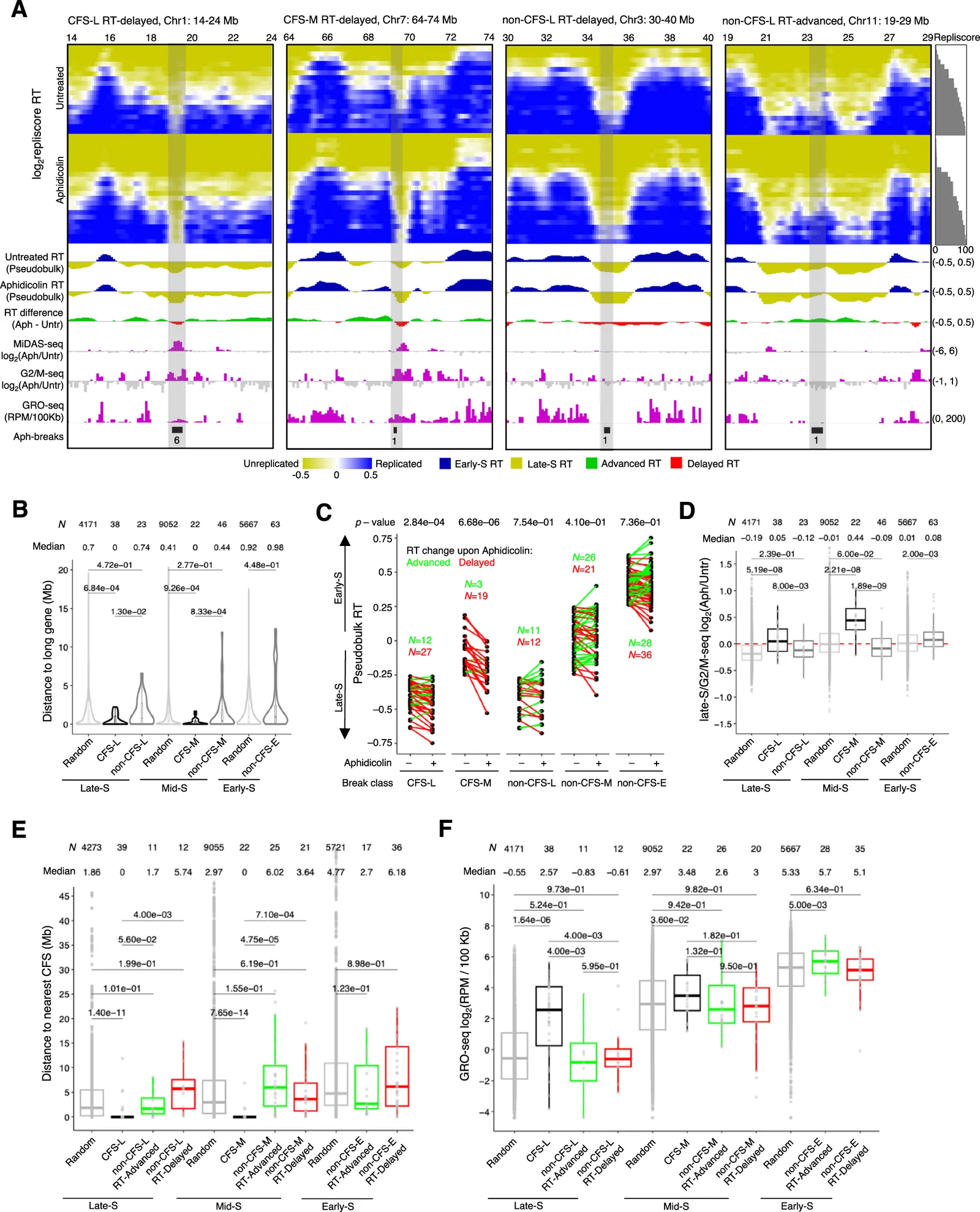
CFS-L and CFS-M breaks are classic CFSs. **A** Comparison of log_2_repliscore RT and pseudobulk RT with or without aphidicolin treatment, aphidicolin-induced RT changes, MiDAS-seq, late-S/G2/M-seq, and untreated GRO-seq (RPM/100 kb) signals at a representative genomic region showing CFS-L, CFS-M, and non-CFS-L RT-delayed and advanced break regions. Break regions are within the gray highlighted area. **B** Violin plots showing the distance to nearest long genes (> = 300 kb) from break regions in each class (CFS, black; non-CFS, dark gray) and randomized control regions with similar RT (light gray). Exact *P*-values are shown from two-sided unpaired Wilcoxon test. *N*, number of non-redundant breaks or randomized control regions. **C** Paired dot plot comparing pseudobulk RT with or without aphidicolin treatment in each class of break. Lines represent the direction and degree of RT change (delayed, red; advanced, green) upon aphidicolin treatment. Exact *P*-values are shown from two-sided paired Wilcoxon test. *N*, number of aphidicolin-induced non-redundant breaks. **D** Boxplots with jitters showing log_2_(aphidicolin/untreated) late-S/G2/M-seq values for breaks in each class (CFS, black; non-CFS, dark gray) and similar RT randomized control regions (light gray). Exact *P*-values are shown from two-sided unpaired Wilcoxon test. *N*, number of non-redundant breaks or randomized control regions. **E** Boxplots with jitters showing distance to nearest MiDAS-seq’s CFSs from break regions in each class (CFS, black; non-CFS advanced, green; non-CFS delayed, red) and similar RT randomized control regions (light gray). Exact *P*-values are shown from two-sided unpaired Wilcoxon test. *N*, number of non-redundant breaks or randomized control regions. **F** Boxplots with jitters showing untreated U2OS’s GRO-seq signals (log_2_(RPM/100 kb)) for break regions in each class (CFS, black; non-CFS advanced, green; and non-CFS delayed, red) and similar RT randomized control regions (light gray). Exact *P*-values are shown from two-sided unpaired t-test. *N*, number of non-redundant breaks or randomized control regions. For all box plots in this figure, the center line indicates the median, the box limits indicate the 25th and 75th percentiles, and the whiskers extend to the minimum and maximum values excluding outliers (1.5 × interquartile range). Source data are provided as a Source Data file.

CFS-L and CFS-M break regions, which were classified as CFSs (i.e., MiDAS-seq positive regions), were replicated in late and mid-S-phase, respectively, by 10-cell Repli-seq’s pseudobulk RT (Figs. 1F, G, and 2A). By CNV analysis, they were frequently associated with large copy number gains and losses (Supplementary Fig. 5A, see median sizes), while they did not show bias toward gains or losses (Supplementary Fig. 5E, Supplementary Data 4, and see also “Methods”). They were located closer to long genes (> = 300 kb) than randomized control with similar RT (Fig. 2B, see Supplementary Fig. 6A, and also “Methods”), consistent with previous reports^13,19,20,22^. Upon aphidicolin treatment, they frequently exhibited delayed RT (Fig. 2A, C, and see Supplementary Note 6) and under-replication at the end of S-phase (Fig. 2A, gray highlighted regions), consistent with their enrichment for aphidicolin-induced G2/M replication signals compared to randomized control with similar RT (Fig. 2A, D, Supplementary Fig. 6B, and see also Supplementary Note 4). Moreover, the degree of RT delay correlated well with aphidicolin-induced MiDAS-seq signal intensity (Supplementary Fig. 6C), suggesting that aphidicolin-induced RT changes identified using pseudobulk RT can be used to locate CFSs.

The relationship between CFSs and topologically associating domain (TAD) boundaries has been debated^9,20^. To address this issue, we reanalyzed publicly available U2OS Hi-C data^23^ and identified TAD boundaries using insulation scores (see “Methods”). Consistent with an earlier study^9^, we found that CFS-L and CFS-M breaks were not significantly closer to TAD boundaries compared to randomized controls with similar RT (Supplementary Fig. 6D–F and see Supplementary Note 7). Taken together, our data suggest that CFS-L and CFS-M break regions are classic CFSs, which are not necessarily close to TAD boundaries. Furthermore, aphidicolin-induced incomplete DNA replication could be the potential cause of the breaks.

### Non-CFS-L RT-delayed breaks represent a category distinct from CFS-L

The non-CFS-L break regions were as late replicating as CFS-L regions in untreated cells (Figs. 1F and 2A, C). However, they were much less recurrent than CFS-L breaks (Fig. 1H and Supplementary Data 4) and exhibited both RT delay and advance upon aphidicolin treatment (Fig. 2A, C). In addition, they were not close to long genes (Fig. 2B), TAD boundaries (Supplementary Fig. 6D), or CFSs (Fig. 2E), nor were they associated with large CNVs (Supplementary Fig. 5A).

As CFSs often coincide with actively transcribed genes, we asked if the “rare” non-CFS-L breaks were transcriptionally less active. To this end, we reanalyzed publicly available untreated U2OS global run-on (GRO)-seq data^24^ (Supplementary Data 2) and confirmed that CFS-L and CFS-M break regions were more transcriptionally active compared to randomized control regions with similar RT (Fig. 2A, F). By contrast, non-CFS-L break regions showed lower transcriptional activity compared to CFS-L break regions whether they advanced or delayed RT upon aphidicolin treatment, but were not significantly different from randomized control with similar RT (Fig. 2A, F). Together, our data suggests that non-CFS-L represents a break type distinct from CFS-L.

**Fig. 3:**
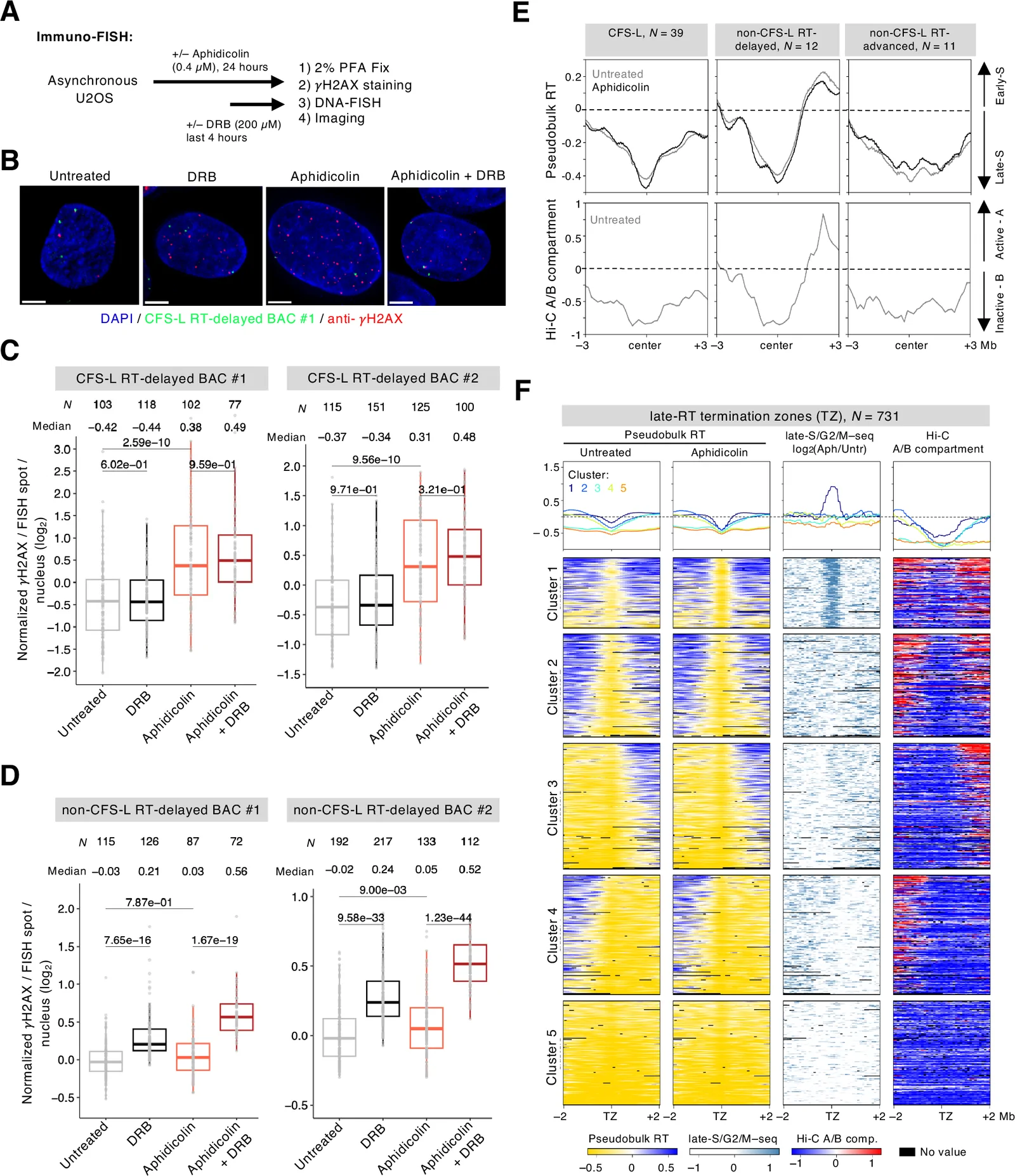
Non-CFS-L RT-delayed breaks represent a category distinct from CFS-L. **A** Schematic of immuno-FISH experiment. **B** Representative immuno-FISH images of U2OS cells treated with or without low-dose aphidicolin and/or DRB. Representative CFS-L break regions are shown by DNA-FISH (green), γH2AX (red), and nuclei (blue). Scale bar is 5 μM. Boxplots with jitters showing logarithmic average γH2AX signals at CFS-L RT-delayed (**C**) and non-CFS-L RT-delayed (**D**) DNA-FISH foci per nuclei in different conditions (untreated, grey; DRB, black; aphidicolin, red; and aphidicolin+DRB, crimson). Two representative break regions were tested with two biological replicates. Exact *P*-values are shown from two-sided unpaired t-test. *N*, number of nuclei. **E** Average line plot showing pseudobulk RT (top) and untreated Hi-C A/B compartments (bottom) at and around CFS-L and non-CFS-L break regions. Non-CFS-L breaks were sub-categorized by aphidicolin-induced RT changes (i.e., advanced or delayed). *N*, number of non-redundant break regions. **F** Average line plot and heatmap showing pseudobulk RT with or without aphidicolin treatment, aphidicolin-induced G2/M replication, and untreated A/B compartment scores at and surrounding the late-RT TZs (±2 Mb from the center of the TZs). K-means clustering (*K* = 5) and ordering were done using untreated RT of late-RT TZs. *N*, number of late-RT TZs. For all box plots in this figure, the center line indicates the median, the box limits indicate the 25th and 75th percentiles, and the whiskers extend to the minimum and maximum values excluding outliers (1.5 × interquartile range). Source data are provided as a Source Data file.

To further validate this distinction, we next tested how genomic regions corresponding to CFS-L and non-CFS-L RT-delayed breaks (matched for RT and aphidicolin-induced RT-change) respond to transcription inhibition. Specifically, we performed immuno-FISH to investigate the dependency of aphidicolin-induced DNA damage (as indicated by γH2AX; used as a proxy for break regions) on transcription (Fig. 3A and see “Methods”). We used 5,6-dichloro-1-β-D-ribofuranosylbenzimidazole (DRB, 200 μM) for 4 h as it effectively inhibited transcription in U2OS cells compared to α-amanitin (Supplementary Fig. 6G and see “Methods”). Furthermore, 200 μM DRB treatment for 4 h did not drastically affect the cell-cycle distribution in both untreated and aphidicolin-treated conditions (Supplementary Fig. 6H). By quantifying γH2AX signals in each segmented nuclei, we found that γH2AX signals increased upon DRB and aphidicolin treatment, with the latter leading to higher γH2AX signals (Fig. 3B, Supplementary Fig. 6I, and see also Discussion). Interestingly, aphidicolin-induced γH2AX signals were decreased upon DRB treatment (Fig. 3B and Supplementary Fig. 6I).

In addition to whole nuclei-level quantification, we measured γH2AX signals at two representative break regions for CFS-L and non-CFS-L using DNA-FISH probes (Fig. 3B, green foci). As expected, we found that γH2AX intensity at CFS-L RT-delayed break regions increased significantly upon aphidicolin treatment (Fig. 3C). By contrast, γH2AX signals at non-CFS-L RT-delayed break regions did not change upon aphidicolin treatment (Fig. 3D), possibly because they were rare events. Interestingly, transcription inhibition did not change the γH2AX signal intensity at transcriptionally active CFS-L RT-delayed break regions in both untreated and aphidicolin conditions (Fig. 3C), consistent with a previous report^25^. By contrast, γH2AX signal intensity at non-CFS-L RT-delayed break regions was increased upon transcription inhibition in both untreated and aphidicolin conditions (Fig. 3D). These results are consistent with the idea that CFS-L and non-CFS-L RT-delayed breaks represent two distinct types of late-replicating fragile sites.

As for the non-CFS-L RT-advanced breaks, they also did not show features of classic CFSs (Fig. 2A, E, F). Further analysis is needed to understand the cause of these breaks.

### Steep late-S RT valleys are associated with CFS-L breaks

Interestingly, we noticed that RT landscapes of CFS-L and non-CFS-L break regions were distinct. In both untreated and aphidicolin-treated conditions, CFS-L and non-CFS-L RT-delayed breaks corresponded to steep and moderately steep late-S RT valleys, respectively, compared to non-CFS-L RT-advanced breaks, which corresponded to shallow RT valleys (Figs. 2A and 3E, top row). Since CFS-L and non-CFS-L RT-delayed breaks showed increased under-replication in G2/M compared to non-CFS-L RT-advanced breaks (Supplementary Fig. 6J), we asked if the steepness of late-S RT valley correlates with a higher under-replication tendency.

To test this hypothesis, we first identified the local minimum positions of RT (i.e., termination zones, TZs) in untreated conditions (Supplementary Fig. 6K, see also Methods and Supplementary Note 8), and TZs that replicated in late-S phase (RT < − 0.25) were selected. Then, we performed a K-means clustering of RT surrounding these late-S TZs into five groups, which distinguished TZs with moderately steep RT valleys (cluster-2) and steep RT valleys (cluster-1) from TZs with a shallow late RT slope on one side (cluster-3 and cluster-4) or both sides (cluster-5) (Fig. 3F). In support of our hypothesis, only cluster 1 clearly showed enrichment for aphidicolin-induced G2/M replication, suggesting that steep late-S RT valleys tend to remain under-replicated upon aphidicolin treatment (Fig. 3F).

Interestingly, the steepness of RT valleys reflects smaller distances covered by unidirectional forks within a given duration^26^, i.e., slow fork movement. This suggest that slow fork movement could be yet another feature of CFSs. In addition, consistent with the tight correlation between RT and Hi-C A/B compartments^27,28^, moderately steep (cluster 2) and shallow (cluster 5) late-S RT valleys corresponded to moderately steep and shallow B-compartment valleys (Fig. 3F), respectively, as revealed by the reanalysis of publicly available U2OS Hi-C data^23^ (Supplementary Data 2). Furthermore, non-CFS-L RT-delayed and RT-advanced breaks exhibited moderately steep and shallow B-compartment valleys, respectively (Fig. 3E, bottom row). Taken together, these findings suggest that steep late-S RT valleys in untreated cells are associated with increased aphidicolin-induced under-replication in G2/M and represent a characteristic feature of CFS-L break regions.

### Non-CFS-M RT-delayed breaks are distinct and coincide with early/late-S TTRs

**Fig. 4:**
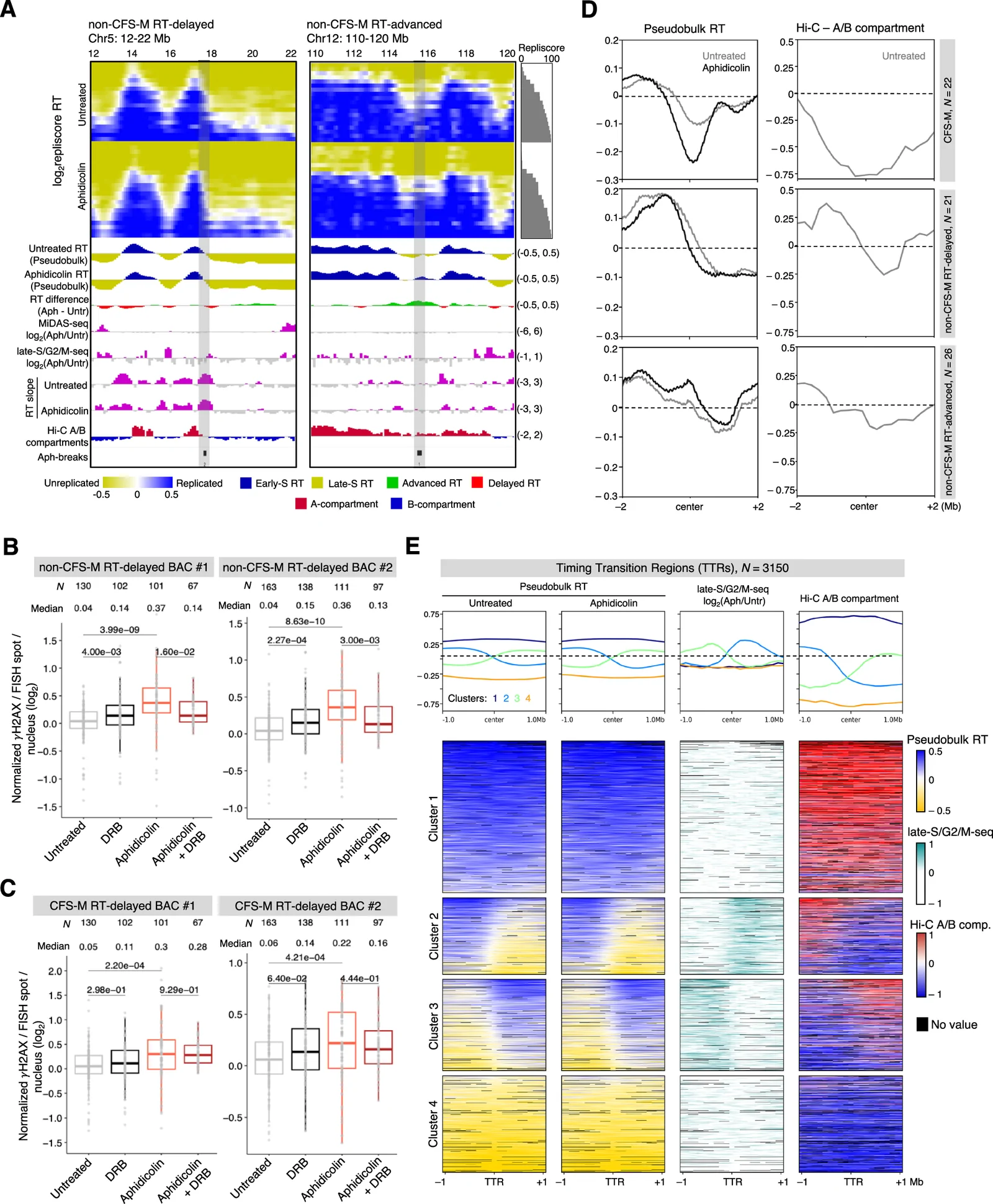
Transcription-dependent non-CFS-M RT-delayed breaks coincide with E/L-TTRs. **A** Comparison of log_2_repliscore RT and pseudobulk RT with or without aphidicolin treatment, aphidicolin-induced RT changes, MiDAS-seq, late-S/G2/M-seq, RT slope values, untreated Hi-C A/B compartment score at representative non-CFS-M RT-delayed and advanced break regions. Break regions are within gray highlighted area. Boxplots with jitters showing logarithmic average γH2AX signals at non-CFS-M RT-delayed (**B**) and CFS-M RT-delayed (**C**) break foci per nuclei in different conditions (untreated, grey; DRB, black; aphidicolin, red; and aphidicolin+DRB, crimson). Two representative break regions were tested with two biological replicates. Exact *P*-values are shown from two-sided unpaired t-test. *N*, number of nuclei. **D** Average line plot showing pseudobulk RT (left) and Hi-C A/B compartment values (right) at and around CFS-M and non-CFS-M break regions. Non-CFS-M breaks were sub-categorized by aphidicolin-induced RT changes (i.e., advanced or delayed). *N*, number of non-redundant break regions. **E** Average lineplot and heatmap showing pseudobulk RT with or without aphidicolin treatment, aphidicolin-induced G2/M replication, and untreated A/B compartment scores at and surrounding the TTRs (±1 Mb from the center of the TTRs). K-means clustering (*K* = 4) and ordering were done using untreated RT of TTRs. *N*, number of TTRs. For all box plots in this figure, the center line indicates the median, the box limits indicate the 25th and 75th percentiles, and the whiskers extend to the minimum and maximum values excluding outliers (1.5 ×  interquartile range). Source data are provided as a Source Data file.

Non-CFS-M break regions are mid-S-replicating in untreated conditions (Figs. 1F, 2C, and 4A) and exhibited both RT delay and advance upon aphidicolin treatment (Figs. 2C and 4A). Similar to non-CFS-L breaks, they were away from long genes (Fig. 2B), CFSs (Fig. 2E), and TAD boundaries (Supplementary Fig. 6D), compared to randomized controls with similar RT. In addition, they were not associated with large CNVs (Supplementary Fig. 5A, see median size difference) and showed no significant difference in transcriptional activity compared to randomized controls with similar RT (Fig. 2F).

Interestingly, upon transcription inhibition, representative non-CFS-M RT-delayed break regions exhibited a decrease in aphidicolin-induced γH2AX signals (Fig. 4B), while no significant difference was observed at CFS-M breaks matched for RT and aphidicolin-induced RT change (Fig. 4C). Furthermore, non-CFS-M RT-delayed break regions were also distinct from CFS-L and non-CFS-L RT-delayed breaks, which showed no change and an increase, respectively, in γH2AX signals upon transcription inhibition (Figs. 3C, D, and 4B). These results suggest that non-CFS-M RT-delayed breaks could form in a manner mechanistically distinct from CFS-L, CFS-M, and non-CFS-L RT-delayed break regions.

In both untreated and aphidicolin-treated conditions, non-CFS-M RT-delayed but not RT-advanced break regions were frequently present at strong RT transition regions (TTRs)^26,29,30^, where forks travel uni-directionally (Fig. 4A, left and Fig. 4D, second row on the left; Supplementary Fig. 7A). To systematically assess the occurrence of breaks at TTRs, we measured the absolute RT slope value, which is high at TTRs (Supplementary Fig. 7B and Supplementary Data 2). Non-CFS-M RT-delayed break regions indeed had a higher RT slope score compared to randomized control regions with similar RT (Supplementary Fig. 7C). Importantly, non-CFS-M RT-delayed breaks were frequently present at TTRs, where replication forks travel long distance from early to late S-phase (Fig. 4A, D). Our results suggest that for non-CFS-M RT-delayed breaks, long TTRs at early/late RT boundary and transcription contribute to their fragility upon replication stress.

### Early/late-S TTRs exhibit aphidicolin-induced under-replication in G2/M

Since TTRs are replication initiation-poor regions^26,30,31^, we asked if long early/late-S TTRs remained under-replicated in G2/M upon low-dose aphidicolin, leading to chromosome breakage. To this end, we first identified TTRs genome-wide by selecting regions with absolute RT slope values greater than the mean in untreated U2OS cells (Supplementary Fig. 7B and see “Methods”). Then, we performed K-means clustering of RT surrounding these TTRs into four groups, which identified clusters that shifted their RT from early to late-S (from left to right; cluster-2) or vice versa (cluster-3), as well as those that mildly shifted RT within the first (cluster-1) and second (cluster-4) half of S-phase (Fig. 4E).

Clusters 2 and 3, but not clusters 1 and 4, clearly showed enrichment for aphidicolin-induced G2/M replication, suggesting that the TTRs coinciding with early/late-S RT boundaries tend to remain under-replicated toward the later RT side upon aphidicolin treatment (Fig. 4E). Consistently, non-CFS-M RT-delayed break regions showed significantly higher under-replication in aphidicolin-treated G2/M phase compared to non-CFS-M RT-advanced break regions, although not to the level similar to CFS-M break regions (Supplementary Fig. 7D). Furthermore, early/late-S TTRs corresponded well with A/B compartment boundaries, consistent with the correlation of early and late S RT domains with A and B compartments, respectively (Fig. 4E). This was also the case for non-CFS-M RT-delayed breaks but not CFS-M break regions (Fig. 4D, right column). Thus, mid-S RT/compartment landscapes, in particular, the early/late-S TTRs, are associated with increased susceptibility to aphidicolin-induced under-replication in G2/M, similar to the case of late-S steep RT valleys (Fig. 3F). These results suggest that non-CFS-M RT-delayed break regions, which are dependent on transcription and frequently present at early/late TTRs as well as A/B compartment boundaries, likely remain under-replicated at G2/M upon aphidicolin treatment, leading to their fragility.

As for the non-CFS-M RT-advanced breaks, we frequently observed aphidicolin-induced initiation-like events at the break regions (Fig. 4A, right and Fig. 4D, third row on the left). However, further analysis is needed to understand the cause of the non-CFS-M RT-advanced break regions.

### Non-CFS-E RT-advanced breaks show features of transcription-replication conflicts

**Fig. 5:**
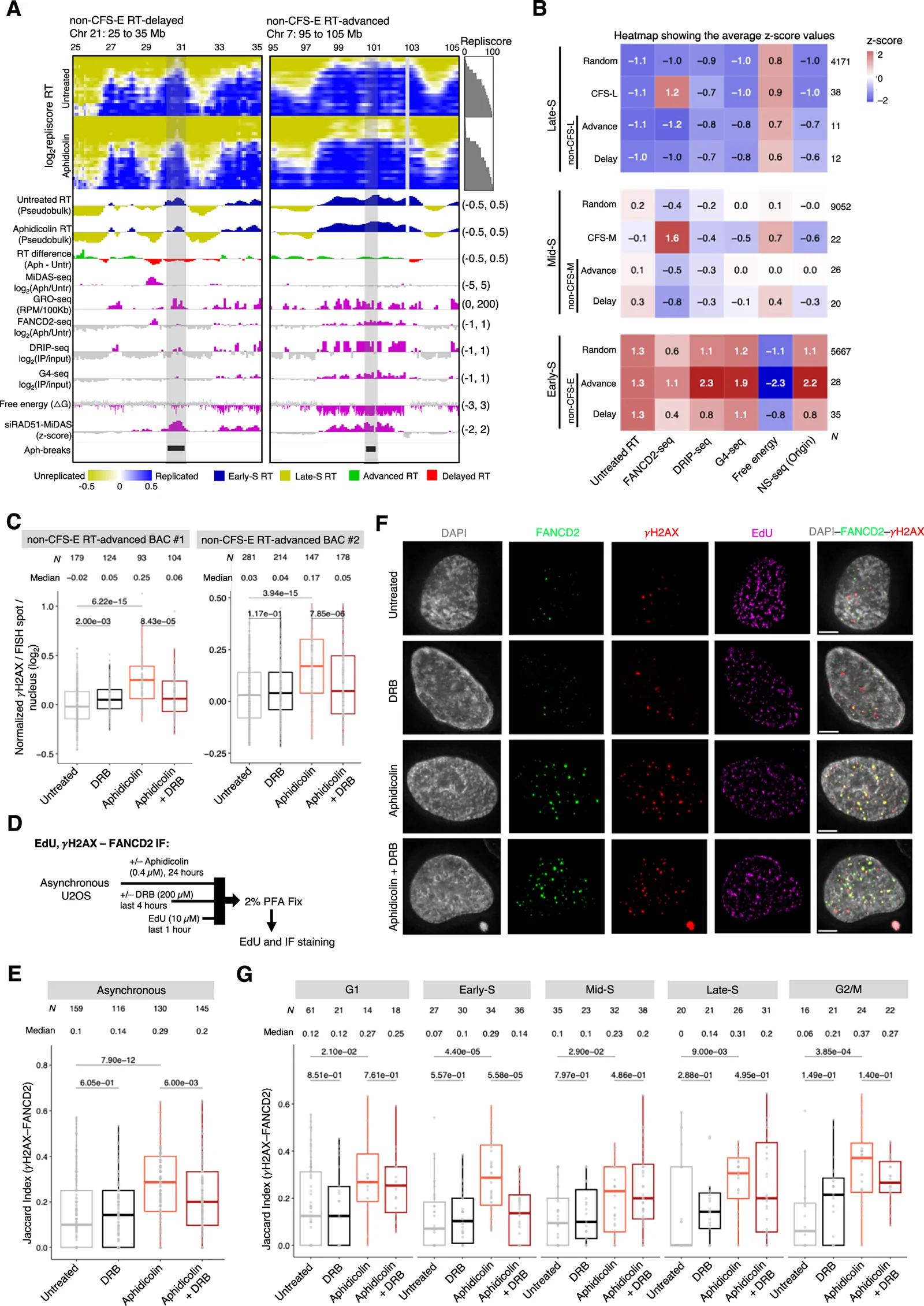
Non-CFS-E RT-advanced breaks are associated with TRCs. **A** Comparison of log_2_repliscore RT and pseudobulk RT with or without aphidicolin treatment, aphidicolin-induced RT changes, MiDAS-seq, FANCD2 ChIP-seq, untreated GRO-seq, DRIP-seq, G4-seq, DNA free energy, and siRAD51 MiDAS-seq signals at a representative genomic region showing non-CFS-E RT-delayed and advanced break regions. Break regions are within the gray highlighted area. **B** A heatmap table showing average z-score values of untreated pseudobulk RT, aphidicolin-induced FANCD2, untreated DRIP-seq, G4-seq, DNA free energy, and NS-seq, for both RT-delayed and advanced break regions of all 5 classes in comparison with RT-matched random control regions. *N*, number of non-redundant breaks or randomized control regions. Statistics are provided in Supplementary Data 4. **C** Boxplots with jitters showing logarithmic average γH2AX signals at non-CFS-E RT-advanced break foci per nuclei in different conditions (untreated, grey; DRB, black; aphidicolin, red; and aphidicolin+DRB, crimson). Two representative non-CFS-E RT-advanced break regions were tested with two biological replicates. Exact *P*-values are shown from two-sided unpaired t-test. *N*, number of nuclei. **D** Design of EdU, γH2AX–FANCD2 immunofluorescence (EdU and IF) experiment to check for their colocalization with or without aphidicolin and/or DRB. **E** Boxplots with jitters showing colocalization of γH2AX-FANCD2 foci per nuclei using Jaccard index in asynchronous population with different conditions (untreated, grey; untreated+DRB, black; aphidicolin, red; and aphidicolin+DRB, crimson). Two biological replicates were performed. Exact *P*-values are shown from two-sided unpaired t-test. *N*, number of nuclei. **F** Representative early-S phase nuclei showing FANCD2 and γH2AX signals with or without aphidicolin and/or DRB. Scale bar is 5 μM. **G** Boxplots with jitters showing colocalization of γH2AX-FANCD2 foci per nuclei using Jaccard index in different cell cycle phases and different treatment (untreated, grey; DRB, black; aphidicolin, red; and aphidicolin+DRB, crimson). Two biological replicates were performed. Exact *P*-values are shown from two-sided unpaired Wilcoxon test. *N*, number of nuclei. For all box plots in this figure, the center line indicates the median, the box limits indicate the 25th and 75th percentiles, and the whiskers extend to the minimum and maximum values excluding outliers (1.5 × interquartile range). Source data are provided as a Source Data file.

Non-CFS-E break regions were early-S replicating in untreated conditions (Figs. 1F and 2C), and like other non-CFSs, they exhibited both RT delay and advance upon aphidicolin treatment (Figs. 2C and 5A). Non-CFS-E breaks were also far from long genes (Fig. 2B), not associated with large CNVs (Supplementary Fig. 5A), and did not show enrichment for aphidicolin-induced G2M-seq signals (Supplementary Fig. 8A), suggesting that under-replication may not be the potential cause of these breaks.

Given the gene-rich nature of early-RT regions, we speculated that transcription-replication conflicts (TRCs) may be involved in non-CFS-E break formation. Therefore, we reanalyzed various publicly available TRC-associated genome-wide datasets, calculated their average z-score values for each of the five break classes, and compared them to randomized controls with similar RT (Fig. 5B and Supplementary Data 4 for statistics). In the untreated condition, both aphidicolin-induced RT advanced and delayed non-CFS-E break regions exhibited similar RT (Fig. 5A, B, and Supplementary Data 4), whereas only non-CFS-E RT-advanced break regions displayed higher transcriptional activity (Fig. 2F), compared to randomized controls with similar RT.

Interestingly, Fanconi Anemia Group D2 (FANCD2) protein, which is known to bind to CFSs^15^, was also enriched at non-CFS-E RT-advanced break regions (Fig. 5A, B, Supplementary Data 2 and 4). FANCD2 has been implicated in resolving TRCs involving DNA:RNA hybrid structures (R-loops)^32^. To test if non-CFS-E RT-advanced break regions are enriched for R-loops, we reanalyzed publicly available DNA:RNA hybrid immunoprecipitation sequencing (DRIP-seq) data from untreated U2OS cells^33^ (Supplementary Data 2) and observed an enrichment of R-loops at non-CFS-E RT-advanced break regions compared to randomized controls with similar RT (Fig. 5A, B, and Supplementary Data 4). Because R-loops have been linked to G-quadruplex (G4) structure-forming regions^34^, we reanalyzed U2OS G4 ChIP-seq data^35^ (Supplementary Data 2) and found a strong positive correlation between G4 structures and R-loops (*R* = 0.71). Consistently, non-CFS-E RT-advanced break regions with increased R-loops were also enriched for G4 structure formation and showed increased DNA secondary structure stability, as indicated by more negative DNA free energy (Δ*G*) values (Fig. 5A, B, and Supplementary Data 4; see also “Methods”). As G4 structure-forming regions are associated with DNA replication initiation sites^36,37^, we asked if non-CFS-E RT-advanced break regions were enriched for initiation sites. Reanalysis of publicly available nascent strand sequencing (NS-seq) data from untreated U2OS cells revealed a significant enrichment of initiation sites for non-CFS-E RT-advanced break regions compared to randomized controls with similar RT (Fig. 5A, B, Supplementary Data 2 and 4).

Furthermore, early replicating fragile sites (ERFS)^10,38^, oncogene-induced origins^39^, and atypical-MiDAS regions^16,40^ were shown to be early-S replicating and have been attributed to TRCs^16^. Therefore, we compared non-CFS-E break regions to the above-mentioned datasets. Both RT advanced and delayed non-CFS-E break regions were not enriched for atypical MiDAS compared to randomized controls with similar RT (Supplementary Fig. 8B and Supplementary Data 2). Non-CFS-E RT-advanced break regions were enriched for DNA replication origins in normal condition^39^ (Supplementary Fig. 8C, left, and Supplementary Data 2), consistent with our comparison to NS-seq data (Fig. 5B). However, both RT advanced and delayed non-CFS-E break regions were not enriched for oncogene-induced short-G1 origins (Supplementary Fig. 8C, right, and Supplementary Data 2). As ERFSs were identified in mice, we converted their chromosomal coordinates to those in humans (see “Methods”). Interestingly, both RT-delayed and advanced non-CFS-E breaks were closer to ERFSs compared to randomized controls with similar RT (Supplementary Fig. 8D). Our correlative analysis suggested that origin-rich and transcriptionally active non-CFS-E RT-advanced breaks were close to ERFSs and could likely arise from TRCs.

As for non-CFS-E RT-delayed breaks, in addition to being closer to ERFSs, they were also more frequently flanked by sharper RT and A/B compartment transition regions than non-CFS-E RT-advanced breaks (Supplementary Fig. 8E). Further studies are needed to understand the cause of non-CFS-E RT-delayed breaks.

### DNA damage at non-CFS-E RT-advanced breaks decreased upon transcription inhibition

We investigated the dependence of non-CFS-E RT-advanced breaks on transcription and found that aphidicolin-induced γH2AX signal at representative non-CFS-E RT-advanced break regions was decreased upon transcription inhibition (Fig. 5C). To expand our findings beyond the two representative FISH probes, we used FANCD2 foci in early S-phase as a proxy for non-CFS-E RT-advanced break regions. Thus, we performed double immunostaining of γH2AX and FANCD2 to determine if transcription inhibition affected their colocalization in early-S phase (Fig. 5D and see “Methods”). Transcription inhibition in asynchronous population did not change the number of γH2AX and FANCD2 foci in aphidicolin-treated cells (Supplementary Fig. 8F, G). However, aphidicolin-induced γH2AX–FANCD2 colocalization was decreased upon transcription inhibition (Fig. 5E).

To test if the decreased γH2AX–FANCD2 colocalization was specific to early-S phase, nuclei were cell-cycle-phased using DAPI and EdU (Supplementary Fig. 8H). In line with our hypothesis, aphidicolin-induced γH2AX–FANCD2 colocalization decreased significantly upon transcription inhibition only in early-S phase (Fig. 5F, G). Furthermore, aphidicolin-induced γH2AX–FANCD2 colocalization did not change significantly upon transcription inhibition in mid- and late-S phases (Fig. 5G), which was consistent with our immuno-FISH results for CFS-L and CFS-M breaks (Figs. 3C and 4C).

Because aphidicolin substantially alters cell-cycle distribution (Supplementary Fig. 6H), we also examined whether γH2AX levels differ systematically across cell-cycle phases. As no substantial difference was observed across cell-cycle phases (Supplementary Fig. 8H–J), our data suggest that altered cell-cycle composition alone is unlikely to fully account for the γH2AX enrichment observed at representative non-CFS loci following aphidicolin treatment (see Supplementary Note 9 for details). Taken together, both immuno-FISH and γH2AX–FANCD2 colocalization results validated the dependence of non-CFS-E RT-advanced breaks on transcription and support our hypothesis that TRCs are a key mechanism underlying their fragility.

### RT landscapes in untreated cells can distinguish different break classes

**Fig. 6:**
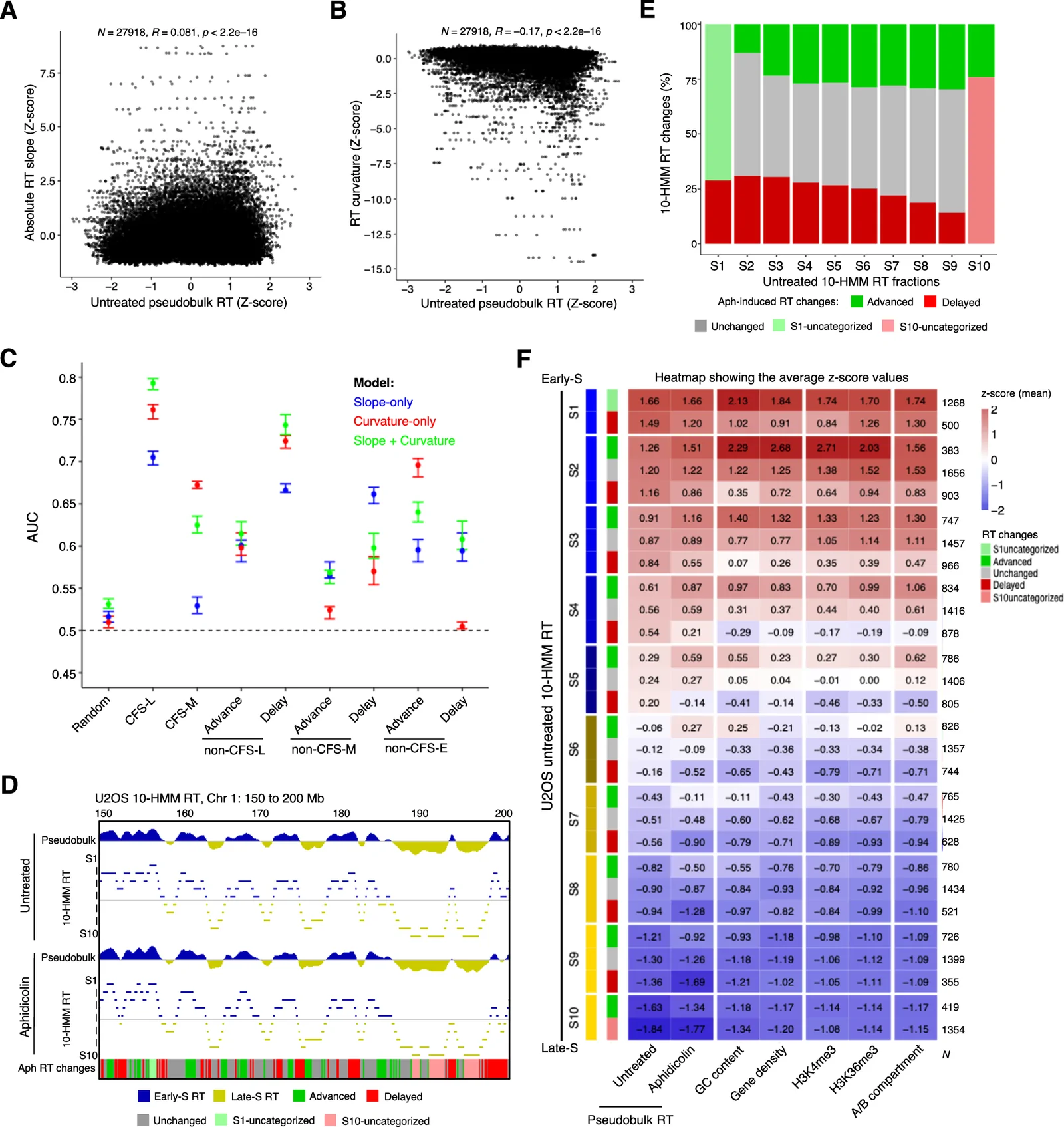
RT landscapes can distinguish aphidicolin-induced RT changes and break classes. Scatter plot showing correlation between RT and RT slope (**A**), and between RT and RT curvature (**B**). *N*, number of 100-kb bins. Pearson correlation coefficient (*R*) and exact *P*-value from a two-sided Pearson correlation test are shown; values below machine precision are reported as *p* < 2.2e-16. **C** Area under the curve (AUC) score with 95% confidence intervals evaluating the performance of generalized linear models (glm) based on untreated RT landscape features (slope – blue, curvature – red, and slope + curvature – green) for predicting break classes. Points represent the observed AUC value, and error bars indicate the 95% confidence interval estimated by stratified bootstrap resampling (*N* = 1000). Black dotted horizontal line at 0.5 AUC indicates random prediction, whereas an AUC value of 1 indicates perfect prediction. **D** Representative genomic regions showing comparison of pseudobulk and 10-HMM RT with or without aphidicolin treatment. 10-HMM RT fractions were ordered from S1 to S10, which represent early- to late-S phase. Aphidicolin-induced RT changes defined by 10-HMM RT is shown at the bottom of the figure. **E** Stacked bar chart showing the percentage of aphidicolin-induced RT changes in each untreated 10-HMM fraction. **F** Heatmap showing the average z-score values of pseudobulk RT, GC content, gene density, H3K4me3, H3K36me3, and A/B compartment score for aphidicolin-induced RT-change categories in each untreated HMM fraction of U2OS. 10-HMM RT fractions were ordered from S1 to S10, which represent early to late S-phase. *N*, number of 100-kb genomic bins. Statistics are provided in Supplementary Data 4. Source data are provided as a Source Data file.

Previous studies have primarily focused on the direction of RT changes following aphidicolin treatment, which was also one of our focuses. In addition, our results so far suggest that RT landscapes are predictive of aphidicolin-induced break classes (Figs. 3E, 4D, and Supplementary Fig. 8E). To test whether information encoded in RT landscapes can actually predict break classes, we performed a binary logistic regression analysis. Using the untreated U2OS RT profile, we derived local RT landscape features such as RT slope and RT curvature. Importantly, these features showed little to no correlation with RT values (Fig. 6A, B), suggesting that they capture independent aspects of the RT landscape shape rather than RT itself. We then generated three logistic regression models for each break class using (i) RT slope alone, (ii) RT curvature alone, and (iii) a combination of RT slope and curvature (see “Methods”). Model performance was evaluated using the “area under the receiver operating characteristic curve (AUC),” which quantifies the ability of each model to predict genomic regions that belong to a given break class from the rest of the genome.

As a result, non-break regions exhibited near-random AUC values between 0.5 and 0.52, indicating little to no predictive power for any of the three models (Fig. 6C). In contrast, CFS-L break regions were predicted with good accuracy (AUC = 0.7–0.78) across all three models (Fig. 6C). This predictive power of models incorporating both RT slope and curvature was consistent with the characteristic steep RT valleys observed at CFS-L break regions (Fig. 3E). In comparison, non-CFS-L RT-delayed break regions, which exhibited moderately steep RT valleys, showed AUC values slightly lower than those of CFS-L break regions (Fig. 6C). In addition, CFS-M and non-CFS-M RT-delayed (TTR class) break regions were predicted with moderate accuracy by curvature-only (AUC = 0.67) and slope-only (AUC = 0.66) models, respectively (Fig. 6C). Furthermore, non-CFS-E RT-advanced break regions were also predicted with moderate accuracy by the curvature-only model (AUC = 0.69, Fig. 6C), consistent with their enrichment for replication initiation sites (Fig. 5B). Taken together, these results demonstrate that RT landscape features in untreated cells, particularly slope and curvature, encode moderate but valuable predictive information that distinguishes distinct aphidicolin-induced break classes, independent of RT values.

### Genomic/epigenomic features in untreated cells can distinguish aphidicolin-induced RT changes

Conventionally, stress-induced RT delays have been associated with CFSs and chromosome breaks^8,9,20^. However, among non-CFS break classes, we found that chromosome breaks were also associated with regions showing advanced RT (Fig. 2C). While the link to RT advance and break formation is unclear in mid-late S-phase, we sought to determine what features distinguish genomic regions that delay or advance their RT in response to aphidicolin treatment. To investigate this, we first defined aphidicolin-induced RT advanced and delayed regions stratified by untreated RT states.

To this end, we utilized U2OS pseudobulk RT data to divide the S-phase into 10 fractions using a Hidden Markov Model (HMM), which we refer to as 10-HMM RT (see “Methods” and Supplementary Data 2). These fractions were labeled as S1 to S10, with S1 being the earliest and S10 the last-to-replicate genomic regions (Fig. 6D). Based on this 10-HMM RT, we identified genomic regions that shifted fractions (either earlier or later) following aphidicolin treatment as advanced or delayed RT regions. However, we could not define advanced and delayed RT for S1 and S10 fractions, respectively, as they represent the earliest and latest replicating fractions. Therefore, regions within S1 and S10 that did not change their RT after aphidicolin treatment were classified as uncategorized regions (Fig. 6D). The percentage of RT changes across each untreated HMM fraction suggested that approximately 50% of regions in each fraction remained unchanged, while the remaining 50% advanced or delayed their RT upon aphidicolin treatment (Fig. 6E). In addition, the location and the types of aphidicolin-induced RT-changes were mostly conserved between 5 and 10-HMM RT analysis (Supplementary Fig. 9A). Therefore, we decided to proceed with the aphidicolin-induced RT changes identified by 10-HMM RT and characterize them further.

After defining aphidicolin-induced RT advanced and delayed regions in S1 through S10 fractions, we performed a comprehensive characterization of these regions by calculating the z-score values of different genomic and epigenomic properties for RT advanced, unchanged, and delayed regions in each untreated HMM fraction. Interestingly, we found that the average RT of aphidicolin-induced RT advanced and delayed regions deviated from that of unchanged regions in every HMM fraction, not only upon aphidicolin treatment but even in the untreated condition (Fig. 6F and Supplementary Data 4). In addition, genomic and epigenomic features, such as GC content, gene density, H3K4me3, H3K36me3, and Hi-C A/B compartments, exhibited a similar trend and correlated more strongly with aphidicolin-treated RT than with untreated RT (Fig. 6F and Supplementary Data 4). Our data suggest that genomic regions exhibiting aphidicolin-induced RT delay or advance already differ in their genomic and epigenomic features in untreated cells, indicating an association between pre-existing chromatin states and subsequent RT responses to aphidicolin (Supplementary Note 10, Supplementary Fig. 9A–D, and Supplementary Data 4).

### Five classes of chromosome breaks are also observed in other human and mouse cells

Since U2OS is non-diploid and its karyotype is abnormal, we also analyzed a near-diploid human TERT-immortalized retinal pigment epithelial (hTERT-RPE1) and hTERT-fibroblast (BJ-hTERT) cells to see whether different classes of breaks in U2OS cells are conserved in other cell types. As single G1-phase cell data for both human cell lines treated with or without low-dose aphidicolin were publicly available^13^, CNV analysis was performed similarly to U2OS, and their karyotypes with or without aphidicolin treatment were compared. We found that RPE1 and BJ cells also increased their karyotype heterogeneity upon aphidicolin treatment (Supplementary Fig. 10A–C).

**Fig. 7:**
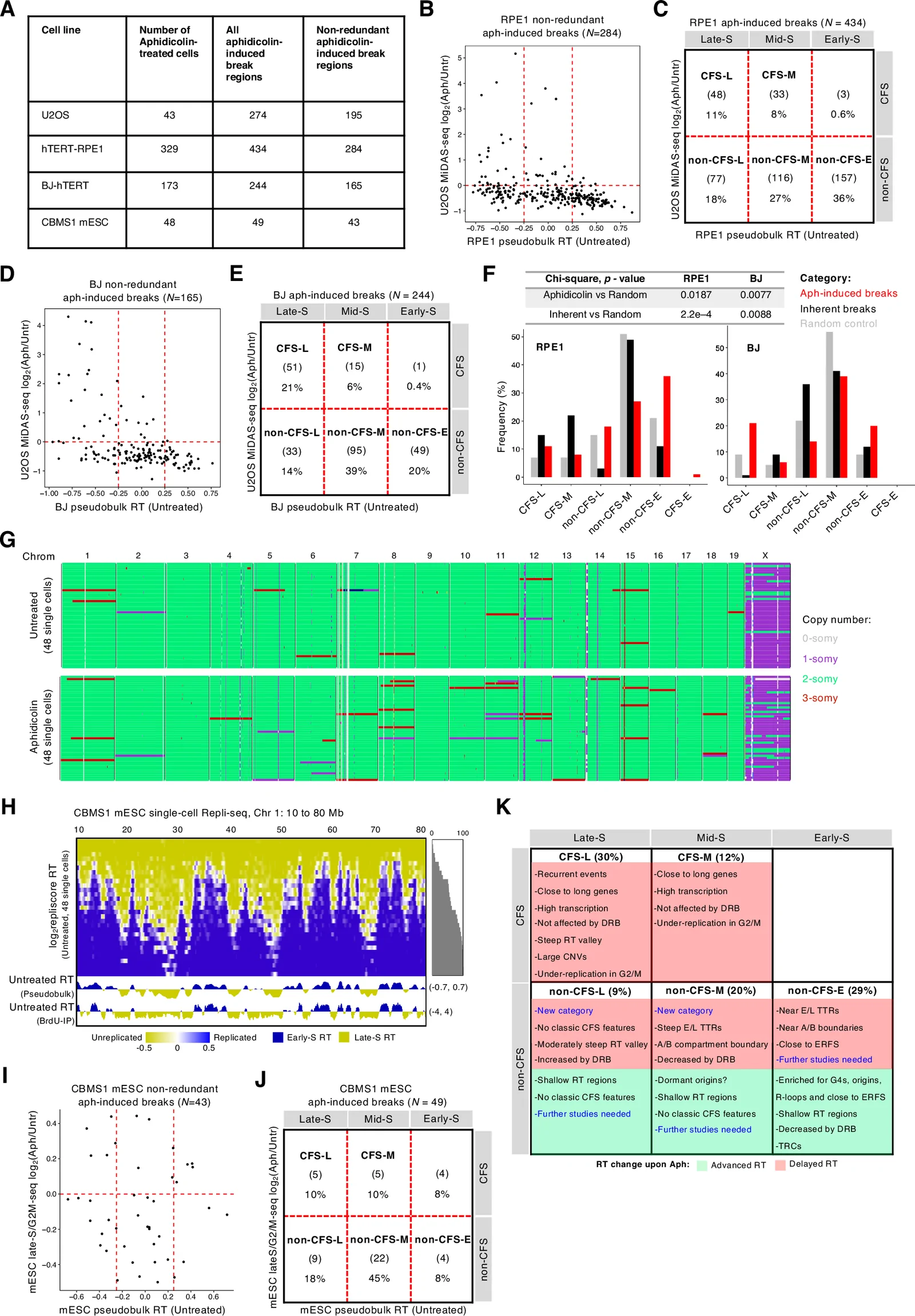
Five classes of breaks are also observed in other human and mouse cells. **A** Table showing the number of single cells treated with aphidicolin, and the number of all and non-redundant aphidicolin-induced break regions identified in human and mouse cells. **B** RPE1 non-redundant aphidicolin-induced break regions categorized using RPE1’s untreated pseudobulk RT and U2OS’s aphidicolin-induced MiDAS-seq signals. Vertical red dotted lines categorize the break regions into late, mid, and early-S RT. Horizontal dotted red line categorizes the break regions into CFS and non-CFS. **C** Percentage of RPE1’s aphidicolin-induced break regions in each class as defined in (**B**). Number within the bracket in each class is the total number of aphidicolin-induced breaks. **D** BJ’s non-redundant aphidicolin-induced chromosome break regions categorized using BJ’s untreated pseudobulk RT and U2OS’s aphidicolin-induced MiDAS-seq signals. Vertical red dotted lines categorize the break regions into late, mid, and early-S RT. The horizontal dotted red line categorizes the break regions into CFS and non-CFS. **E** Percentage of BJ’s aphidicolin-induced break regions in each class as defined in (**D**). Number within the bracket in each class is the total number of aphidicolin-induced breaks. **F** Bar chart showing the frequency of breaks (aphidicolin, red; inherent, black) and 1000 sets of randomized controls (grey) in each RT-MiDAS-defined break class for RPE1 and BJ cells. Exact *P*-values are shown from two-sided Chi-square test. Pairwise comparison *P*-values are shown in Supplementary Data 4. **G** Genome-wide copy number heatmap of single CBMS1 mESC G1-cells treated with or without low-dose aphidicolin. Copy number analysis was performed at 500-kb resolution, and each color represented a copy number. **H** Comparison of untreated single-cell log_2_repliscore RT heatmap, pseudobulk RT, and conventional early/late BrdU-IP RT (average of two replicates) of CBMS1 mES cells for a representative genomic region. Log_2_repliscore RT heatmap is ordered by the percentage replication score of each single cell (gray bars on right). **I** CBMS1 mESC non-redundant aphidicolin-induced chromosome break regions categorized using mESC’s untreated pseudobulk RT and mESC’s aphidicolin-induced late-S/G2/M-seq signals. Vertical red dotted lines categorize the break regions into late, mid, and early S RT. The horizontal dotted red line categorizes the break regions into CFS and non-CFS. **J** Percentage of mESCs aphidicolin-induced break regions in each class as defined in (**I**). Number within the bracket in each class is the total number of aphidicolin-induced breaks. **K** Summary of features, RT landscape, and potential mechanism of different break classes identified in U2OS. Source data are provided as a Source Data file.

We identified 434 aphidicolin-induced break regions from 329 single aphidicolin-treated RPE1 cells, with 284 having unique genomic positions. In BJ cells, 244 aphidicolin-induced break regions were identified from 173 single aphidicolin-treated cells, with 165 having unique genomic positions (Fig. 7A and Supplementary Data 1). When compared with U2OS, BJ and RPE1 cells had 19% and 21% aphidicolin-induced break regions overlapped or present within ±1 Mb of U2OS breaks, respectively (Supplementary Fig. 10D). Overlapped breaks were mostly CFS breaks in U2OS cells, suggesting that a subset of CFS breaks is conserved between different human cell types.

To categorize the break regions, untreated RT data from single RPE1 and BJ cells throughout the S-phase was reanalyzed^13^, and pseudobulk RT was generated similarly to U2OS’s 10-cell Repli-seq (Supplementary Data 2). Since U2OS MiDAS-seq had been shown to largely overlap with MiDAS-seq peaks of other human cell lines^9^, we categorized chromosome breaks in RPE1 and BJ using U2OS’s aphidicolin-induced MiDAS-seq and their respective untreated pseudobulk-RT (Fig. 7B, D). Interestingly, the results suggest that our observations were not specific to U2OS cells, as all five classes of chromosome breaks were also observed in both RPE1 and BJ near-diploid cells. The relative frequency of each class of breaks was different in different cell types (Fig. 7C, E).

Since U2OS’s MiDAS-seq was used to define aphidicolin-induced break categories for RPE1 and BJ cells, we compared the observed break frequencies with 1000 sets of randomized controls that were matched for both the number and size of breaks and assigned to break classes based on their position on the RT-MiDAS axes. We found that the distribution of break classes differed significantly from the randomized controls in both RPE1 and BJ cells (Fig. 7F and Supplementary Data 4 for pairwise comparisons). Similarly, distribution of inherent break classes also differed significantly from randomized controls in both in RPE1 and BJ cells (Fig. 7F). Despite the uncertainties in statistical tests due to the limited sample size, we did not detect any enrichment for copy number gains or losses that was commonly observed across all cell types analyzed (Supplementary Fig. 5E–G and Supplementary Data 4). Interestingly, large copy number gains and losses were frequently associated with CFS-L breaks in all human cell lines analyzed (Supplementary Fig. 5A–C; see median sizes). However, unlike U2OS cells, CFS-M breaks in RPE1 and BJ cells were not frequently associated with large CNVs.

In addition to re-analyzing publicly available near-diploid human cell lines, we also performed experiments using CBMS1 mouse embryonic stem cells (mESCs) with a near-diploid karyotype to see if our results are conserved in mice. CNV analysis of CBMS1 mESC single G1 cells was done the same way as in U2OS cells (Supplementary Fig. 11A). Genome-wide single G1 cells karyotype heatmap with or without aphidicolin treatment showed an increase in chromosome CNVs (Fig. 7G).

We identified 49 aphidicolin-induced break regions in 48 single aphidicolin-treated CBMS1 mESCs, with 43 having unique genomic positions (Fig. 7A and Supplementary Data 1). To categorize aphidicolin-induced break regions, we performed whole-S scRepli-seq by collecting single cells throughout S-phase (Supplementary Fig. 11A) and generated a pseudobulk RT profile using CBMS1 mESCs (Fig. 7H and Supplementary Data 2).

Because we do not have CFS information in mouse cells, we instead utilized late-S/G2/M-seq data from CBMS1 mESCs treated with or without aphidicolin to categorize the break regions (Supplementary Fig. 11B). To do so, we first tested if we could identify CFS breaks by using late-S/G2/M-seq instead of MiDAS-seq data in U2OS cells. Our results suggested that CFS breaks were largely conserved in U2OS cells even when MiDAS-seq data were replaced by late-S/G2/M-seq data (Supplementary Fig. 11C). Therefore, we defined aphidicolin-induced break regions in CBMS1 mESCs by using their untreated pseudobulk RT and aphidicolin-induced late-S/G2/M-seq data. As in U2OS and other human cell types, distinct classes of breaks were also observed in mESCs (Fig. 7I, J), and the distribution of both aphidicolin-induced and inherent break regions was significantly different from randomized controls (Supplementary Fig. 11D and Supplementary Data 4), suggesting that our observation is conserved in mice. In addition, similar to human cell lines, large copy number gains and losses were frequently associated with CFS-L breaks (Supplementary Fig. 5D). Furthermore, similar to U2OS, genomic regions that exhibited aphidicolin-induced RT-delay or advance deviated from the average genomic and epigenomic features within each untreated HMM fraction of CBMS1 mESCs (Supplementary Fig. 11E and Supplementary Data 4), suggesting the conservation of this feature between humans and mice.

## Discussion

To date, fragile site studies have been performed under different experimental conditions and have largely focused on CFSs. In this study, we performed an unbiased single-cell mapping and characterization of low-dose aphidicolin-induced chromosome break regions in mammalian cells. Using a single experimental condition in U2OS cells, we identified five classes of breaks based on RT-MiDAS axes. Specifically, 42% of breaks belonged to CFS-L and CFS-M break classes, which exhibited CFS features like late and mid RT, respectively (Fig. 7K). Both classes were associated with large CNVs and showed proximity to long genes, delayed RT, and under-replication upon aphidicolin treatment (Fig. 7K). Our pseudobulk RT accurately identified the aphidicolin-induced RT changes (Supplementary Fig. 3A–E), and we found that the degree of aphidicolin-induced RT delay of CFS-L and CFS-M break regions correlated well with MiDAS-seq signal intensity (Supplementary Fig. 6C), suggesting that the degree of aphidicolin-induced RT changes can be used to locate CFSs. Although CFS break classes showed high transcriptional activity, the level of aphidicolin-induced DNA damage at these regions did not change upon transcription inhibition. Our data suggest that transcriptionally active long genes with possibly low replication origin density^41^ remain under-replicated in G2/M and break during chromosome segregation, providing a potential mechanism for CFS-L and CFS-M break classes. The remaining 58% were non-CFS-L, non-CFS-M, and non-CFS-E break classes that replicated during late, mid, and early-S phase, respectively (Fig. 7K), and exhibited both advanced and delayed RT upon aphidicolin treatment. Non-CFS-L breaks were as late replicating as CFS-L breaks. However, they were non-recurrent and showed low transcription levels compared to CFS-L breaks with similar RT. Interestingly, in both untreated and aphidicolin-treated conditions, non-CFS-L RT-delayed breaks showed increased DNA damage upon transcription inhibition. These results indicated that non-CFS-L RT-delayed breaks represented a distinct break category that could be mechanistically different from CFS-L. In addition, transcription inhibition experiments suggested that, at non-CFS-L RT-delayed breaks, aphidicolin-induced replication stress and DRB-induced transcription stress may be less directly coupled, where transcriptional stress may represent the dominant source of DNA damage. Non-CFS-M RT-delayed break regions frequently overlapped with E/L-TTRs and A/B compartment boundaries and represented another distinct break category. Upon aphidicolin treatment, E/L-TTRs and non-CFS-M RT-delayed breaks regions showed increased G2/M replication, and DNA damage at these break regions was decreased upon transcription inhibition, suggesting transcription-dependent incomplete DNA replication as their potential break mechanism (Fig. 7K). Non-CFS-E RT-advanced breaks did not show under-replication in G2/M but were enriched for aphidicolin-induced FANCD2, R-loops, replication origins, and G-quadruplexes. DNA damage at non-CFS-E RT-advanced break regions were also decreased upon transcription inhibition, suggesting TRCs as their break mechanism (Fig. 7K). Taken together, our data reveal the features, relative frequency, and potential mechanistic differences of distinct classes of breaks in a single experimental setting, thereby providing a holistic view of sequences prone to breakage under replication stress.

There are debates over whether CFSs coincide with TAD boundaries^9,20^. Consistent with an earlier study^9^, we did not find TAD boundaries significantly close to our CFS break classes. The sole exception was non-CFS-E break regions, although the median distance difference relative to control was minimal (Supplementary Fig. 6D). On the other hand, we found that the RT/compartment landscape can detect under-replication in G2/M and break classes. For instance, steep late-S RT valleys tend to be more frequently under-replicated in G2/M and are characteristic feature of CFS-L breaks (Fig. 3E, F). Similar to the steep late-S RT valleys, mid-S RT E/L-TTRs and A/B compartment boundaries tend to be under-replicated in G2/M, which are features of non-CFS-M RT-delayed breaks (Fig. 4D, E). Lastly, in early-S, shallow early-RT/A-compartment regions tended to advance their RT upon aphidicolin treatment and break likely due to TRCs (Supplementary Fig. 8E). The correlation between shallow RT and aphidicolin-induced RT advancement was also observed in mid and late S-phase (Figs. 3E and 4D). Furthermore, our prediction models revealed that RT landscapes in untreated cells can modestly predict aphidicolin-induced break classes. Together, RT and A/B compartment landscapes can distinguish aphidicolin-induced RT behavior and break classes.

On top of the RT/compartment landscape, our fine resolution 10-HMM RT analysis, followed by a comprehensive characterization, revealed a genome-wide feature for aphidicolin-induced RT-advanced and delayed regions. In untreated U2OS cells, aphidicolin-induced RT delayed and advanced regions deviate from RT-unchanged regions in each HMM for their genomic and epigenomic features (Fig. 6F). Interestingly, similar results were also observed in CBMS1 mESCs (Supplementary Fig. 11E), suggesting the conservation of features for aphidicolin-induced RT-changing regions between humans and mice. These observations suggest that regions that change their RT upon aphidicolin treatment could also be distinguished by their genomic and epigenomic features in untreated cells.

We demonstrated that different classes of breaks observed in U2OS cells were also present in other near-diploid human and mouse cell lines. However, their frequency varied among cell lines. For instance, 42% were CFS breaks in U2OS cells, but it was only 19% for RPE1 cells. This difference may be related to a recent report suggesting that RPE1 cells tend to possess more robust replication and G2 checkpoints that allow them to prevent under-replication compared to cancer cell lines^42^. It should also be noted that we used U2OS MiDAS-seq data to identify CFS breaks in RPE1 cells. Further studies are necessary to clarify these observations.

As for recent reports on related topics, Shaikh and colleagues^13^ reported the first single-cell-level study of chromosomal CNVs. Their study focused on CFSs that were associated with active long genes and large CNVs. We showed that different classes of breaks can be identified from their data. Specifically, in addition to CFSs, our study identified and characterized non-CFS break classes that were not associated with large CNVs. Another recent study identified translocation breakpoints in mice using cell populations and found a category of breaks at TTRs, which they termed timing transition region fragile sites (TTRFS)^43^. While we also found a distinct class of breaks at TTRs (i.e., non-CFS-M RT-delayed regions), the majority of the TTRFSs had features of our CFS breaks, such as converging forks, and were closer to long genes compared to our non-CFS-M RT-delayed breaks (Supplementary Fig. 12A, B). Furthermore, when the mouse TTRFS coordinates were converted to human genomic coordinates, we found that only one out of 43 TTRFSs overlapped with our non-CFS-M RT-delayed break regions (Supplementary Fig. 12C). These results support the idea that our non-CFS-M RT-delayed (TTR class) breaks are distinct from TTRFS in their genomic features and distribution.

## Methods

### Cell culture

Human osteosarcoma cells (U2OS), kindly provided by Dr. Minoru Takata of Kyoto University, was cultured in DMEM (Nacalai, 08459-64) supplemented with 10% FBS (Sigma, 172012) and maintained at 37 °C with 5% CO_2_. CBMS1 mouse embryonic stem cells (mESCs) have been described^44^ and grown in 2i/LIF medium as described^45^. For flow cytometry, cells were fixed with 75% ethanol after single-cell suspension with trypsin (Nacalai, 32777-44).

### Optimization of aphidicolin treatment and EdU labelling duration

To ensure that all cells progress through one complete S-phase under aphidicolin treatment, we assessed the duration of a single S-phase in U2OS cells treated with or without low-dose aphidicolin (0.4 μM). To this end, a double thymidine block and release experiment was performed. Asynchronous U2OS cells at 40–50% confluency were treated with 2 mM thymidine (Sigma, T9250) for 14 h, then released into fresh medium for 9 h, followed by another 14-h treatment with 2 mM thymidine. Following double thymidine block, cells were released into fresh medium with or without 0.4 μM aphidicolin (Wako, 011-09811), and samples were collected at 8, 16, and 24 h post-release. Collected single-cell suspension was fixed with 75% ethanol, and their DNA was stained using propidium iodide (Nacalai, 29037) or FxCycle Violet (Thermo Fisher Scientific, F10347) for 15–30 min in the dark. The stained samples were then analyzed using flow cytometry (Sony SH800 or MA900).

For the 2D-FACS cell-cycle profiles, it’s noted that aphidicolin treatment influences the amount of EdU incorporation. Therefore, EdU labeling duration was optimized to observe a minimal sufficient separation between replicating and non-replicating cell populations. In the presence of 0.4 μM aphidicolin, cells were subjected to EdU labeling at 10 μM concentration for 15, 30, 60, and 120 min. For 2D-FACS cell-cycle profiles, labeled EdU was stained using the Click-iT EdU flow cytometry kit (Thermo Fisher Scientific, C10633) before DNA staining.

### Sample preparation for karyotype analysis using single G1 cells

For G1 karyotype and RT analysis, cells were treated with or without 0.4 μM aphidicolin for 24 h and labeled with 10 μM EdU for last 60 and 15 min, respectively. For single G1 karyotyping, single G1 cells were sorted from the 2D cell-cycle FACS profile into 0.2 ml 8-strip PCR tubes using a 100 μM sorting chip in single-cell mode. Sample preparations were carried out following the protocol outlined in ref. ^17^. In brief, after cell lysis, DNA was amplified utilizing a SeqPlex Kit (Sigma, SEQXE), and libraries were generated using an NGS LTP library preparation kit (KAPA, KK8232) for paired-end sequencing on the HiSeqX Ten system.

### Sample preparation for 10-cells or single-cell Repli-seq analysis

For single-cell RT analysis using CBMS1 mESCs, single cells throughout G1 and S phase were sorted. For U2OS, due to the observed karyotype heterogeneity, single-cell experiment was not performed. Instead, 10 cells from G1 and each of the 14 S-phase gates in the 2D cell-cycle FACS profile was sorted. Sample preparations followed the procedure outlined in ref. ^17^, similar to single-G1 karyotyping.

### Sample preparation for BrdU-IP Repli-seq and late-S/G2/M-seq analysis

For RT analysis using BrdU-IP Repli-seq and late-S/G2/M-seq, we followed the BrdU-IP protocol as described in ref. ^12^ with some modifications. Cells were treated with or without 0.4 μM aphidicolin for 24 h. Fifty micromolar BrdU was added to the medium 30 min before harvesting, and cells were fixed using 75% ethanol. After DNA staining with propidium iodide, early-S, late-S, and G2/M phase cells (at least 10,000 cells per fraction) was sorted from 1D cell-cycle FACS profile, and genomic DNA was extracted. Bioruptor UCD-250 (Sonic Bio) was used to fragment DNA (output, high; on/off time, 30 s; cycles, 5), and BrdU-incorporated DNA fragments was immunoprecipitated using anti-BrdU antibody (BD Biosciences Pharmingen, 555627). After BrdU-IP, immunoprecipitated DNA was subjected to whole genome amplification and NGS library preparation as described in sample preparation for single G1 and S phase cells.

### Quality control and mapping of NGS fastq files

We utilized one end of the paired-end sequenced reads as both ends of each read mostly overlapped and the cost of paired-end sequencing was low. Raw fastq files were subjected to quality assessment with FastQC v0.11.5, and the cutadapt program v1.15^46^ was used to eliminate SEQXE and Illumina adapters. SEQXE adapter was empirically determined as the sequence that repeatedly appeared near the 5′ end. Mapping to the hg19 (GRCh37; chr 1–22, X) or mm9 (NCBI Build 37; chr 1–19, X) genome was carried out using bwa v0.7.17-r1188 (bwa-aln command)^47^. The resulting mapped reads were sorted using samtools v1.7^48^, and duplicate reads were flagged using picard-tools v2.8.1.

### Analysis of published hTERT-RPE1 and BJ-hTERT single G1 cells

Sequencing data of single-G1 cells from hTERT-RPE1 and BJ-hTERT human cells treated with or without low-dose aphidicolin were available as public datasets (Supplementary Data 3). Mapping and post-mapping steps for the above-mentioned datasets were performed as described in the previous section.

### Computation associated with karyotype analysis using single G1 cells

After marking the duplicate reads, copy number analysis was performed in R v4.1.3 using the AneuFinder package v1.22.0^18^. Duplicate reads, reads overlapping with blacklist regions (available at https://github.com/Boyle-Lab/Blacklist), and reads with a mapping quality (MAPQ) below 10 were filtered out. Uniquely mapped reads were counted in each 500-kb genomic window, adjusted for GC content (GC.BSgenome = BSgenome.Hsapiens.UCSC.hg19 for humans and BSgenome.Mmusculus.UCSC.mm9 for mouse, method = “loess”), and copy number segmentation was performed using the “findCNVs” function (method = “edivisive,” *R* = 50) of the AneuFinder package.

For quality control (QC), multivariate clustering was performed, and clusters with spikiness values (bin-to-bin variation of read counts) greater than 1 were excluded. As a result, the number of single cells that passed QC was as follows: U2OS, 44/44 untreated and 43/45 aphidicolin-treated; hTERT-RPE1, 91/96 DMSO-treated and 329/372 aphidicolin-treated; BJ-hTERT, 93/96 DMSO-treated and 173/180 aphidicolin-treated; and CBMS1 mESCs, 48/48 untreated and 48/48 aphidicolin-treated.

Cell-to-cell karyotype heterogeneity was determined for each 500-kb genomic window using “karyotypeMeasures” function of AneuFinder package. Karyotype heterogeneity score (H) is defined as

1 $${Karyotype}\,{heterogeneity}\,{score},H=\frac{1}{{TN}}{\sum }_{t=1}^{T}{\sum }_{f=0}^{S}f\cdot {m}_{{{f}},{{t}}}$$

Where *m*_f,t_ is the number of cells with copy number state (*s*) at bin (*t*), and *S* is the total number of copy number states.

### Identification of chromosome break regions

In each single cell, chromosome breakpoints were defined as junctions between adjacent 500-kb bins with different copy number calls. Chromosome breakpoints with a 95% confidence interval (CI) in each single cell was determined using the “getBreakpoints” function, and refined breakpoints with their refined 95% CI were obtained using the “refineBreakpoints” function of the AneuFinder package. Refined 95% CIs (referred to as “chromosome break regions” or “break regions”) from all single cells were concatenated into a single file for both untreated and aphidicolin-treated condition. Break regions identified in aphidicolin-treated cells that did not overlap with, nor present within ±1 Mb of any break region in untreated condition were defined as aphidicolin-induced break regions. Conversely, break regions in aphidicolin-treated cells that overlapped with or were located within ±1 Mb of a break region in untreated condition were defined as inherent breaks.

### Log_2_repliscore, pseudobulk, and 10-HMM RT analysis

We followed our established procedure to generate binarized replication timing (RT) profiles for each of the 14 fractions of S-phase or single-cells under analysis, along with their respective percentage replication scores^17^. The percentage replication score of each S-phase fraction or single-cells was utilized to compute their raw, non-binarized replication timing profile, termed as “log_2_repliscore RT.” For each 100-kb genomic bin

2 $${{{\mathrm{log}}}}_{2}{repliscore}\,{{RT}}={{{\mathrm{log}}}}2\left(\frac{M}{\left(1-\frac{R}{100}\right)}\right)$$

where, *M* is mappability corrected read count per 100-kb genomic bin and *R* is percentage replication score. The log_2_repliscore RT profiles derived from the 14 S-phase fractions or single-cells underwent loess smoothing and were subsequently averaged to generate a pseudo-bulk RT profile.

For five or 10-HMM RT, a five or ten-state HMM was applied, respectively, to 100-kb pseudobulk RT data using the “Rhmm” package (http://cran.r-project.org/src/contrib/Archive/RHmm/). First, k-means (*k* = 5 or 10) analysis using all chromosome data of each sample was performed to determine the initial parameters of each state. Then, HMM was trained by the “HMMFit” command of the RHmm package with the Baum–Welch algorithm. To calculate the optimal hidden states sequence, Viterbi’s algorithm (“viterbi” command of RHmm package) was used for each trained HMM. We subdivided the genome into five or ten RT groups from early to late S according to the mean value of the distribution parameters in five or 10 HMM states, respectively.

### Computation associated with BrdU-IP Repli-seq analysis

After removing duplicate reads and filtering blacklist regions, we counted the reads and calculated reads per million for each 100-kb non-overlapping window in early- and late-S fractions. Then, RT is determined by calculating log2(early/late) for each 100-kb window. All the BrdU-IP RT profiles were quantile normalized before downstream analysis.

### Computation associated with late-S/G2/M-seq analysis

After removing duplicate reads and filtering blacklist regions, we counted the reads and calculated reads per million for each 100-kb non-overlapping window in untreated and aphidicolin-treated conditions. Then, aphidicolin-induced G2/M replication is determined by calculating log2(aphidicolin/untreated) for each 100-kb window.

### EU imaging

U2OS cells were grown on 12-mm coverslips, labeled with 5-ethynyluridine (EU) for 1 h, and fixed with 2% paraformaldehyde (PFA; Electron Microscopy Sciences, 15714) for 10 min. After three washes with phosphate buffered saline (PBS; Gibco, 18912-014), samples were permeabilized with 1% Triton X-100 (Nacalai, 35501-15) for 15 min. Coverslips were washed three times with PBS, and EU staining was performed using Click-iT RNA Alexa Fluor 488 imaging kit (Invitrogen, C10329). Following another PBS wash, samples were counterstained with DAPI (1 μg/ml; Roche, 10236276001) for 15 min. Coverslips were washed three times with PBS, air-dried, and mounted on glass slides using ProLong Diamond Antifade Mountant (Invitrogen, P36970). After curing for at least 24 h, imaging was performed using DeltaVision Olympus IX71 microscope equipped with an Olympus UPlanSApo 20× objective lens. Images were analyzed using Fiji software^49^. Nuclei were segmented, and EU intensity per nuclei was quantified.

### EdU and double immunostaining

U2OS cells were grown on 12-mm coverslips, labeled with EdU for 1 h, and fixed with 2% PFA. After permeabilization with 1% Triton X-100, EdU staining was performed using the Click-iT Plus EdU Alexa Fluor 647 Imaging Kit (Invitrogen, C10640). Coverslips were washed with PBS and blocked with Blocking One buffer (Nacalai, 03953-95) for 30 min. Samples were incubated with primary antibodies (rabbit anti-FANCD2, Abcam, ab108928; mouse anti-γH2AX, 20A8, gift from Dr. Hiroshi Kimura^50^) at 1:1000 dilution for 2 h at room temperature in the dark. Coverslips were washed three times with PBS and incubated with secondary antibodies (Cy3 donkey anti-mouse IgG (H+L), Jackson ImmunoResearch, 715-165-151; Alexa Fluor 488 donkey anti-rabbit IgG (H+L), Jackson ImmunoResearch, 711-545-152) at 1:1000 dilution for 1 h in the dark. After an additional PBS wash, samples were counterstained with DAPI for 15 min. Coverslips were washed three times with PBS and mounted on glass slides using ProLong Diamond Antifade Mountant. After curing for at least 24 h, imaging was performed on a DeltaVision Olympus IX71 microscope equipped with an Olympus UPlanXApo 60× oil-immersion objective lens. Images were deconvolved, and maximum-intensity projections were generated using algorithms in the SoftWorx package (Applied Precision), followed by analysis in Fiji. Nuclei were segmented, and DAPI and EdU intensities were quantified for each nucleus to determine cell-cycle phase. FANCD2 and γH2AX foci were masked and binarized for each nucleus, and the number of foci was quantified. Overlap between FANCD2 and γH2AX binarized foci was used to calculate the Jaccard index.

### Immuno-FISH

U2OS cells were grown on 35-mm glass-bottom dishes and fixed with 2% PFA. After cell permeabilization with 1% Triton X-100, samples were blocked with Blocking One buffer for 30 min. Cells were then incubated with primary antibody (rabbit anti-γH2AX, Abcam, ab81299) at 1:1000 dilution for 2 h at room temperature in the dark. Samples were washed three times with PBS and incubated with secondary antibody (Alexa Fluor 488 donkey anti-rabbit IgG (H+L), Jackson ImmunoResearch, 711-545-152) at 1:1000 dilution for 1 h in the dark.

For DNA-FISH, BAC probes (Supplementary Data 3) were individually labeled with fluorescent dUTP (Red-dUTP, Enzo Life Sciences, 02N34-050; or Cyanine 5-dUTP, Perkin Elmer, NEL579001EA) by nick translation (Abbott Molecular, 07J00-001). On the day of DNA-FISH, labeled DNA probes, human Cot-1 (Invitrogen, 15279-101), and salmon sperm DNA (Thermo Fisher Scientific, 15632-011) were ethanol-precipitated, resuspended in 100 μl hybridization buffer (20% dextran sulfate, 2× SSC, 50% formamide), and denatured at 80 °C for 10 min before hybridization. Immunostained samples on 35-mm glass-bottom dishes were washed three times with PBS and refixed with 2% PFA for 10 min. Samples were washed three times with PBS, permeabilized using 0.5% Triton X-100, and washed three times with PBS. Samples were treated with 0.1 M HCl for 5 min at room temperature, and then treated with 0.1 mg/ml RNaseA for 45 min at 37 °C. After washing three times with 2× saline-sodium citrate (SSC) buffer, samples were incubated with 50% formamide at 47 °C for at least 20 min Cellular DNA was denatured by placing the dishes on an 83 °C water-filled heat block for 4 min. After removing the dish from the heat block, formamide was removed immediately, and 100 μl of denatured probe mixture in hybridization buffer was added. An 18-mm coverslip was placed, and the dishes (samples) were incubated overnight at 37 °C in a humidified chamber in the dark. The next day, coverslips were removed, and samples were washed twice with prewarmed (50 °C) 2× SSCT (2× SSC, 0.1% Tween-20) for 15 min, followed by two additional washes with 2× SSCT for 1 h at room temperature in the dark. After washing once with 2× SSC and once with PBS, samples were counterstained with DAPI for 15 min, washed three times with PBS, and mounted with ProLong Diamond Antifade Mountant. An 18-mm coverslip was placed on top. After curing for at least 24 h, imaging was performed on a DeltaVision Olympus IX71 microscope equipped with an Olympus UPlanXApo 60× oil-immersion objective lens. Images were deconvolved, and maximum-intensity projections were generated using algorithms in the SoftWorx package (Applied Precision) and analyzed in Fiji. Nuclei were segmented, and γH2AX intensity in each nucleus was quantified. DNA-FISH signals were identified using the “Find Maxima” function in Fiji with a noise tolerance threshold of 3000, which was determined empirically to reliably detect true FISH foci while minimizing background noise. In addition, given the hypertriploid nature of U2OS cells, we applied a filtering step to include only nuclei with 2–8 FISH foci for downstream analysis. γH2AX signal intensities at each FISH spot were calculated and averaged per nucleus. To remove batch effects, averaged γH2AX signals were normalized to the mean nuclear γH2AX intensity of untreated cells within the same experiment. The normalized values were then log_2_-transformed, to facilitate interpretation in terms of relative (fold) changes with respect to the untreated baseline at zero. The reported values therefore represent log_2_-transformed γH2AX intensities at FISH spots relative to the untreated baseline.

### Analysis of published GRO-seq, DRIP-seq, NS-seq, ChIP-seq, and MiDAS-seq data

We performed reanalysis of the published GRO-seq, NS-seq, EdU-seq, DRIP-seq, ChIP-seq, and MiDAS-seq data for U2OS cells (Supplementary Data 3). After removing duplicate reads, blacklist filtering was performed, and reads with a MAPQ > = 10 was selected. Reads per million (RPM) for every 100-kb genomic bin were calculated. For comparison purposes, the log2 ratio of enrichment values between untreated and aphidicolin-treated samples was calculated for each bin (log2(aphidicolin/untreated)). In cases where only untreated samples were available, the log2 ratio of immunoprecipitation (IP) per input was computed for each 100-kb genomic bin.

### Analysis of published Hi-C data

We performed reanalysis of the published U2OS and CBMS1 mESC untreated Hi-C data (Supplementary Data 3)^23^. HiC-Pro v3.1.0 with default parameters was used to generate contact matrix from fastq files^51^ (*genome = mm9 or hg19, min_MAPQ* = *10, Genome fragment = HindIII or DpnII*), and A/B compartment profile was calculated using Cooltools v0.5.4^52^ (*cooltools eigs-cis $cool --phasing-track $GC*, where $GC is GC content in 100-kb non-overlapping windows).

### Identification of TAD boundaries

TAD boundaries were identified as described^27^ using a custom script. Valid pair files obtained from HiC-Pro were converted to 50-kb cool files, and the insulation score was calculated (options: -is 250000 -ids 0 -im “mean” -bmoe 0 -nt 0). Boundary scores were calculated from the insulation score, and the local max 50-kb bin in continuous positive regions was defined as a putative TAD boundary.

### Calculation of DNA free energy

Genome-wide DNA structure prediction for hg19 was performed using ViennaRNA^53^ package as described^54^. Five-kb genomic windows with 1-kb sliding windows were generated for hg19, and blacklist regions were removed. For the above-mentioned genomic bins, DNA structure was predicted using RNAfold command (*--paramFile=dna_mathews2004.par --jobs* = *30 --noGU --noLP --gquad --noPS --noconv*).

### Generation of randomized control regions

Randomized control regions were generated using bedtools “shuffle” command (*bedtools shuffle -noOverlapping -chrom -incl -seed $seed -g $genome -i $breaks*). $breaks is aphidicolin-induced chromosome break regions, $seed is set from 1 to 100 or 1000 to generate 100 or 1000 sets of randomized control, respectively, and $genome is hg19 genome binned into 1-Mb bins. $genome bins overlapping with hg19 blacklist regions and aphidicolin-induced chromosome break regions were removed. After generating 100 or 1000 sets of randomized control, they were concatenated and categorized into early/mid/late-S phase using untreated pseudobulk RT in a manner similar to break categorization. Notably, the RT distribution of breaks and randomized controls in early-, mid-, and late-S phases were similar and showed no significant difference (Supplementary Fig. 6A).

### Identification of DNA replication termination zones

Local minima of the RT profile (i.e., termination zones, TZs) were identified using pseudobulk RT and a custom script. Briefly, a 100-kb bin was defined as a local minimum if it was flanked on both sides by at least three consecutive bins forming a descending ladder pattern toward the minimum. Once TZs were identified genome-wide, they were categorized as early-, mid-, or late-S based on untreated pseudobulk RT. Heatmaps and average line plots surrounding the TZs or break regions were obtained using deepTools^55^.

### Identification of timing transition regions

Timing transition regions (TTRs) were defined using 100 kb untreated pseudobulk RT profile. Briefly, absolute slope values of the RT profile was calculated for every 100 kb bins. Then, regions with absolute RT slope values greater than genome-wide mean were defined as TTRs.

### Binary logistic regression analysis

To test whether information encoded in the RT landscape is sufficient to predict aphidicolin-induced break classes, we performed a binary logistic regression analysis using the U2OS untreated RT landscape features (slope and curvature). Briefly, genome-wide RT data at 100-kb resolution were annotated with break classes by overlapping them with experimentally defined aphidicolin-induced break regions. Bins overlapping a break were assigned the corresponding break class, while all other bins were labeled as “no break regions.” To quantify the local RT landscape, we computed the RT slope (first derivative of RT) and RT curvature (second derivative of RT) from the RT profiles. These features were calculated using 100-kb sliding windows to capture the local RT landscape.

3 $${Absolute}\,{RT}\,{slope}=\left|\frac{{{RT}}_{i+1}-{{RT}}_{i-1}}{2\Delta x}\right|$$

4 $${RT}\,{curvature}=\frac{{{RT}}_{i+1}-{2{RT}}_{i}\,+{{RT}}_{i-1}}{{\left(\Delta x\right)}^{2}}$$

Where *i* represents a given 100-kb genomic bin and Δ*x* represents bin size (Δ*x* = 100 kb). We then evaluated whether RT landscape features could predict the break class identity using a one-versus-rest binary logistic regression framework (*glm(model, family = binomial, data* = *RT)*), applied separately to each break class. Three models were tested: (i) slope-only, (ii) curvature-only, and (iii) slope + curvature. Model performance was quantified using the “area under the receiver operating characteristic curve (AUC),” which measures the ability of the models to discriminate 100-kb regions belonging to a given break class from all other bins in the genome. An AUC value of 0.5 indicates random prediction, whereas a value of 1 indicates perfect prediction. To assess the uncertainty of each AUC estimate, we computed 95% confidence intervals using a stratified bootstrap procedure (*N* = 1000). The 2.5th and 97.5th percentiles of the resulting bootstrap AUC distribution were taken as the lower and upper bounds of the 95% confidence interval, respectively.

### Type and size of CNVs associated with break regions

Type (loss or gain) and size of CNVs were determined for each aphidicolin-induced chromosome break regions. For each break regions, most frequent copy number in untreated single cells was identified. Then, at each break regions of aphidicolin-treated single cells, type and size of copy number segments that varied from most frequent copy number was determined.

### Identification and liftOver of ERFSs

As defined in original article^10^, overlapping regions among ChIP-seq peaks of mouse RPA, BRCA1 and SMC5 were identified as ERFSs. Coordinated of mouse ERFSs (mm9) was converted to hg19 using “liftOver” command (-minMatch=0.8).

### Statistics and reproducibility

Statistical tests were performed using rstatix v0.7.2 package in R. Statistical tests used, exact values of *P* and Pearson’s correlation values (*R*) are reported in the figures, figure legends, or associated main texts. Statistical significance is determined by the value of *P* < 0.05 by the indicated tests. In all the boxplots, the whiskers (lines extending from the box) represent the minimum and maximum values in the dataset, excluding any outliers (1.5× interquartile range). The horizontal bar at the center of the box represents the median or 50th percentile of the data. The lower and upper bounds of the box indicate the 25th and 75th percentiles, respectively. *N* is the sample size.

Experimental reproducibility was demonstrated as follows. Imaging, 10-cell Repli-seq, BrdU-IP Repli-seq, and late-S/G2/M-seq were performed with at least two biological replicates. Single-cell experiments were conducted in two separate batches following cell sorting. Sorted cells were stored in single-cell lysis and fragmentation buffer (Sigma, L1043) at –80 °C.

### Reporting summary

Further information on research design is available in the Nature Portfolio Reporting Summary linked to this article.

## Supplementary information

Supplementary Information

Description of Additional Supplementary Files

Supplementary Data 1

Supplementary Data 2

Supplementary Data 3

Supplementary Data 4

Reporting Summary

Transparent Peer Review File

## Source data

Source Data

## Data Availability

The scRepli-seq and BrdU-IP-seq data generated in this study have been deposited in NCBI’s Gene Expression Omnibus (GEO) database under accession code GSE310972. Processed data files and accession codes of previously published data are provided in Supplementary Data 2 and 3, respectively. Source data are provided with this paper.

## Code availability

Custom codes used in this study are available on GitHub (https://github.com/jothivanan-elumalai/single_cell_genome_fragility)^56^.
