# Supplementary material for: Single-cell mapping of chromosome breaks identifies multiple fragile site classes with distinct DNA replication timing landscapes
Source: Nat Commun. 2026 Sep 23;17:9055. doi: 10.1038/s41467-026-76451-1 (PMC13601574; doi:10.1038/s41467-026-76451-1)
Supplement: Supplementary file 1 [file 41467_2026_76451_MOESM1_ESM.pdf]

Supplementary Information

Single-cell mapping of chromosome breaks identifies multiple fragile site classes with distinct DNA replication timing landscapes

Jothivanan Elumalai[1,2] and Ichiro Hiratani[1,2,*]

[1]Laboratory for Developmental Epigenetics, RIKEN Center for Biosystems Dynamics Research (BDR), Kobe, Japan.

[2]Department of Developmental Biology and Regenerative Medicine, Division of Physiology and Cell Biology, Graduate School of Medicine, Kobe University, Kobe, Japan.

[*]Email: ichiro.hiratani@riken.jp

List of Supplementary Information

- Supplementary Figures 1 to 12
- Supplementary Notes 1 to 10
- Supplementary References

Supplementary Figures

Supplementary Figure 1

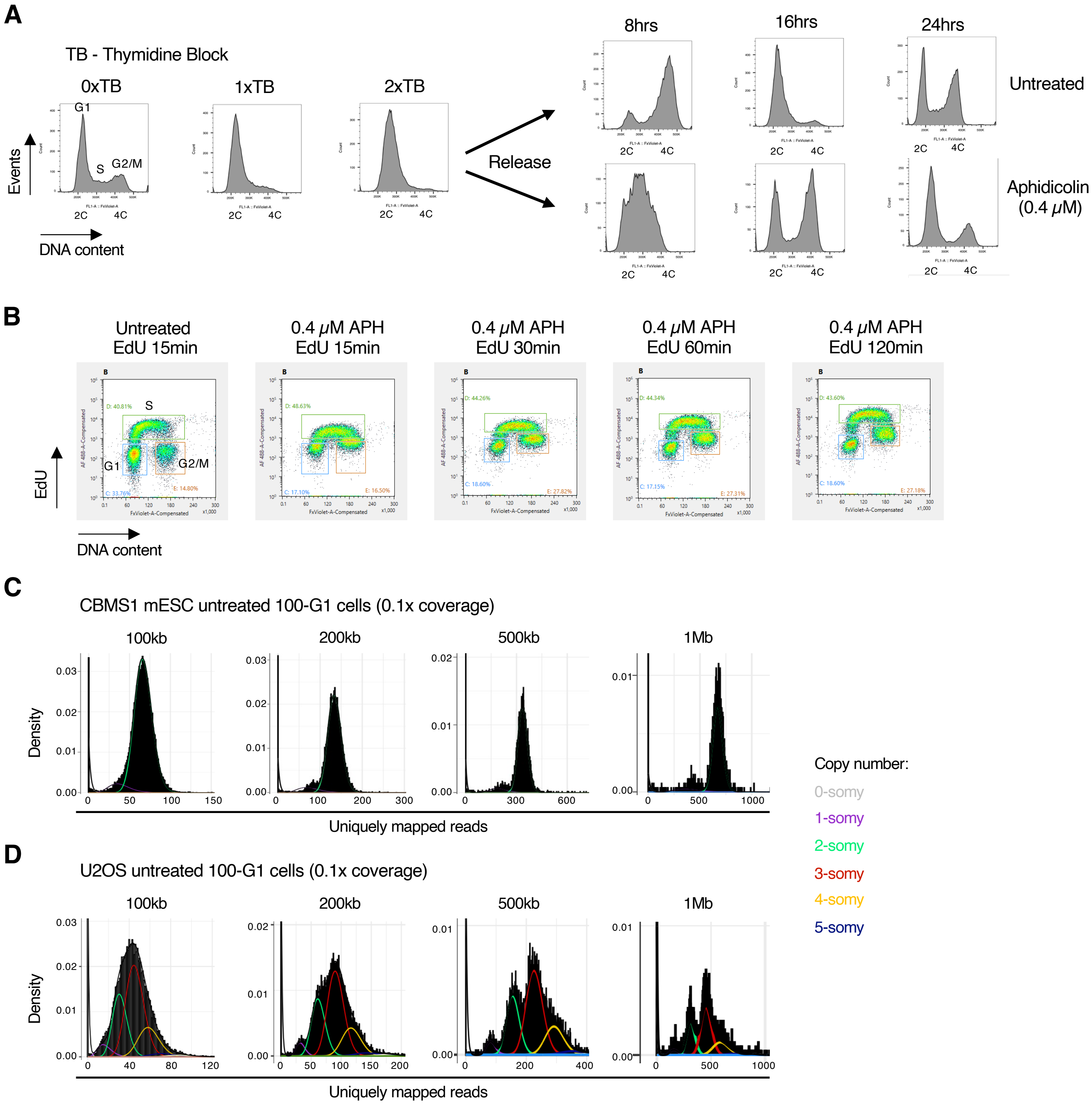

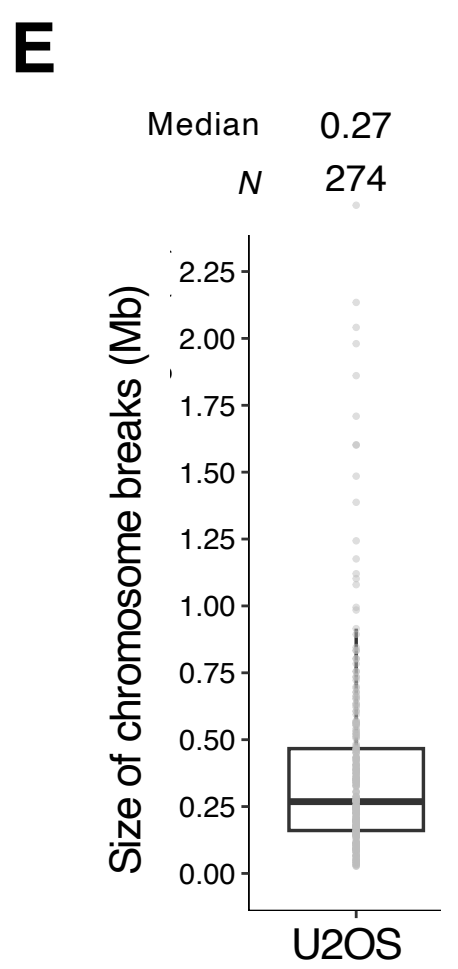

F

	Compared to		
	**MiDAS-seq CFSs**	**Cytogenetic CFSs**	**APH-induced breaks**
**APH-induced breaks**	38% (103 / 274)	45% (122 / 274)	-
**MiDAS-seq CFSs**	-	42% (137 / 324)	16% (53 / 324)
**Cytogenetic CFSs**	69% (59/ 86)	-	54% (46 / 86)

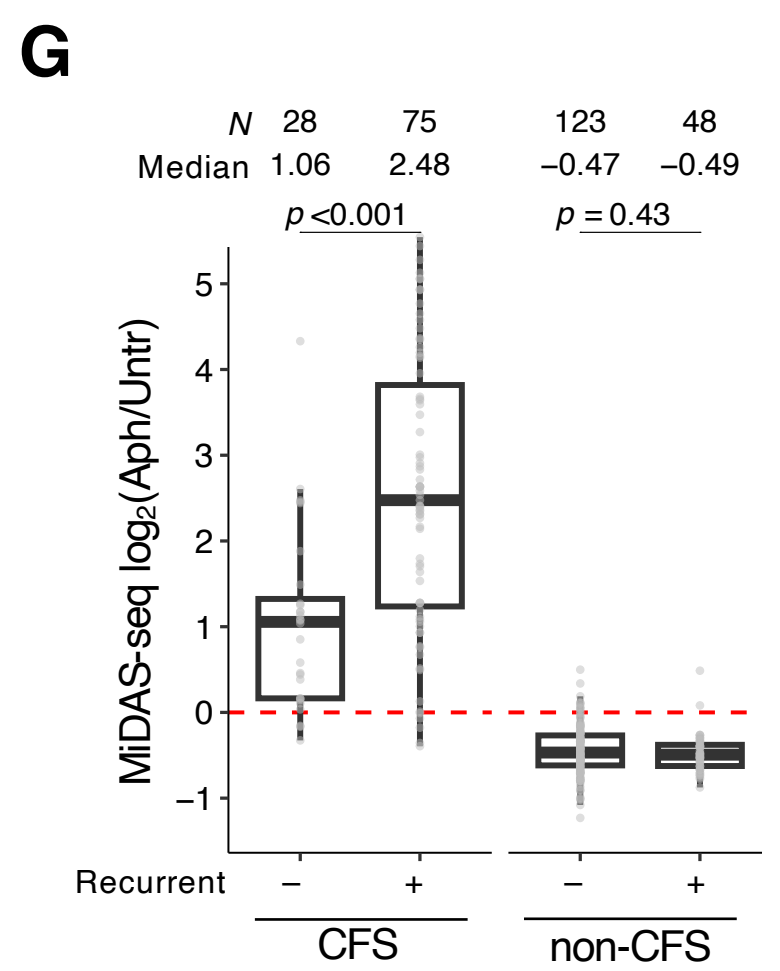

(Figure legend in next page)

Supplementary Figure. 1 | Chromosome copy number and break identification in U2OS

(A) Double thymidine block (TB) and release of U2OS cells treated with or without low-dose aphidicolin. FxCycle Violet dye was used as a marker for DNA content (2C – G1 and 4C – G2/M). FACS plot of U2OS cells collected at different time points before and after release from double thymidine block is shown. One cell cycle duration is defined as the time it takes to return to a 2C DNA content after release from a double thymidine block. **(B)** EdU labeling optimization experiment for U2OS cells to clearly separate replicating and non-replicating populations. A 2D cell-cycle FACS plot of U2OS cells treated with or without aphidicolin and collected after different EdU labeling durations is shown. **(C)** Uniquely mapped read density plots of 100 G1 untreated CBMS1 mESCs at different bin sizes. Colored lines are copy number calls from AneuFinder (1-somy, purple; 2-somy, green). **(D)** Uniquely mapped read density plots of 100-G1 untreated U2OS cells at different bin sizes. Colored lines are copy number calls from AneuFinder (1-somy, purple; 2-somy, green; 3-som, red; 4-somy, yellow). **(E)** Size range of aphidicolin-induced chromosome break regions in U2OS cells. *N*, number of aphidicolin-induced chromosome break regions. **(F)** Table showing the pairwise overlap percentage among our aphidicolin-induced breaks, MiDAS-seq's CFSs and cytogenetic CFSs. **(G)** Aphidicolin-induced MiDAS-seq signal for recurrent and rare breaks in CFS and non-CFS categories. CFS and non-CFS categorization was based on the overlap of 274 aphidicolin-induced breaks with 324 previously defined CFS regions. *P*-values were obtained from a two-sided unpaired Wilcoxon test. *N*, number of aphidicolin-induced chromosome break regions. For all box plots in this figure, the center line indicates the median, the box limits indicate the 25th and 75th percentiles, and the whiskers extend to the minimum and maximum values excluding outliers (1.5 x interquartile range).

Supplementary Figure 2

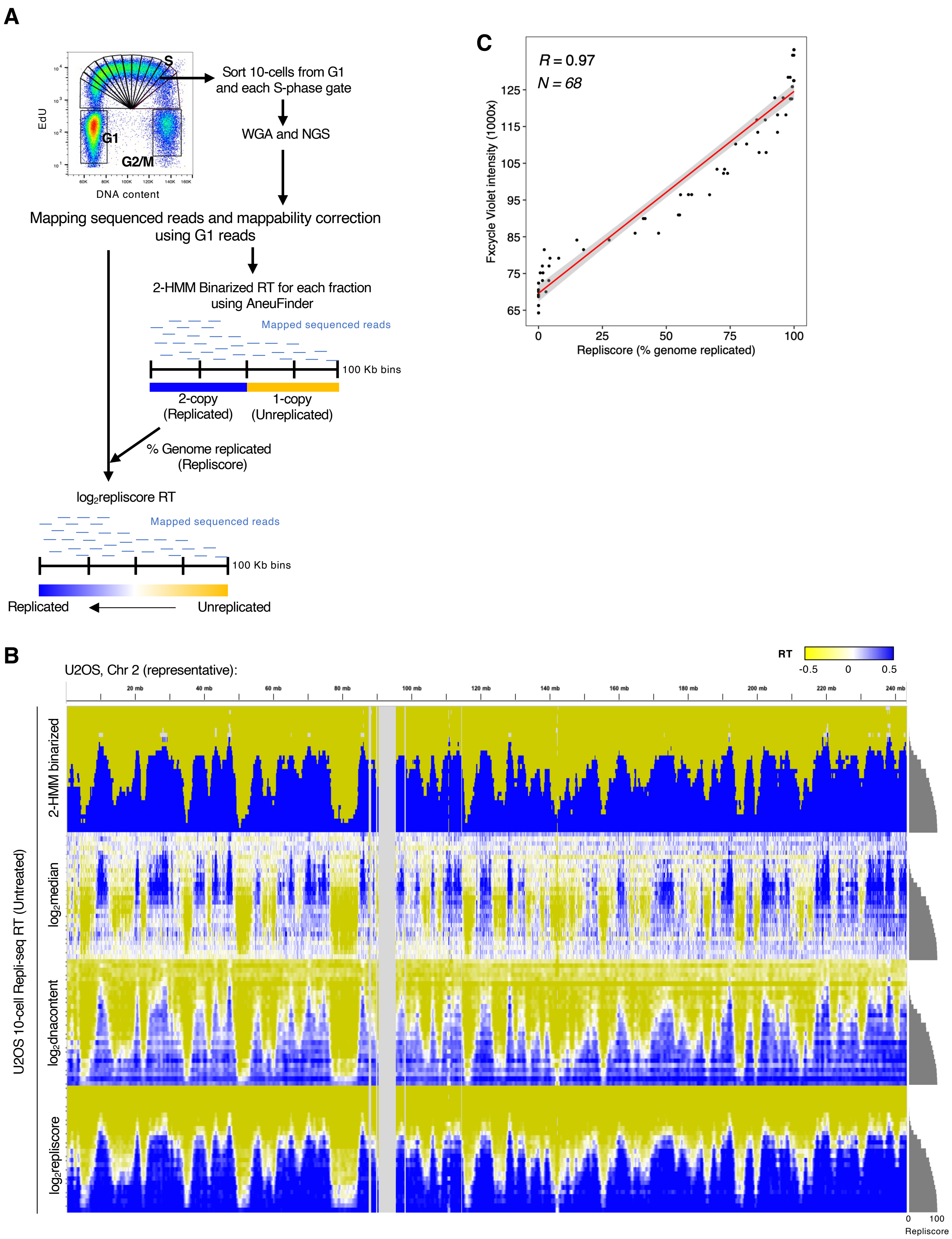

(Figure legend in next page)

Supplementary Figure. 2 | Generation of log_2repliscore RT profiles

(A) Steps involved in 10-cells Repli-seq experiment and log_2repliscore RT calculation. **(B)** Comparison of U2OS's untreated binarized, log_2median, log_2DNAcontent, and log_2repliscore RT. RT heatmaps were ordered by the percentage replication score (Repliscore) of each 10-cell fraction (gray bars on right). **(C)** Scatter plot showing the correlation between the repliscore and DNA content. DNA content is obtained as FxCycle Violet dye intensity from FACS metadata. Pearson's correlation (R) and N, number of 10-cell fraction from both untreated and aphidicolin treatment.

Supplementary Figure 3

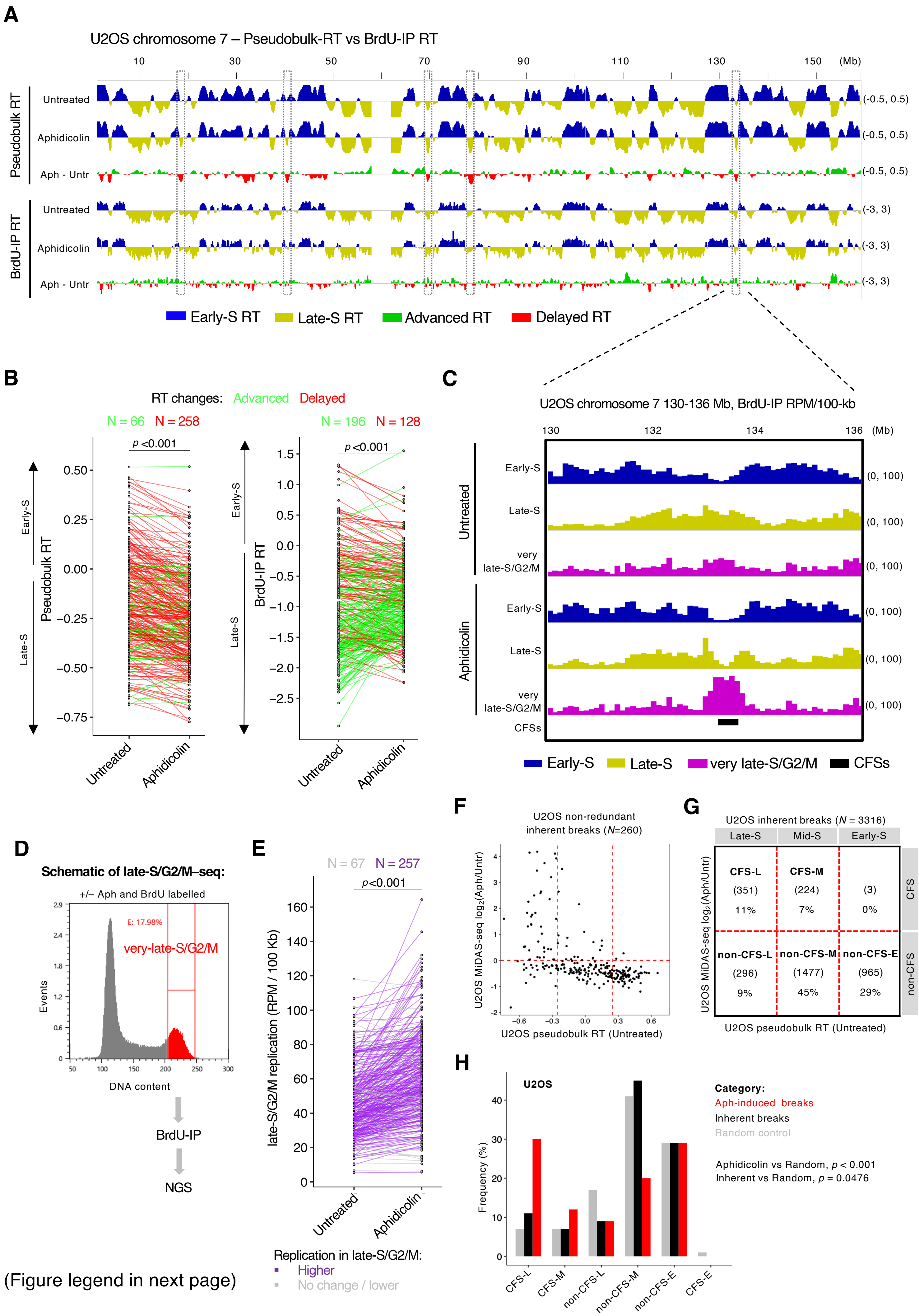

(Figure legend in next page)

Supplementary Figure. 3 | Pseudobulk RT and conventional BrdU-IP E/L RT

(A) Representative genomic region showing U2OS's 10-cell pseudobulk and conventional two-fraction BrdU-IP RT with or without aphidicolin treatment and RT difference (Aphidicolin – Untreated). Dotted segments show regions where RT is delayed and advanced upon aphidicolin treatment using pseudobulk and BrdU-IP RT, respectively. **(B)** Paired dot plot comparing pseudobulk RT (left) and BrdU-IP RT (right), with or without aphidicolin treatment for CFSs identified by MiDAS-seq. Lines represent the direction and degree of RT change (delayed, red; advanced, green) upon aphidicolin treatment. *P*-values were obtained from a two-sided paired Wilcoxon test. *N*, number of CFSs. **(C)** Representative genomic region showing U2OS's BrdU-IP raw reads count per million reads in each 100-kb genomic bin for early-S (blue), late-S (yellow), and very-late-S/G2/M (magenta) in untreated and aphidicolin treated conditions. **(D)** Experiment design of late-S/G2/M-seq. **(E)** Paired dot plot showing BrdU incorporation rate in late-S/G2/M with or without aphidicolin treatment for CFSs identified by MiDAS-seq. Lines represent the direction and degree of BrdU incorporation (higher, purple; no change/lower, grey) upon aphidicolin treatment. *P*-values were obtained from a two-sided paired Wilcoxon test. *N*, number of CFSs. **(F)** Non-redundant inherent chromosome break regions in U2OS cells were categorized using their untreated pseudobulk RT and aphidicolin-induced MiDAS-seq signals. Vertical red dotted lines categorize the break regions into late, mid, and early S RT. The horizontal dotted red line categorizes the break regions into CFS and non-CFS. **(G)** Percentage of U2OS's inherent break regions in each class, as shown in Supplementary Fig. 3F. The number within the bracket in each class is the total number of inherent breaks. **(H)** Bar chart showing the frequency of breaks (aphidicolin, red; inherent, black) and 1,000 sets of randomized controls (grey) in each RT–MiDAS-defined break class for U2OS. *P*-values were obtained from Chi-square test. Pairwise comparison *P*-values are shown in Supplementary Data 4.

Supplementary Figure 4

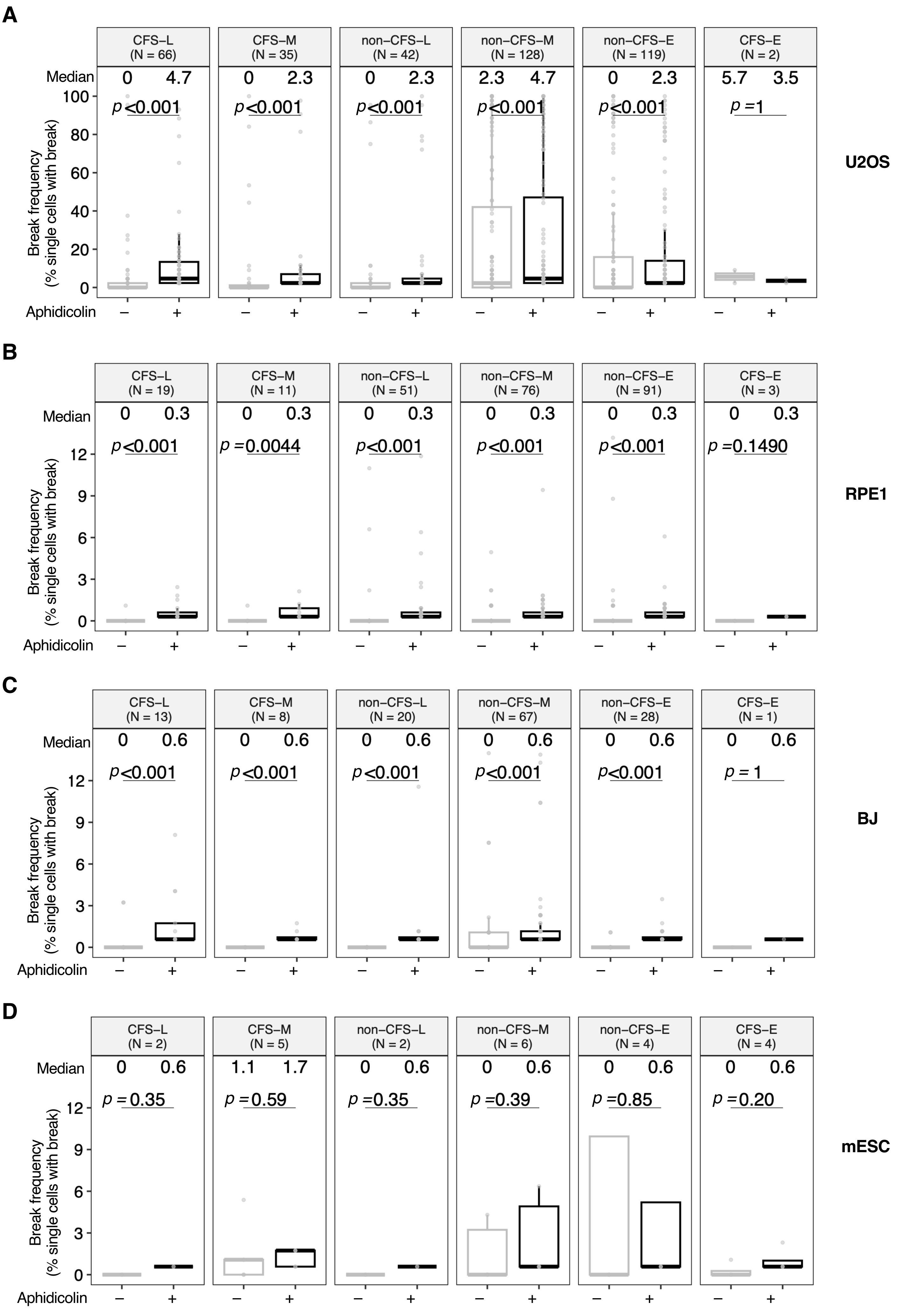

(Figure legend in next page)

Supplementary Figure. 4 | Aphidicolin treatment increases break frequencies across different break classes

(A–D) Boxplots overlaid with jittered points showing the distribution of break frequencies for break regions identified in aphidicolin-treated cells in U2OS **(A)**, RPE1 **(B)**, BJ **(C)**, and mESCs **(D)**. Each dot represents the break frequency of an individual break region. Briefly, break regions identified in aphidicolin-treated cells were categorized according to their positions on the RT-MiDAS axes. For each break region, break frequency was calculated independently in untreated (grey) and aphidicolin-treated (black) datasets as the percentage of single cells carrying a chromosome break within that region. See also Supplementary Note 5. Break frequencies were compared using a two-sided paired Wilcoxon test. *P*-values and median break frequency values are indicated above the boxplot. N, number of break regions. For all box plots in this figure, the center line indicates the median, the box limits indicate the 25th and 75th percentiles, and the whiskers extend to the minimum and maximum values excluding outliers (1.5 x interquartile range).

Supplementary Figure 5

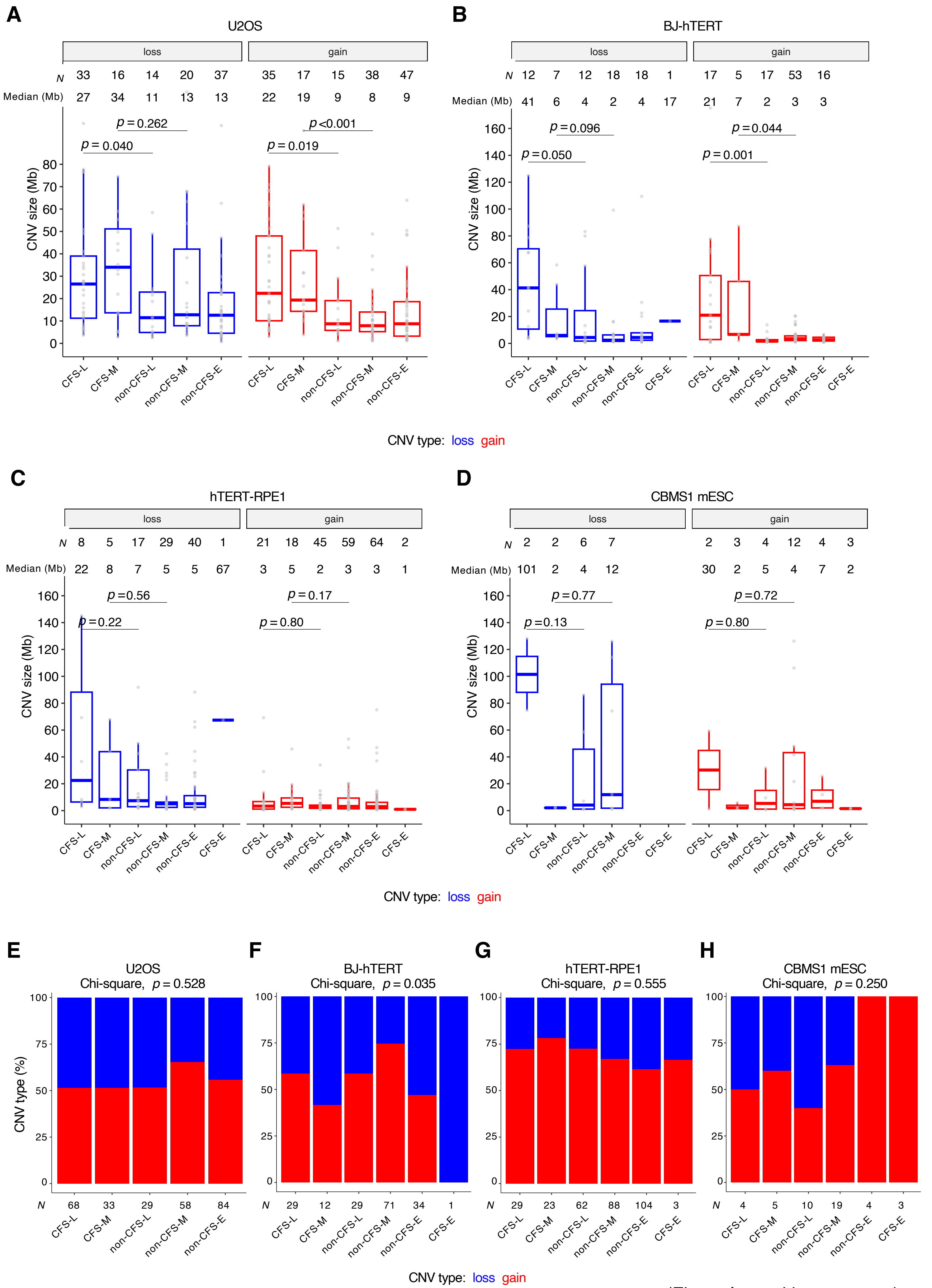

(Figure legend in next page)

Supplementary Figure. 5 | Type and size of CNVs associated with break classes

(**A-D**) Boxplot with jitters showing the size of both loss (blue) and gain (red) CNVs associated with each break classes in different cell types. *P*-values were obtained from a two-sided paired Wilcoxon test. *N*, number of CNVs associated with each break classes. (**E-H**) Stacked bar chart showing the type of CNV (loss – blue; gain – red) associated with each break classes in different cell types. *P*-values were obtained from Chi-square test. Pairwise comparison *P*-values are shown in Supplementary Data 4. *N*, number of CNVs associated with each break classes. For all box plots in this figure, the center line indicates the median, the box limits indicate the 25th and 75th percentiles, and the whiskers extend to the minimum and maximum values excluding outliers (1.5 x interquartile range).

Supplementary Figure 6

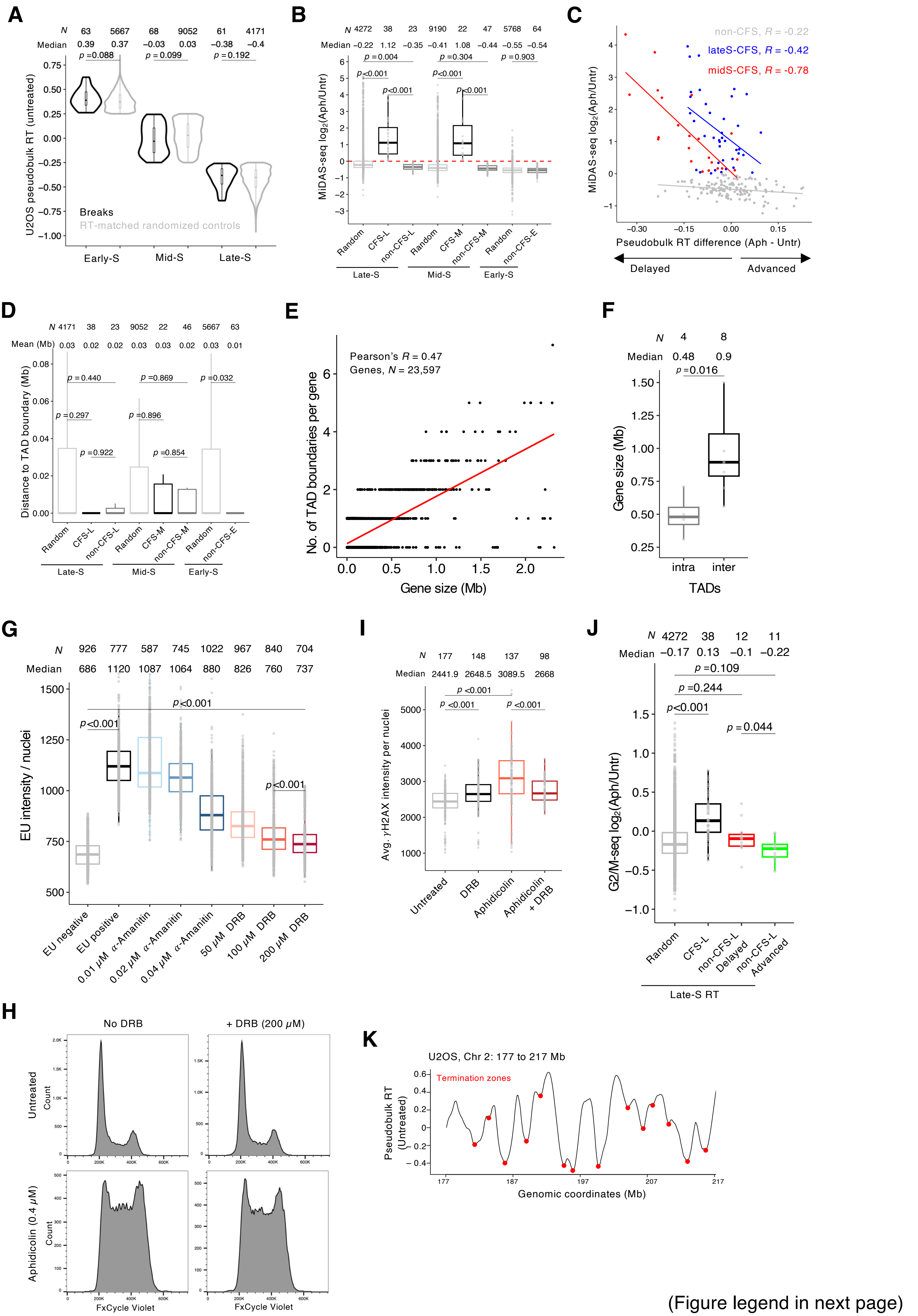

(Figure legend in next page)

Supplementary Figure. 6 | Characterization of late-RT breaks

(A) RT distribution of RT-matched randomized controls and breaks in early-, mid-, and late-S phases in U2OS. *P*-values were obtained from two-sided unpaired t-test. *N*, number of breaks or randomized controls. **(B)** MiDAS-seq values for breaks in each class (CFS, black; non-CFS, dark gray) and RT-matched randomized controls (light gray). *P*-values were obtained from two-sided unpaired Wilcoxon test. *N*, number of breaks or randomized controls. **(C)** Correlation between pseudobulk RT difference and aphidicolin-induced MiDAS-seq at CFS-L, CFS-M, and non-CFS break regions. Pearson's correlation (*R*) is shown, and each dot is a chromosome break region. **(D)** Distance to the nearest TAD boundary from break regions in each class (CFS, black; non-CFS, dark gray) and RT-matched randomized controls (light gray). *P*-values were obtained from two-sided unpaired Wilcoxon test. *N*, number of breaks or randomized controls. **(E)** Pearson's correlation (*R*) between gene size and number of TAD boundaries overlapped. *N*, number of genes. **(F)** Gene size of long genes that are present (black) or not present (grey) at a TAD boundary as defined by Sarni et al[20]. *P*-values were obtained from two-sided unpaired Wilcoxon test. **(G)** EU intensity per U2OS nuclei in different treatment conditions (no treatment with or without EU, different concentration of α-Amanitin and DRB treatment with EU). *P*-values were obtained from a two-sided unpaired t-test. *N*, number of nuclei. **(H)** FACS plots showing U2OS cell-cycle distribution with or without 200 μM DRB in untreated and aphidicolin-treated conditions. **(I)** Average γH2AX signal per U2OS nucleus in different conditions. *P*-values were obtained from two-sided unpaired t-test. *N*, number of nuclei. **(J)** Aphidicolin-induced late-S/G2/M-seq values for late-RT break classes and RT-matched randomized controls. *P*-values were obtained from two-sided unpaired Wilcoxon test. *N*, number of breaks or randomized controls. **(K)** Representative genomic regions showing the local minimum (i.e., Termination Zones (TZs), red dots) for untreated pseudobulk RT. For all box plots in this figure, the center line indicates the median, the box limits indicate the 25th and 75th percentiles, and the whiskers extend to the minimum and maximum values excluding outliers (1.5 x interquartile range).

Supplementary Figure 7

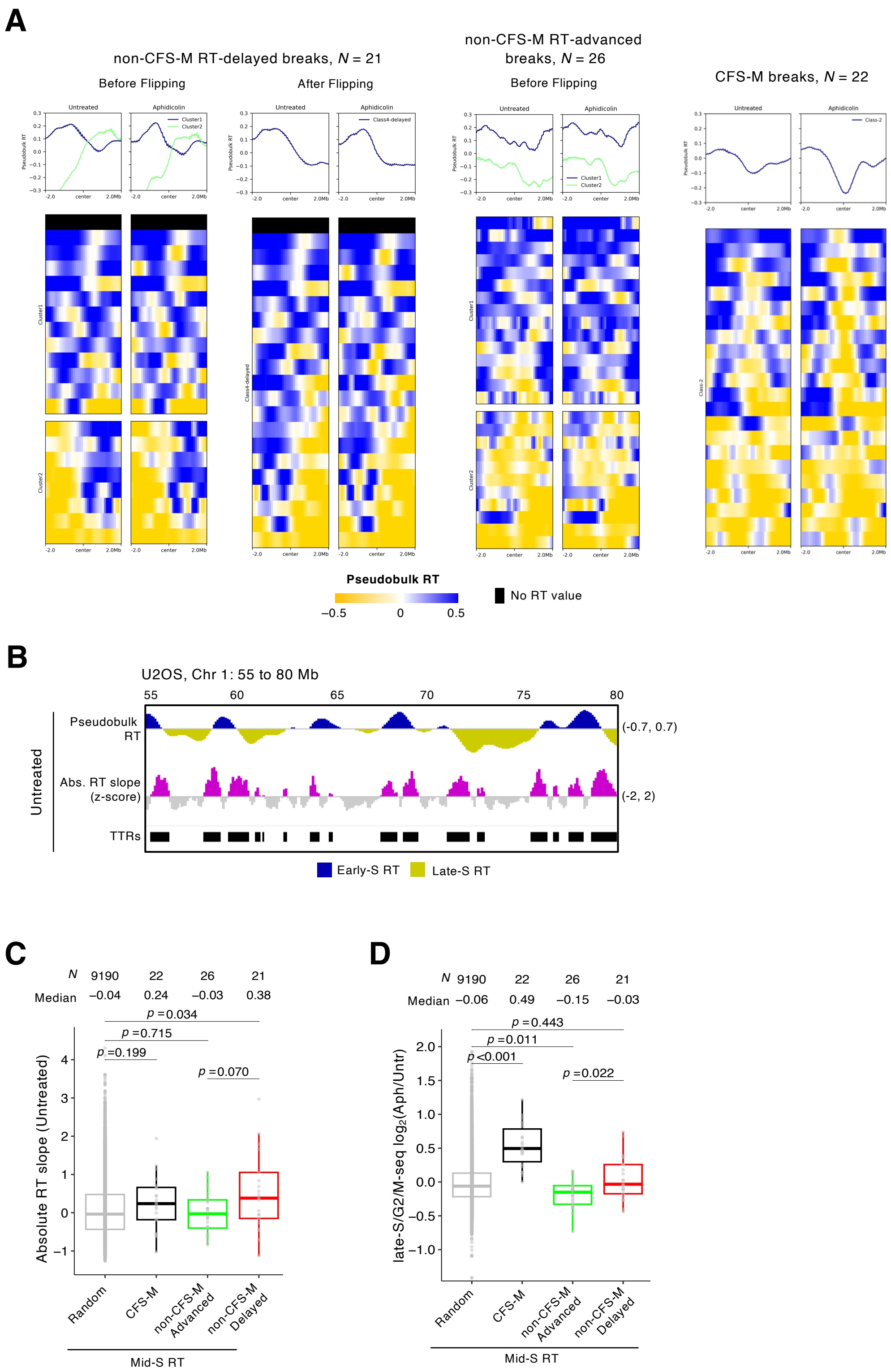

(Figure legend in next page)

Supplementary Figure. 7 | Characterization of mid-RT breaks

(A) Average line plot and heatmap showing the untreated and aphidicolin-treated pseudobulk RT values surrounding CFS-M, non-CFS-M RT-delayed and advanced breaks (+/– 2 Mb from the center of the break region). Non-CFS-M break regions were clustered using untreated RT with K-means clustering (K=2). Since the two clusters in non-CFS-M RT-delayed breaks transitioned in opposite directions, cluster-2 was flipped to facilitate averaging of pseudobulk-RT values. *N*, number of chromosome breaks. **(B)** Representative genomic region showing pseudobulk RT, z-score of absolute RT slope and identified TTRs (black bars) in untreated condition. Positive and negative slope values represent transitioning and flatter RT regions, respectively. **(C)** Boxplots with jitters showing U2OS's untreated absolute RT slope values for breaks in classes with mid-RT (CFS-M, black; non-CFS-M RT-delayed, red; non-CFS-M RT-advanced, green) and randomized control regions with similar RT (light gray). Positive and negative slope values represent transitioning and flatter RT regions, respectively. *P*-values were obtained from a two-sided unpaired Wilcoxon test. *N*, number of non-redundant breaks or randomized control regions. **(D)** Boxplots with jitters showing U2OS's aphidicolin-induced late-S/G2/M-seq values for breaks in classes with mid-RT (CFS-M, black; non-CFS-M RT-delayed, red; non-CFS-M RT-advanced, green) and randomized control regions with similar RT (light gray). *P*-values were obtained from a two-sided unpaired Wilcoxon test. *N*, number of non-redundant breaks or randomized control regions. For all box plots in this figure, the center line indicates the median, the box limits indicate the 25th and 75th percentiles, and the whiskers extend to the minimum and maximum values excluding outliers (1.5 x interquartile range).

Supplementary Figure 8

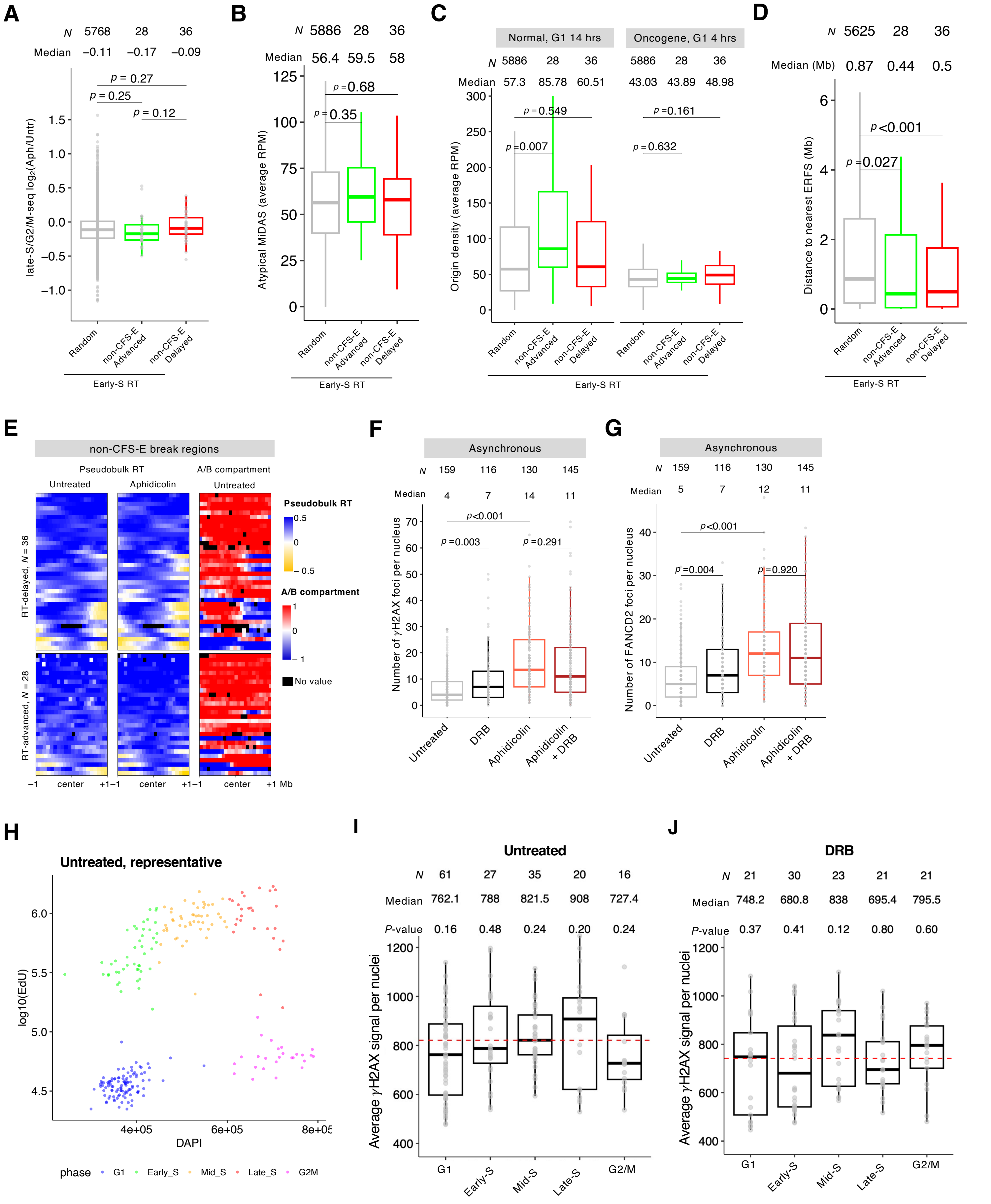

(Figure legend in next page)

Supplementary Figure. 8 | Characterization of early-RT breaks

(A-D) Boxplots showing U2OS's $\log_2$(aphidicolin/untreated) late-S/G2/M-seq values **(A)**, atypical-MiDAS signals **(B)**, origin density in normal and oncogene-induced condition **(C)**, and distance to nearest ERFS **(D)**, for non-CFS-E break regions (delayed, red; advanced, green) and randomized control regions with similar RT (light gray). *P*-values were obtained from a two-sided unpaired Wilcoxon test. *N*, number of non-redundant breaks or randomized control regions. **(E)** Heatmap showing pseudobulk RT with or without aphidicolin treatment, and untreated A/B compartment scores at and surrounding the non-CFS-E breaks (+/–1 Mb from the center of the break region). Non-CFS-E breaks were sub-categorized into RT-delayed and advanced upon aphidicolin treatment. *N*, number of chromosome break regions. **(F-G)** Boxplots with jitters showing number of γH2AX **(F)** and FANCD2 **(G)** foci per nuclei in asynchronous population with different conditions (untreated, grey; DRB, black; aphidicolin, red; aphidicolin+DRB, crimson). Two biological replicates were performed. *P*-values were obtained from a two-sided unpaired t-test. *N*, number of nuclei. **(H)** Scatter plot showing cell cycle phasing using DAPI and EdU signals of each nucleus from imaging data. Untreated condition is shown as representative. **(I–J)** Boxplots overlaid with jittered points showing the average γH2AX signal intensity per nucleus in untreated **(I)** and DRB-treated **(J)** conditions. Nuclei were assigned to cell-cycle phases based on DAPI and EdU imaging signals (see Supplementary Fig. 8H). *P*-values were calculated using the Wilcoxon test by comparing each cell-cycle phase with the asynchronous population mean. The red dotted horizontal line indicates the asynchronous mean γH2AX intensity. *N*, number of nuclei. For all box plots in this figure, the center line indicates the median, the box limits indicate the 25th and 75th percentiles, and the whiskers extend to the minimum and maximum values excluding outliers (1.5 x interquartile range).

Supplementary Figure 9

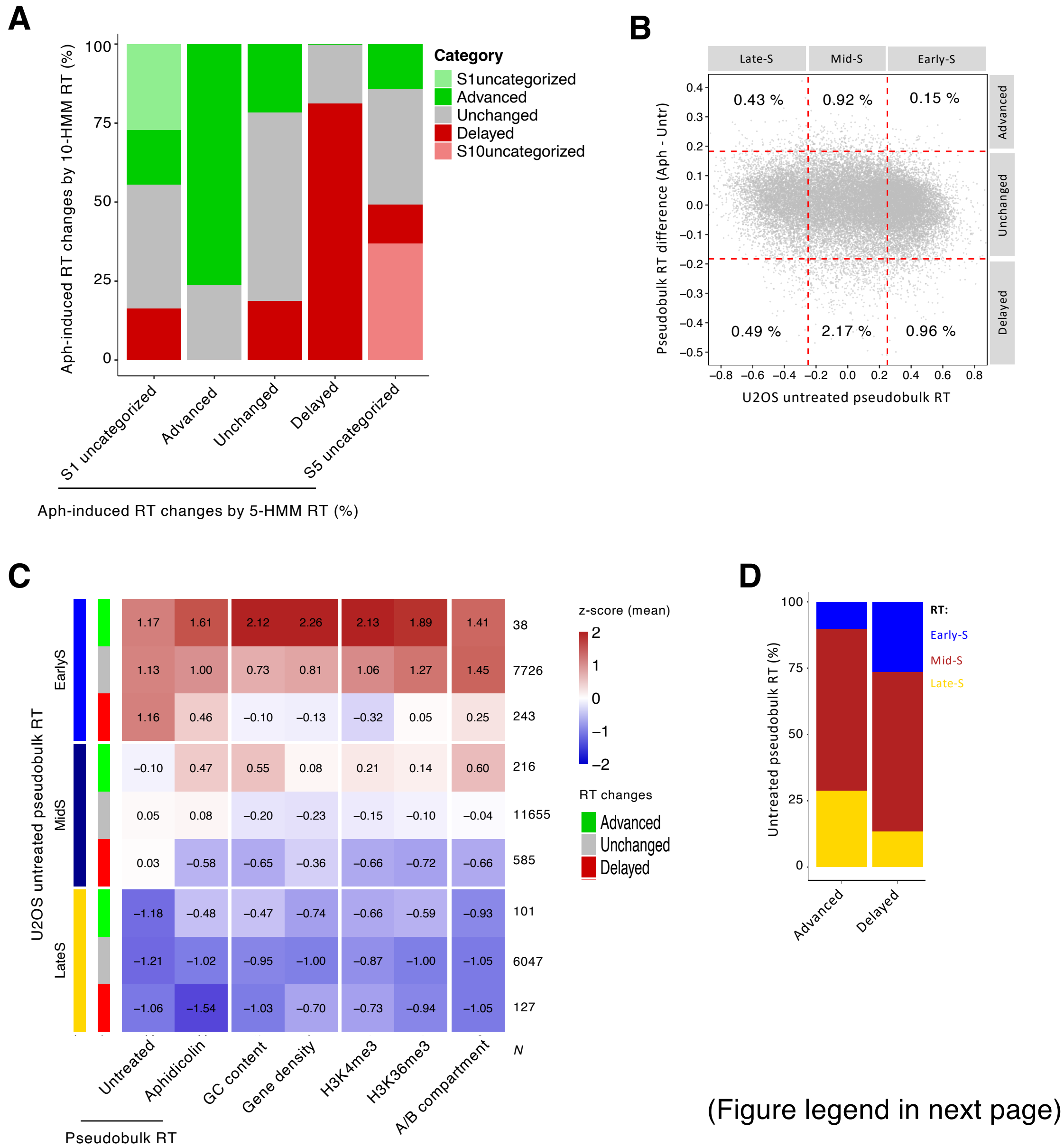

RT	RT changes	Untreated	Aphidicolin	GC content	Gene density	H3K4me3	H3K36me3	A/B compartment	N
EarlyS	Advanced	1.17	1.61	2.12	2.26	2.13	1.89	1.41	38
EarlyS	Unchanged	1.13	1.00	0.73	0.81	1.06	1.27	1.45	7726
EarlyS	Delayed	1.16	0.46	−0.10	−0.13	−0.32	0.05	0.25	243
MidS	Advanced	−0.10	0.47	0.55	0.08	0.21	0.14	0.60	216
MidS	Unchanged	0.05	0.08	−0.20	−0.23	−0.15	−0.10	−0.04	11655
MidS	Delayed	0.03	−0.58	−0.65	−0.36	−0.66	−0.72	−0.66	585
LateS	Advanced	−1.18	−0.48	−0.47	−0.74	−0.66	−0.59	−0.93	101
LateS	Unchanged	−1.21	−1.02	−0.95	−1.00	−0.87	−1.00	−1.05	6047
LateS	Delayed	−1.06	−1.54	−1.03	−0.70	−0.73	−0.94	−1.05	127

(Figure legend in next page)

Supplementary Figure. 9 | Global threshold to identify aphidicolin-induced RT changes

(A) Stacked bar chart comparing the categories of aphidicolin-induced RT changes between 5 and 10-HMM RT analysis. **(B)** Scatter plot showing untreated pseudobulk RT and RT difference (aphidicolin – untreated) for each 100-kb genomic bin. Vertical red dotted lines categorize the untreated pseudobulk RT into late, mid, and early S. Horizontal dotted red lines (2*SD) categorizes aphidicolin-induced RT differential into advanced, unchanged and delayed. Percentage of advanced and delayed across S-phase is shown in numbers. **(C)** Heatmap showing the average z-score values of U2OS pseudobulk RT, GC content, gene density, H3K4me3, H3K36me3 and A/B compartment score for aphidicolin-induced RT-change categories across early, mid and late-S phases identified using a global threshold (2*SD). *N*, number of 100-kb genomic bins. Statistics are provided in Supplementary Data 4. **(D)** Stacked bar chart showing the percentage of aphidicolin-induced RT advanced and delayed 100-kb bins across early/mid/late-S phases from Supplementary Fig. 9B.

Supplementary Figure 10

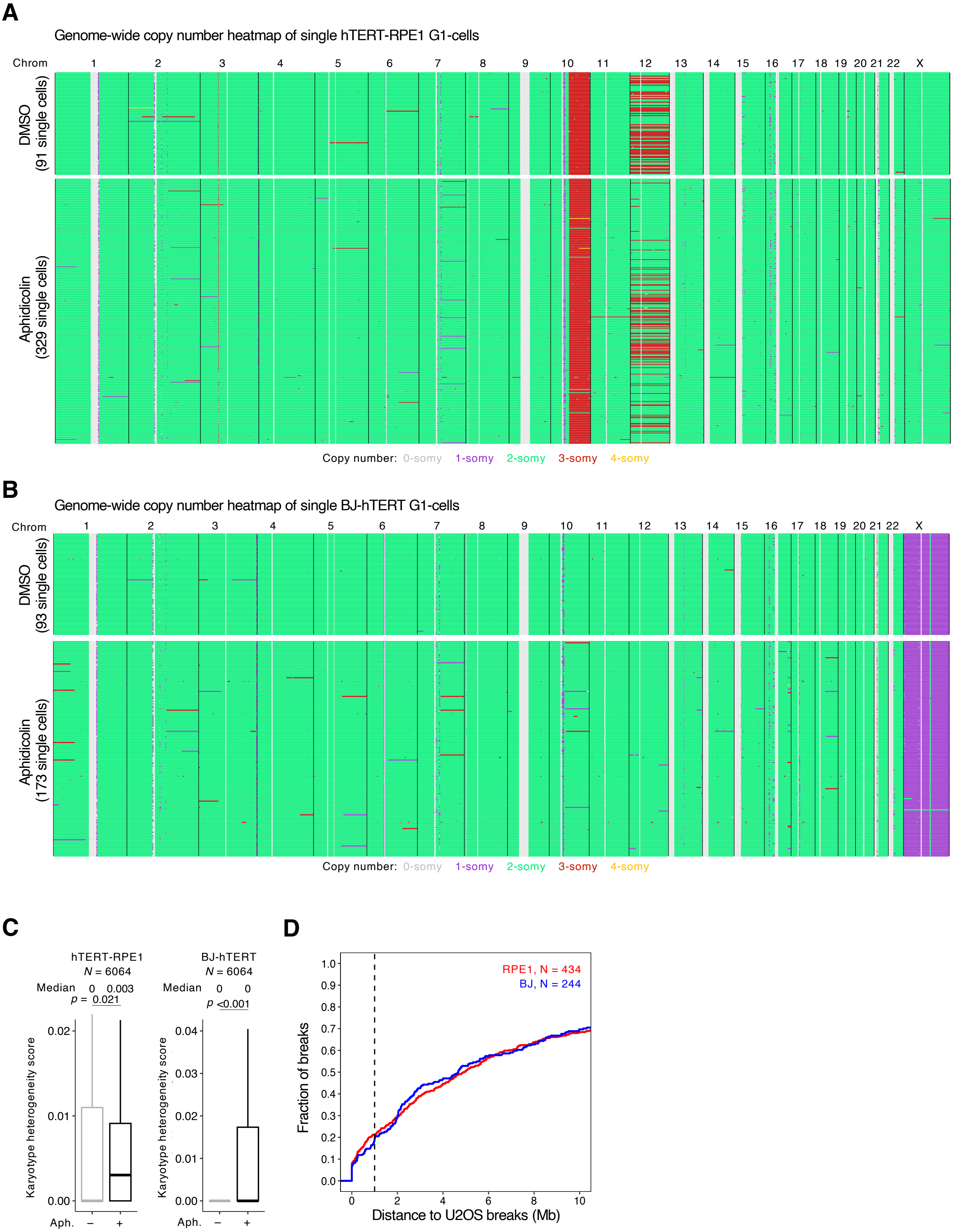

(Figure legend in next page)

Supplementary Figure. 10 | Chromosome copy number and break region analysis in other human cell lines

(A-B) Genome-wide copy number heatmap of single hTERT-RPE1 **(A)** and BJ-hTERT **(B)** G1 cells treated with or without low-dose aphidicolin. **(C)** Boxplots comparing karyotype heterogeneity score of RPE1 and BJ cells with or without aphidicolin treatment. *P*-values were obtained from a two-sided paired Wilcoxon test. *N*, number of 500-kb genomic bins. **(D)** Cumulative density plot showing the fraction of RPE1 and BJ break regions overlapped or close to U2OS break regions. *N*, number of aphidicolin-induced breaks. Black dotted line represents 1-Mb distance from overlap. For all box plots in this figure, the center line indicates the median, the box limits indicate the 25th and 75th percentiles, and the whiskers extend to the minimum and maximum values excluding outliers (1.5 x interquartile range).

Supplementary Figure 11

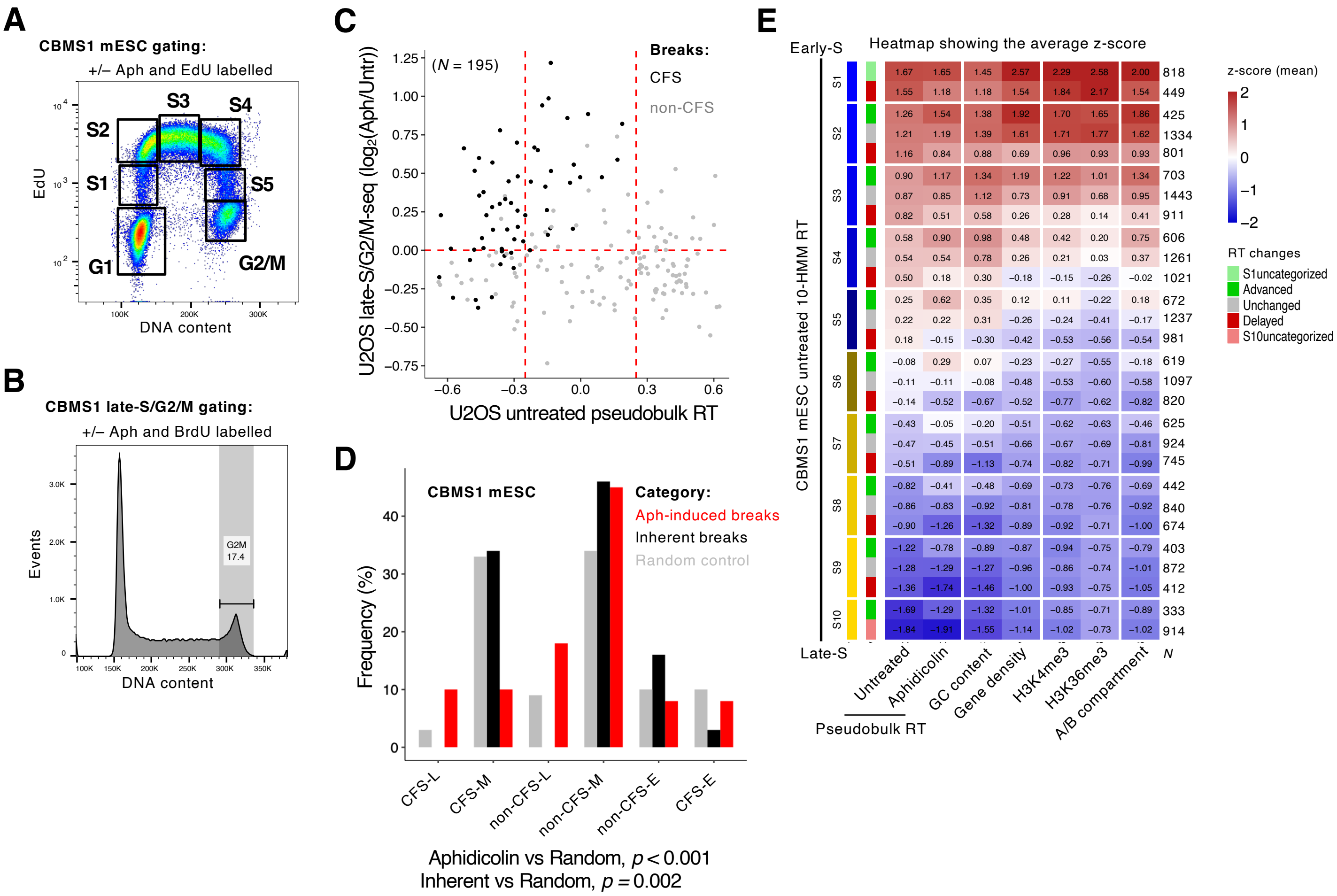

(Figure legend in next page)

Supplementary Figure. 11 | Chromosome copy number and break region analysis in mESCs

(A) Gating strategy used for sorting single G1 and S-phase CBMS1 mESCs. **(B)** Gating strategy used for sorting very late-S/G2/M fractions of CBMS1 mESCs. **(C)** Scatter plot showing untreated pseudobulk RT and aphidicolin-induced late-S/G2/M-seq (log_2(aphidicolin/untreated)) signals for each non-redundant U2OS break region. Each dot is a non-redundant break region (*N*) and they were colored based on the CFS (black) and non-CFS (gray), defined using MiDAS-seq. **(D)** A bar chart showing the frequency of breaks (red, aphidicolin; black, inherent) and 1,000 sets of randomized controls (grey) for each RT–lateS/G2/M categories in CBMS1 mESCs. *P*-values were obtained from the chi-square test. Pairwise comparison *P*-values are shown in Supplementary Data 4. **(E)** Heatmap showing the average z-score values of pseudobulk RT, GC content, gene density, H3K4me3, H3K36me3 and A/B compartment score for aphidicolin-induced RT-change categories in each untreated HMM fraction of CBMS1 mESC. 10-HMM RT fractions were ordered from S1 to S10, which represent early to late S-phase. *N*, number of 100-kb genomic bins. Statistics are provided in Supplementary Data 4.

Supplementary Figure 12

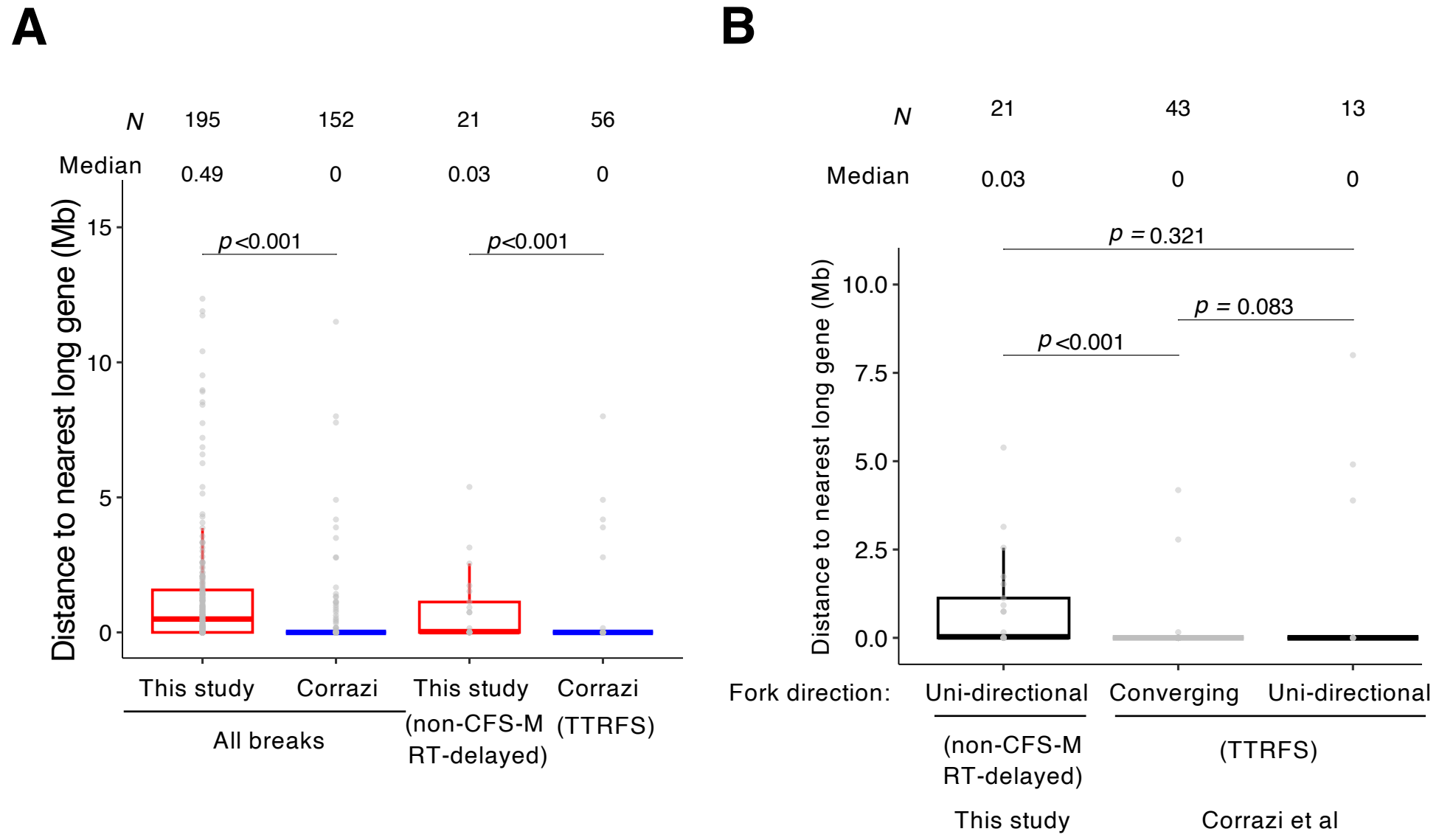

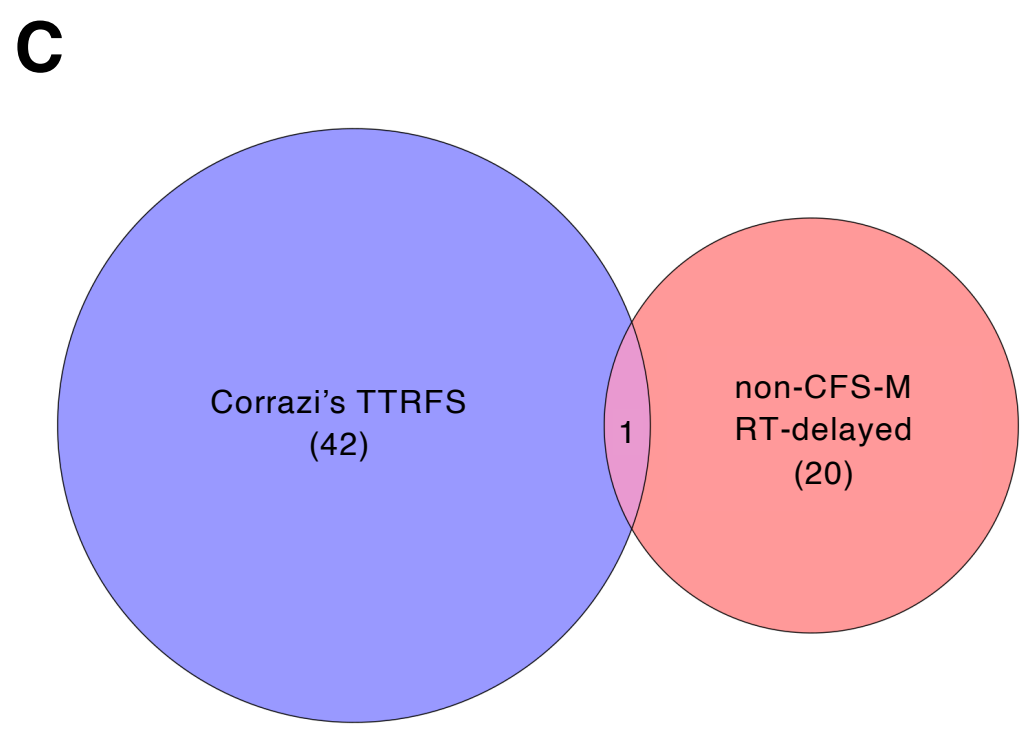

(Figure legend in next page)

Supplementary Figure. 12 | Comparison between TTRFS and non-CFS-M RT-delayed breaks

(A) Boxplots with jitters showing the distance to the nearest long gene (>= 300 kb) from U2OS break regions (all, and non-CFS-M RT-delayed) in our study (red) compared with mNPC translocation breakpoints (all and TTRFS) in Corazzi et al, *Nat. Comm*. 2024 (blue)[43]. *P*-values were obtained from a two-sided unpaired Wilcoxon test. *N*, number of breaks. **(B)** Boxplots with jitters showing the distance to the nearest long gene (>= 300 kb) from non-CFS-M RT-delayed and TTRFS breaks subdivided into those with converging and uni-directional forks (converging - grey, uni-directional - black). *P*-values were obtained from a two-sided unpaired Wilcoxon test. *N*, number of breaks. **(C)** Venn diagram showing overlap between TTRFS ($N = 43$, blue) and non-CFS-M RT-delayed breaks ($N = 21$, red). For all box plots in this figure, the center line indicates the median, the box limits indicate the 25th and 75th percentiles, and the whiskers extend to the minimum and maximum values excluding outliers (1.5 x interquartile range).

Supplementary Notes

Supplementary Note 1 | On the resolution of karyotype analysis

For reliable karyotype analysis of single G1-phase cells, we empirically reasoned that: (1) the number of peaks in the uniquely mapped read-density profile should reflect the known karyotype of the cell line, and (2) the average read count of 1-somy bins should exceed 20–30 in order to be distinguished from noise.

Our sequencing depth (~ 4–6 million reads) typically yielded ~0.1× genome coverage. For CBMS1 mESCs, whose karyotype is known to be near-diploid except for the frequent loss of one copy of the X chromosome, we assessed the effective resolution by performing copy-number analysis on 100 untreated G1-phase CBMS1 mESCs. We found that the expected bimodal distribution became visually apparent at 500-kb resolution, where the smaller peak on the left corresponded to 1-somy and the larger peak on the right corresponded to 2-somy, as identified by AneuFinder. The average read count for 1-somy bins at this resolution was 197 (Supplementary Fig. 1C).

Similarly, in 100 untreated G1-phase U2OS cells with comparable coverage, peaks corresponding to different copy numbers also became visually distinguishable at 500-kb bin resolution (Supplementary Fig. 1D). Therefore, at ~0.1× coverage, we concluded that 500-kb bin size is the minimum resolution that satisfies the above two criteria and is suitable for copy-number analysis.

Supplementary Note 2 | Overlap analysis between breaks and CFSs

We found that 16% of MiDAS-seq CFSs overlapped with our aphidicolin-induced breaks, which was relatively low. Because inherent breaks (defined as those shared between untreated and aphidicolin-treated conditions) were excluded, this could have possibly removed a subset of CFS-class breaks. Consistent with our hypothesis, we found that 24% (76/324) of MiDAS-seq CFSs overlapped with inherent breaks. Therefore, overall, we found that 40% (129/324) of MiDAS-seq CFSs overlapped with our breaks in aphidicolin-treated cells. It is also important to note that our break analysis is based on a limited number of single cells treated with aphidicolin (N = 43), which may have underestimated the true overlap with MiDAS-seq CFSs.

On the other hand, we found that 45% of cytogenetically defined CFSs in human fibroblast cells overlapped with our aphidicolin-induced breaks, which was relatively high, especially for a cross-cell-type comparison. The low resolution of cytogenetically defined CFSs (average size = 11.5 Mb) could have increased the likelihood of overlap. In addition, although CFSs have often been described as cell-type-specific, many of these conclusions are based on cytogenetic analyses of a limited number of well-characterized CFS loci. Moreover, recent sequencing-based studies suggest that a substantial fraction of CFSs is conserved across cell types[8,9]. These findings indicate that both conserved and cell-type-specific fragile regions exist. Therefore, the relatively high overlap of CFSs across cell types may reflect a combination of resolution differences between cytogenetics and sequencing-based techniques, as well as the presence of conserved CFSs.

Supplementary Note 3 | Log$_2$repliscore RT analysis

In the conventional scRepli-seq analytical pipeline, log$_2$[(mappability-corrected S-phase reads)/median] (log$_2$median) values are used to generate single-cell RT profiles of mid-S cells[12,17,56]. However, we realized that using the genome-wide median of counted reads per bin to calculate RT (log$_2$median RT) was challenging for cells in the earliest or latest S-phase fractions compared to binarized RT (Supplementary Fig. 2B). Therefore, we decided to calculate the percentage replication score (Repliscore; percentage of the genome replicated) of each 10-cell S-phase fraction from binarized RT and the proportion of the complement of repliscore to 100 is used in the place of median value (see Methods and Supplementary Fig. 2A,B). We used repliscore as it showed a very good correlation with the DNA content of each fraction obtained from FACS plot (Supplementary Fig. 2C). We call this 'log$_2$repliscore RT'.

In addition, one can use DNA content (meta-data from FACS) as an alternative to repliscore to obtain log$_2$DNAcontent RT, which gave similar results as log2repliscore RT (Supplementary Fig. 2B). Although we did not use this method in this work (due to the unavailability of FACS metadata for public datasets, discussed later (Fig. 7B,D) in this manuscript), the process of log$_2$DNAcontent RT calculation is simpler and faster, as it saves the computation time associated with the generation of binarized RT.

Supplementary Note 4 | Comparison between MiDAS-seq and late-S/G2/M-seq

MiDAS-seq: Cells were first arrested at the G1/S boundary with thymidine, released into S-phase with or without low-dose aphidicolin, and then arrested again at the G2/M boundary using RO3306 (a CDK1 inhibitor). Cells at the G2/M boundary were subsequently released into fresh medium containing EdU to label DNA synthesis occurring in early mitosis, in the presence of hydroxyurea (to block any residual S-phase replication) and nocodazole (to arrest cells in prometaphase). Mitotic cells were then collected by mitotic shake-off, and EdU-labeled DNA was pulled down and sequenced. The advantage of MiDAS-seq is its high signal-to-noise ratio, making it highly sensitive in detecting DNA synthesis in early mitosis. However, the method involves multiple drug treatments, including prolonged exposure to RO3306, which may perturb cellular physiology and reduce MiDAS signals if treated longer. Another limitation is that MiDAS-seq cannot detect DNA replication occurring in the G2 phase.

late-S/G2/M-seq: Cells treated with or without low-dose aphidicolin were pulse-labeled with BrdU for 30 minutes, and the very-late-S/G2/M fraction (i.e., 4C DNA content population) was collected from a simple DNA-content cell-cycle FACS plot. After fragmentation of genomic DNA, BrdU-labeled DNA was immunoprecipitated and sequenced. The signal-to-noise ratio is somewhat low, as there is a possibility of contamination by very late S-phase cells during cell sorting. However, this experiment is much simpler with fewer drug treatments compared to MiDAS-seq and yet gives comparable results.

In addition, we observed stronger late-S/G2/M-seq signals for CFS-M breaks compared to CFS-L (Fig. 2D), whereas MiDAS-seq signals was comparable for both (Supplementary Fig. 6B). We speculate that this is due to a higher BrdU-IP signal in the untreated late-S/G2/M-seq for CFS-L (late-S RT) compared to CFS-M (mid-S RT), likely due to contaminating late S-phase cells. Because overestimated untreated late-S/G2/M-seq signal values were used as the denominator for CFS-L in Fig. 2D, the CFS-L late-S/G2/M-seq log2(aphidicolin/untreated) values were probably an underestimate.

Supplementary Note 5 | Effect of aphidicolin on non-CFS break classes

Because non-CFS break regions are ‘rare’ events (Fig. 1H), their absence in untreated cells

cannot necessarily be used to formally distinguish a truly aphidicolin-specific break event from a low-frequency event that escaped detection in untreated cells due to finite sampling. Therefore, it is difficult to determine statistically whether a given non-CFS break region is exclusively induced by aphidicolin.

However, aphidicolin-dependent fragility should nevertheless be reflected by an increase in break frequency following aphidicolin treatment. We therefore quantified break frequencies at all break regions identified in aphidicolin-treated cells, which are expected to include both break regions newly generated by aphidicolin treatment and pre-existing constitutive fragile regions with a range of baseline break frequencies. Specifically, break regions were categorized according to their positions on the RT-MiDAS axes, and for each region, we calculated the percentage of single cells carrying a chromosome break within that region in untreated and aphidicolin-treated conditions.

As a result, all five break classes exhibited increased break frequencies following aphidicolin treatment in three human cell lines (Supplementary Fig. 4A–D; U2OS, RPE1, and BJ cells). Mouse ESCs showed a similar trend but did not reach statistical significance because of the limited sample size. Taken together, these findings indicate that aphidicolin-induced replication stress enhances instability not only at canonical CFSs but also across all non-CFS classes. This phenomenon is unlikely to be explained solely by the limited detection sensitivity of these breaks in untreated samples due to finite sampling. Rather, the results are consistent with the interpretation that replication stress enhances instability at both CFS and non-CFS regions.

For these reasons, we use the term “aphidicolin-induced breaks” throughout this study to denote break regions whose frequencies are enhanced following aphidicolin treatment.

Supplementary Note 6 | Limitations of 10-cell Repli-seq’s Pseudobulk RT

We noticed that the amount of aphidicolin-induced RT-advance (~ one third) in CFS-L was higher compared to CFS-M break regions (Fig. 2C). We speculate that this could be a limitation of 10-cell Repli-seq. While 10-cell Repli-seq pseudobulk RT is much better than two-fraction BrdU-IP Repli-seq (Supplementary Fig. 3A–E), it is possible that it may have difficulties in

identifying RT-delay in the very late-S phase, though not to the same extent as the two-fraction BrdU-IP Repli-seq.

Supplementary Note 7 | Linear relationship between gene size and TAD boundary overlap

TADs are typically ~200 kb to 1 Mb in size[57], and the presence of actively transcribed long genes at TAD boundaries has been proposed as a feature of CFSs[20]. However, we reasoned that this observation may be largely expected, as longer genes have a higher probability of overlapping with TAD boundaries simply due to their size. Indeed, we observed a positive correlation between gene length and the number of TAD boundaries overlapped, indicating an approximately linear relationship (Supplementary Fig. 6E).

Furthermore, for each locus reported in Fig. 4n of Sarni et al.[20], we found that genes categorized as inter-TAD (i.e., overlapping TAD boundaries) were significantly larger than those categorized as intra-TAD (Supplementary Fig. 6F). This suggests that the comparison presented in their study may not be sufficient to establish TAD boundaries as an intrinsic feature of CFSs. Additionally, long genes generally replicate during mid- to late-S phase, underscoring the importance of using randomized controls with similar RT. Using RT-matched random controls for CFS-L and CFS-M breaks, we found that CFS breaks are not preferentially located near TAD boundaries (Supplementary Fig. 6D), consistent with a recent report[9].

Supplementary Note 8 | Limitations of in-silico identification of TZs

Our computational analysis identified 1,771 TZs across the genome in untreated U2OS cells (see Methods), accounting for approximately 0.06% of the genome. Briefly, TZs were defined as local minima along the RT profile flanked by at least three consecutive descending bins on each side, forming a descending ladder pattern toward the minimum. In addition, we further limited the analysis to TZs that replicated in late S-phase. These stringent criteria may have limited the number of detected TZs.

Supplementary Note 9 | Potential effects of altered cell-cycle distribution on γH2AX enrichment

We observed increased γH2AX signals at representative non-CFS break foci following

aphidicolin (Fig. 4B, 5C) and aphidicolin+DRB (Fig. 3D) treatments. Because aphidicolin treatment substantially alters cell-cycle distribution, resulting in an increased proportion of cells in S and G2/M phases (Supplementary Fig. 6H), altered cell-cycle composition could potentially influence the interpretation of γH2AX enrichment at these loci.

To address this possibility, we categorized nuclei into different cell-cycle phases based on their DAPI and EdU imaging signals and examined whether S- or G2/M-phase cells in untreated or DRB-treated conditions exhibited elevated γH2AX signals relative to the asynchronous population mean (Fig. 5D and Supplementary Fig. 8H). Interestingly, we did not observe a substantial increase in γH2AX signals in S- or G2/M-phase cells under either untreated or DRB-treated conditions (Supplementary Fig. 8I,J).

These observations suggest that enrichment of S- or G2/M-phase cells alone is unlikely to fully explain the increased γH2AX signals observed at representative non-CFS loci following aphidicolin or aphidicolin+DRB treatment.

Supplementary Note 10 | Global threshold to define aphidicolin-induced RT changes

As discussed in the main text, using 10-HMM RT analysis, we identified RT changes in approximately 50% of the genome upon aphidicolin treatment (Fig. 6E) and showed that these changes are not stochastic variation of RT (Supplementary Fig. 9A). On the other hand, applying a global threshold of two standard deviations identified RT changes in ~5% of the genome upon aphidicolin treatment (Supplementary Fig. 9B), consistent with a recent report[20]. Interestingly, similar to 10-HMM RT analysis, aphidicolin-induced RT advanced and delayed regions across early/mid/late-S fractions deviated from their mean genomic and epigenomic features in untreated conditions (Supplementary Fig. 9C; Supplementary Data 4). These results are consistent with our idea that aphidicolin-induced RT changes can be distinguished from genomic/epigenomic features in untreated cells.

Despite this concordance, we favor the 10-HMM RT analysis for identifying aphidicolin-induced RT changes, because applying a global threshold underestimates smaller RT shifts in early- and late-S fractions, which spend less time in S phase. Consequently, mid-S regions show larger RT

delays than late-S regions and larger RT advances than early-S regions, reflecting their longer residence time in S phase (Supplementary Fig. 9B,D).

Supplementary References

56. Takahashi, S. et al. Embryonic genome instability upon DNA replication timing program emergence. *Nature* **633**, 686–694 (2024).
57. Nora, E. P. et al. Spatial partitioning of the regulatory landscape of the X-inactivation centre. *Nature* **485**, 381–385 (2012).
