# Supplementary material for: Single-cell mapping of chromosome breaks identifies multiple fragile site classes with distinct DNA replication timing landscapes
Source: Nat Commun. 2026 Sep 23;17:9055. doi: 10.1038/s41467-026-76451-1 (PMC13601574; doi:10.1038/s41467-026-76451-1)
Supplement: Supplementary file 2 [file 41467_2026_76451_MOESM2_ESM.pdf]

Description of Additional Supplementary Files

File Name: Supplementary Data 1
Description: Aphidicolin-induced break regions list for U2OS, hTERT-RPE1, BJ-hTERT and CBMS1 mESCs

File Name: Supplementary Data 2
Description: Processed files for U2OS, hTERT-RPE1, BJ-hTERT and CBMS1 mESCs

File Name: Supplementary Data 3
Description: Data generated in this study, accession codes of public datasets and list of BACs used in this study.

File Name: Supplementary Data 4
Description: Statistical tests and *P*-values associated with the figures.
