# Supplementary material for: Single-cell mapping of chromosome breaks identifies multiple fragile site classes with distinct DNA replication timing landscapes
Source: Nat Commun. 2026 Sep 23;17:9055. doi: 10.1038/s41467-026-76451-1 (PMC13601574; doi:10.1038/s41467-026-76451-1)
Supplement: Supplementary file 3 [file 41467_2026_76451_MOESM7_ESM.pdf]

nature portfolio

Corresponding author(s): Ichiro Hiratani

Last updated by author(s): July 10, 2026

Reporting Summary

Nature Portfolio wishes to improve the reproducibility of the work that we publish. This form provides structure for consistency and transparency in reporting. For further information on Nature Portfolio policies, see our Editorial Policies and the Editorial Policy Checklist.

Statistics

For all statistical analyses, confirm that the following items are present in the figure legend, table legend, main text, or Methods section.

n/a	Confirmed	
☐	☒	The exact sample size (*n*) for each experimental group/condition, given as a discrete number and unit of measurement
☐	☒	A statement on whether measurements were taken from distinct samples or whether the same sample was measured repeatedly
☐	☒	The statistical test(s) used AND whether they are one- or two-sided<br>*Only common tests should be described solely by name; describe more complex techniques in the Methods section.*
☒	☐	A description of all covariates tested
☐	☒	A description of any assumptions or corrections, such as tests of normality and adjustment for multiple comparisons
☐	☒	A full description of the statistical parameters including central tendency (e.g. means) or other basic estimates (e.g. regression coefficient) AND variation (e.g. standard deviation) or associated estimates of uncertainty (e.g. confidence intervals)
☐	☒	For null hypothesis testing, the test statistic (e.g. *F*, *t*, *r*) with confidence intervals, effect sizes, degrees of freedom and *P* value noted<br>*Give P values as exact values whenever suitable.*
☒	☐	For Bayesian analysis, information on the choice of priors and Markov chain Monte Carlo settings
☒	☐	For hierarchical and complex designs, identification of the appropriate level for tests and full reporting of outcomes
☐	☒	Estimates of effect sizes (e.g. Cohen's *d*, Pearson's *r*), indicating how they were calculated

Our web collection on statistics for biologists contains articles on many of the points above.

Software and code

Policy information about availability of computer code

Data collection	Cell sorter software (Sony), MultiNA (Shimadzu), Tapestation 4150 (Agilent), and DeltaVision Olympus IX71

Data analysis	The following softwares were used in this paper and cited in the online methods section.<br>FlowJo v10.10.0 to analyze FACS cell cycle profiles.<br>fastq-dump v2.8.2 to download publicly available fastq files.<br>FastQC v0.11.5 to check quality of sequenced reads.<br>Cutadapt v1.15 to trim adapters.<br>BWA v0.7.17-r1188 (bwa-aln) to map sequenced reads.<br>SAMtools v1.7, Picard-tools v2.8.1, and BEDtools v2.26.0 to process mapped reads.<br>Samstat v1.5.1 to check quality of mapped reads.<br>HiC-Pro v3.1.0 to analyze Hi-C data (until generation of contact matrices).<br>Cooltools v0.5.4 to obtain A/B compartment profiles.<br>ViennaRNA v2.6.4 to calculate DNA free energy.<br>LiftOver for lifting between different genomes.<br>deepTools v3.5.5 to create heatmaps and average plots.<br>AneuFinder (R package, v1.2.1 for replication timing analysis and v1.22.0 for chromosome break region analysis).<br>RHmm (R package, v2.2.1 for 10-HMM RT analysis).<br>rstatix (R package, v0.7.2 for statistical analysis).<br>ggplot2 (R package, v3.5.1), ggpubr (R package, v0.6.0), reshape2 (R package, v1.4.4), and pheatmap (R package, v1.0.12) for plotting.<br>Custom codes used in this study are available on GitHub (https://github.com/jothivanan-elumalai/single_cell_genome_fragility) and have also been deposited in Zenodo (https://doi.org/10.5281/zenodo.21292001)

For manuscripts utilizing custom algorithms or software that are central to the research but not yet described in published literature, software must be made available to editors and reviewers. We strongly encourage code deposition in a community repository (e.g. GitHub). See the Nature Portfolio guidelines for submitting code & software for further information.

Data

Policy information about availability of data

All manuscripts must include a data availability statement. This statement should provide the following information, where applicable:

- Accession codes, unique identifiers, or web links for publicly available datasets
- A description of any restrictions on data availability
- For clinical datasets or third party data, please ensure that the statement adheres to our policy

The scRepli-seq and BrdU-IP-seq data generated in this study have been deposited in the Gene Expression Omnibus (GEO) database under accession code GSE310972. Processed data files and accession codes of previously published data are provided in Supplementary Data 2 and 3, respectively. Source data are provided with this paper.

Research involving human participants, their data, or biological material

Policy information about studies with human participants or human data. See also policy information about sex, gender (identity/presentation), and sexual orientation and race, ethnicity and racism.

Reporting on sex and gender	Not applicable
Reporting on race, ethnicity, or other socially relevant groupings	Not applicable
Population characteristics	Not applicable
Recruitment	Not applicable
Ethics oversight	Not applicable

Note that full information on the approval of the study protocol must also be provided in the manuscript.

Field-specific reporting

Please select the one below that is the best fit for your research. If you are not sure, read the appropriate sections before making your selection.

☒ Life sciences ☐ Behavioural & social sciences ☐ Ecological, evolutionary & environmental sciences

For a reference copy of the document with all sections, see nature.com/documents/nr-reporting-summary-flat.pdf

Life sciences study design

All studies must disclose on these points even when the disclosure is negative.

Sample size	Sample sizes were chosen based on empirical knowledge and common practice in the field. Technical feasibility, sequencing depth requirements, and the number of cells or independent samples sufficient to detect reproducible effects were also considered.

Data exclusions	In single-cell CNV analysis, we performed multivariate clustering for quality control, and clusters with spikiness values greater than 1 were excluded, as they denote failed libraries.
Replication	Experimental findings were verified by independent biological replicates where applicable, including repeated imaging and sequencing experiments. All attempts at replication were successful.
Randomization	Randomized control genomic regions were generated and described in detail in Methods section.
Blinding	Blinding was not relevant to this study because all metrics were derived from absolute quantitative methods without human subjectivity.

Reporting for specific materials, systems and methods

We require information from authors about some types of materials, experimental systems and methods used in many studies. Here, indicate whether each material, system or method listed is relevant to your study. If you are not sure if a list item applies to your research, read the appropriate section before selecting a response.

Materials & experimental systems

n/a	Involved in the study	
☐	☒	Antibodies
☐	☒	Eukaryotic cell lines
☒	☐	Palaeontology and archaeology
☒	☐	Animals and other organisms
☒	☐	Clinical data
☒	☐	Dual use research of concern
☒	☐	Plants

Methods

n/a	Involved in the study	
☒	☐	ChIP-seq
☐	☒	Flow cytometry
☒	☐	MRI-based neuroimaging

Antibodies

Antibodies used	Anti-BrdU antibody (dilution to 12.5 ug/ml by PBS) from BD Biosciences Pharmingen, 555627<br>rabbit anti-FANCD2, Abcam, ab108928<br>mouse anti-gH2AX, 20A8, gift from Dr. Hiroshi Kimura<br>rabbit anti-gH2AX, Abcam, ab81299
Validation	Anti-BrdU antibody was used to immunoprecipitate BrdU-containing DNA (Takahashi et al., Nature Genetics 2019).<br>mouse anti-gH2AX antibody was used in Yamagata et al., Scientific Reports 2019

Eukaryotic cell lines

Policy information about cell lines and Sex and Gender in Research

Cell line source(s)	CBMS1 mESCs (Dr. Kimi Araki's lab, Kumamoto University)<br>Human U2OS (Dr. Minoru Takata, Kyoto University)
Authentication	Karyotype (CBMS1 mESCs and U2OS) was verified using an NGS technology, scRepli-seq, using single and 100-G1 cells.
Mycoplasma contamination	The cell lines were not tested for mycoplasma contamination.
Commonly misidentified lines (See ICLAC register)	No commonly misidentified cell lines were used.

Plants

Seed stocks	Not applicable
Novel plant genotypes	Not applicable
Authentication	Not applicable

Flow Cytometry

Plots

Confirm that:

☒ The axis labels state the marker and fluorochrome used (e.g. CD4-FITC).

☒ The axis scales are clearly visible. Include numbers along axes only for bottom left plot of group (a 'group' is an analysis of identical markers).

☒ All plots are contour plots with outliers or pseudocolor plots.

☒ A numerical value for number of cells or percentage (with statistics) is provided.

Methodology

Sample preparation	Cells were fixed with 75% ethanol, and then stained with AF488 picolyl azide and/or Fxcycle Violet to asses replicating population and/or DNA content, respectively.
Instrument	SH800 or MA900 cell sorter (SONY)
Software	The data was collected by Cell Sorter Software and analyzed using FlowJo
Cell population abundance	Approximately 1,000,000-2,000,000 cells were used for cell sorting per sample. Around 90% of the cells showed the typical cell cycle profile.
Gating strategy	FSC/BSC gate was used for gating the population of cells to exclude cell debris. Then, FSC-A/H gate was used for excluding cell doublets. Single-G1/S or 10-cell S-phase fractions were sorted from 2D cell-cycle profile.

☒ Tick this box to confirm that a figure exemplifying the gating strategy is provided in the Supplementary Information.
