# Supplementary material for: Single-cell mapping of chromosome breaks identifies multiple fragile site classes with distinct DNA replication timing landscapes
Source: Nat Commun. 2026 Sep 23;17:9055. doi: 10.1038/s41467-026-76451-1 (PMC13601574; doi:10.1038/s41467-026-76451-1)
Supplement: Supplementary file 4 [file 41467_2026_76451_MOESM8_ESM.pdf]

nature portfolio

# Peer Review File

# Single-cell mapping of chromosome breaks identifies multiple fragile site classes with distinct DNA replication timing landscapes

Corresponding Author: Dr Ichiro Hiratani

**This file contains all reviewer reports in order by version, followed by all author rebuttals in order by version.**

Version 0:

Reviewer comments:

Reviewer #1

(Remarks to the Author)
This manuscript maps aphidicolin-induced fragile sites in single mammalian cells. Previous studies have mapped fragile sites induced by aphidicolin in human and mouse cells, as well as fragile sites induced by driving entry of cells into S phase or in cells that are deficient for homologous recombination. The previous studies have studied populations of cells, meaning that rare fragile sites could not be identified. One exception is the study by Shaikh et al (Genome Biology 2022 = ref. 12 of this manuscript), which examined fragile sites in single cells using the same method as the one employed in the current manuscript. Shaikh et al studied non-transformed cells (RPE1 and BJ) and identified fragile sites, which were mostly associated with transcribed large genes.

In the current manuscript the authors focus on U2OS cells, but also study RPE1 and mouse ESCs. The analysis is more detailed than that performed by Shaikh et al and reveals, according to the authors, 5 types of fragile sites.

Types 1 and 2 are classical common fragile sites (CFSs) mapping to mid-S and late-S replicating regions. Mechanistically they are very similar. They could be even considered as one type.

Types 3 and 4 are not well-defined mechanistically. They map to mid-S and late-S replicating regions. They were not recurrent (unlike types 1 and 2), but it remains possible that fragility at these sites is due to the same mechanism as that leading to fragility at sites of types 1 and 2. This is perhaps the part of the manuscript that deserves greatest attention by the authors. To define too many classes of sites, when these are not very well defined is not helpful.

Type 5 sites are sites of transcription-replication conflicts. They tend to map to early-S replicating regions. I wonder if they overlap with the oncogene-induced regions defined by Macheret et al (Nature 2018).

Overall, I am not sure that the current study provides novel mechanistic insights on how fragile sites are generated in response to aphidicolin, but the study is carefully performed and the data will be useful to the community. Thus, I would recommend publication after some analysis reveals whether types 3 and 4 are distinct from types 1, 2 or 5. Regarding the publication venue, I would not make a strong case for Nature Communications versus a Genomics journal.

Reviewer #2

(Remarks to the Author)
In this work, Elumalai and Hiratani performed a study to map and classify aphidicolin-induced chromosome breaks identified by single-cell Whole-Genome Sequencing (scWGS) in human (U2OS cells) and mouse (CBMS1 mESC) cell lines. Based on the replication timing (RT) data (early, mid, or late) and previously published aphidicolin-induced MiDAS-seq signals (or G2/M-seq data generated in the current study) that enrich within common fragile sites (CFSs), they classified chromosomal break regions into five classes. Two classes (Class-1 and Class-2), with recurrent breaks and mid or late RT, are associated with CFSs, and three other classes are not enriched with MiDAS-seq signals. The authors further perform additional

bioinformatics analyses to try to identify the genomic and epigenomic features (including 3D genome organization) of different classes of breaks. They concluded that Class-4 is a novel class of breaks that coincided with RT transition regions (TTRs) and A/B compartment boundaries, and Class-5 is another class that showed early RT and enrichment for R-loops and G quadruplexes that might be associated with transcription-replication conflicts. In addition, the authors re-analyzed the previously published data of aphidicolin-induced chromosome breaks mapped by scWGS in RPE1 epithelial cells and BJ fibroblast cells to show that these 5 classes of breaks also exist in other human cell lines.

While the topic aligns with an important area in the field of genome instability, the current manuscript falls short of the journal's standards for novelty, rigor, and mechanistic insights. Several of the findings described are either overlapping with or lack sufficient differentiation from previous seminal works, such as Sarni et al. (Nat. Commun., 2020) and Shaikh et al. (Genome Biol., 2022). Moreover, key conclusions, such as the identification of novel classes of breaks (e.g., Class-4 and Class-5), appear inadequately supported by the presented analyses (see below for detailed comments). The study also misses opportunities to leverage its dataset to address critical mechanistic questions in the field. Therefore, the current study is largely premature, and substantial revisions are required before this manuscript can be considered for publication.

Major comments:

1. Novelty and Contextualization

The findings presented here overlap extensively with prior publications, particularly Sarni et al. (Nat. Commun., 2020) and Shaikh et al. (Genome Biol., 2022). For example:

• 1a. The classification of chromosome breaks based on RT and genomic features is not new. Sarni et al. already established such classifications, which align closely with the classes described here.
In study by Sarni et al., they classified aphidicolin-induced RT-variable regions into 5 different classes (called “cluster” or “signature”) based on RT data (early, mid, or late) and the stage of alternation (i.e. advanced or delayed RT in aphidicolin-treated cells). The Class-1 and 2 identified in the current study are likely the Cluster/Signature 3 and 4 identified by Sarni et al. (mid/late regions associated with CFSs with delayed RT), and the Cluster/Signature 5 identified by Sarni et al. is similar to the Class-5 in this study (early region with advanced RT).

• 1b. The association of Class-4 breaks with RT transition regions and A/B compartment boundaries is a well-documented feature of CFSs.

• 1c. Class-5's enrichment for transcription-replication conflict markers, such as R-loops and G quadruplexes, reflects known characteristics of early RT regions rather than a novel mechanistic insight.

The manuscript fails to provide compelling evidence that these findings represent significant advancements beyond what is already known.

2. Insufficient Categorization of Chromosome Breaks

It has been well-documented that different replication stresses can generate different types of copy number alterations (CNAs) at different genomic contexts. Notably, in the study by Shaikh et al., separating CNAs identified by scWGSs into different categories allowed them to show that low doses of aphidicolin treatment favor large CN losses with recurrent breaks associated with cell type-specific CFSs. The analysis in the current study lacks granularity, as all chromosome breaks are grouped together without distinguishing break types (e.g., gains vs. losses, small vs. large CNAs). This approach obscures potential mechanistic differences and makes it difficult to contextualize the findings relative to prior studies. Without a more refined categorization, the results offer limited interpretative value.

As already pointed out by the authors in the current manuscript, “Recently, using scRepli-seq technology, large (>20 Mb) and small (<20 Mb) replication stress-induced CNVs were identified in single mammalian cells (Ref 12). However, only large CNVs were found to display features of CFSs, suggesting there could be other yet-to-be defined categories of fragile sites”. However, it is unclear how, without performing any distinction of breakpoints as did in the current manuscript, the authors can better address this question comparing the previous study.

3. Insufficient Evidence for Key Claims

Several major conclusions, including the identification of Class-4 and Class-5 as distinct categories, are inadequately supported:

• 3a. Class-4 breaks likely represent extensions of CFS regions rather than a novel break type. This interpretation aligns more closely with prior literature, as features such as delayed RT and association with TTRs are hallmarks of CFSs.

Based on Figure 2F, Class-4 showed similar transcriptional activity as Class-2 with no significant difference. Based on

Figure 3C, the RT slope of Class-4 (with delayed RT) is also similar to that of Class-2. Importantly, Figure 3F shows that the Class-4 breaks are located at the extremities of CFSs (based on the MiDAS signals). Therefore, it is more likely that Class-4 and Class-1/Class-2 breaks indeed result from the same phenomenon, i.e. the breaks associated with CFSs, which are large mid- to late-replicating regions poor in replication initiation induced by large active genes. The difference is that Class-1/Class-2 correspond to the core regions of CFSs (frequently around the gene centers), which show strong MiDAS signals (population average) and recurrent breaks. While the under-replicated regions within CFSs in aphidicolin-treated cells can display certain degrees of variation in the cell population, they can also extend outside of these CFS core regions (e.g. closer to gene extremities) and induce chromosome breaks, although with lower frequency. This can explain all the observed results shown in the current study. In agreement, Figure 3D shows that Class-4 (delayed RT) breaks show higher MiDAS signals than random control, although at a level much lower than that of Class-2.

Additionally, Sarni et al. (Nat. Commun. 2020) have shown that an important feature of CFSs is comprised of a TAD boundary (frequently at gene extremities) overlapping a highly transcribed large gene with aphidicolin-induced RT-delay. Considering that TTRs are frequently associated with transition regions between A/B compartments, it is not surprising that the authors find an association of Class-4 breaks (frequently located at extremities of large active genes poor in replication initiation, therefore, TTRs) coinciding with A/B compartment boundaries.

• 3b. The resolution of scWGS (500 kb) limits the ability to attribute transcription-replication conflict features specifically to Class-5 breaks. The observed associations appear to be driven by replication timing rather than a distinct mechanism.

It is well known that both R-loops and G quadruplexes are enriched at early-replicating regions, Therefore, it is not surprising that the authors find an enrichment of these features at Class-5 breaks and not others. Although the authors showed a difference between the Advanced-RT and Delayed-RT groups of Class-5 breaks, it lacks a propre control analysis to evaluate the significance and interpretation of such difference. Based on Figure 4A, it is clear that the enrichment of R-loops and G quadruplexes is tightly linked to replication timing (Class-5 > Class-2/4 > Class-1/3). If Advanced-RT Class-5 breaks have an earlier RT than the Delayed-RT Class-5 ones, it can well explain the observed results shown in Figure 4A. The authors need to evaluate/analyze the RT distribution of each sub-group for comparison.

4. Methodological and Statistical Rigor

4a. The manuscript relies heavily on descriptive observations and lacks the statistical rigor required to substantiate its claims. Key analyses (e.g., enrichment of R-loops and G quadruplexes in Class-5) do not include appropriate controls or statistical tests to evaluate significance. Randomization or permutation tests taking into account the RT distribution of each subgroup (to get a P-value to show that whether the observed Z-score is significant or not) would be necessary to rule out artifacts.

4b. Similar criticism applies to the results showed in Figure 5. Please provide the RT distribution for each subgroup. If in each RT fraction (i.e. from S2 to S9) the Advance-RT group has the earliest RT while the Delayed-RT group the latest RT, this can explain the results shown in Figure 5C, as all these features show higher signals at the earlier RT regions. Statistical evaluations and P-values, therefore, need to be provided for the figures.

PS: it seems that the percentage of Advanced and Delayed RT regions are very high in each RT fraction (comparing to those reported by Sarni et al., 2020). I wonder whether this is due to the authors not setting any cutoff to define the Advanced and Delayed RT regions (Page 11, "we identified genomic regions that shifted fractions (either earlier or later) following aphidicolin treatment as advanced or delayed RT regions"). This might capture stochastic variations in RT data between the two conditions. If the authors restricted their analyses to regions with significant RT swifts, would they observe similar or different results?

4c. In Figure 5, the authors show the numbers and percentage of breaks in 5 Classes in PRE-1 cells (B,C), BJ cells (D,E) and mESC cells (G). However, without a control analysis, it is hard to evaluate whether the obtained results signify some biological meaning. The authors need to generate random simulation data with similar RT distribution to check whether the observed results are significantly different from random expectation. This is particularly important considering that the CFSs of U2OS cells were used in the figure, and we know that CFSs are cell type specific.

5. Limited Mechanistic Insights

The study largely recapitulates known features of aphidicolin-induced replication stress without addressing critical unresolved questions in the field. For example:
• What mechanisms drive the formation of specific break classes? For example, comparing data obtained in mutant cells (as in Shaikh et al., 2022) to support the role of a gene/pathway in the formation of specific break classes.
• How do these findings advance our understanding of replication stress-induced fragility beyond previous work?
The lack of mechanistic depth significantly undermines the manuscript's potential impact.

Minor comments:

- 1. “To determine the cell-cycle duration of U2OS cells, cells were released from a double thymidine block with or without low-dose aphidicolin,”

Please indicate, in the main text, the concentration of aphidicolin used in the current study to avoid requiring readers to check the figure or method.

- 2. Page 3, “Based on the 2D cell-cycle FACS, single G1 cells were sorted and scRepli-seq was performed as described16 (Fig. 1A, Experiment).”

Based on the experiment protocol, it’s indeed a classical scWGS of G1 cells (exactly the same principle as Shaikh et al. 2022). Therefore, please modify the description of this section and the corresponding figures to avoid any confusion. scRepli-seq allows obtaining information on DNA replication timing, while here the scWGS data identify scCNAs and will not provide information on RT.

- 3. Page 4, “karyotype heterogeneity increased as judged by visual inspection (Fig. 1B, compare top and bottom panels), which was statistically confirmed by analyzing the degree of cell-to-cell karyotype heterogeneity (Fig. 1C).”

Please provide the P-values and the corresponding test to support the statement of “statically confirmed”.

- 4. Page 4, “We then refined the breakpoint position to be close to the actual breakpoint position using uniquely mapped reads and calculated a 95% confidence interval (CI) for each refined chromosome breakpoint, which we defined as a chromosome break region (or break region for short) (Fig. 1A, Data analysis)”.

It is unclear how this process was performed. Please provide more details in the Methods section. What is the resolution/precision of the refined breakpoint positions? Is it much better than 500-Kb (the bin size used for the breakpoint identification)?

- 5. Page 5, “Aphidicolin-induced break regions had a median size of 0.27 Mb and 76% of the break regions were less than 0.5 Mb in size (Supplementary Fig. 1C)”.

If I understand correctly, this is the size of regions enriched with aphidicolin-induced specific breakpoints, and not the size of CNAs associated with aphidicolin-induced breaks. Therefore, it might better to avoid using “regions” to avoid confusion.

- 6. Instead of focusing directly on Aphidicolin-induced breaks, the authors should first characterize and compare breaks identified in non-treated cells versus Aphidicolin-treated cells. For instance, details on the type and frequency of CNAs (gains vs. losses), their size distribution, and their RT distribution would provide a clearer context for understanding features specific for aphidicolin-induced breaks.

- 7. Page 4, “We found that 38% (103/274) of all break regions corresponded to CFSs, which was statistically significant (p = 0.001; vs. 1000 randomized control regions).”

Did the generation of randomized control regions take into account the RT distribution as in some other randomized control analyses? If not, is this result still signification when considering RT distribution of the break regions?

- 8. Page 4. “Even for this comparison, 39% (107/274) of our chromosome break regions corresponded to CFS break regions, indicating that our experimental and analytical setup using scRepli-seq safely captured known CFSs”.

What about the numbers and percentages of CFS regions (e.g. identified by MiDAS-seq) associated with chromosome breaks identified in the current study? Please provide and interpretate this analysis.

- 9. Page 5. “The 10-cell Repli-seq log2repliscore RT values of all 14 S-phase fractions were averaged to obtain a ‘pseudobulk’ genome-wide RT profile (Fig. 1E, Supplementary Table 2),”.

If only pseudobulk RT data were used in this analysis, what additional information does it provide compared to population-based RT data (e.g. classical Repli-seq)? It seems that scRT data, generated using a more complicated protocol, was reduced to pseudobulk RT data in the final analysis. Does this imply scRT data added no advantages?

- 10. Page 5, “Class 1, late-S RT CFS (30%); class 2, mid-S RT CFS (12%); class 3, late-S RT non-CFS (9%); class 4, mid-

S RT non-CFS (20%); and class 5, early-S RT non-CFS (29%)".

In addition to percentage, please include the actual count of breaks (n) in each class.

- 11. Fig. 2B, the amount of Advance-RT (~ one third) group in Class-1 is much higher than that of Class-2 (only a few). Any explanation?

- 12. Fig. 2E, the numbers of break regions indicated in this figure agree with the numbers shown in Fig. 1G, however, they are different compared to the numbers shown in Fig. 2 (B-D,F-G). Please provide an explanation for these differences and ensure consistency across all figures.

PS: move Fig. 2E into Fig. 1 since it seems that they belong to the same object.

- 13. Page 5, "The degree of RT delay is correlated well with aphidicolin-induced MiDAS-seq signal intensity (Supplementary Fig. 3A), suggesting that aphidicolin-induced RT changes can be used to predict CFSs".

This observation aligns with numerous prior studies (e.g., Sarni et al., 2020). Please rephase it to better appreciate the exiting literatures in the field and avoid suggesting this is a novel finding.

- 14. Page 6. "we performed a more simple and straightforward experiment, namely BrdU-IP of G2/M-phase cells followed by sequencing, or G2/M-seq, which directly measured BrdU incorporation, i.e., DNA synthesis in G2/M (Supplementary Fig. 3B)".

Please provide additional details on the difference between G2/M-seq and MiDAS-seq. What are the advantage and limitations of each?

Based on Fig. 2C, G2/M-seq signals are much stronger for Class-2 than Class-1, whereas MiDAS-seq signals are comparable for both (Fig. S3C). Could the authors explain this difference?

- 15. Page 6. "Moreover, they were frequently observed in multiple single cells, strongly suggesting that they are recurrent events (Fig. 2E)".

Please provide a statical test to substantiate this claim.

- 16. Page 7, "more so than class-3 RT-delayed breaks".

Typo? Should be "more than…".

- 17. Page 7. "To test this hypothesis, we first identified the local minimum positions of RT (i.e., termination zones, TZs) in untreated conditions (Supplementary Fig. 3G), and TZs that replicated in late-S phase (RT < –0.25) were selected. Then, we performed a K-means clustering of RT surrounding these late-S TZs into two groups, which distinguished TZs with narrow RT valleys (cluster-1) from those with broad RT valleys (cluster-2) (Supplementary Fig. 3H)".

Please provide additional details in the Methods section regarding how local minimum positions of RT were identified.

Based on the data shown in Supplementary Fig. 3H, only a small number of TZs were identified (336 in Cluster-1 and 395 in Cluster-2). Clarify whether this is expected. If this number is unusually low or high, explain the underlying reasons.

- 18. Page 7. "In support of our hypothesis, cluster 1 but not 2 clearly showed enrichment for aphidicolin-induced G2/M replication, suggesting that narrow late-S RT valleys tend to remain under-replicated upon aphidicolin treatment (Supplementary Fig. 3H)".

Based on Figure 2G, three classes of late regions were identified. Both Class-1 and Class-3 (RT-delayed) exhibit narrow late-S RT valleys, while Class-3 (RT-advanced) shows broad RT valleys.

Please clarify whether Cluster-2 in Supplementary Figure 3H corresponds to Class-3 RT-advanced regions, and whether Cluster-1 includes both Class-1 and Class-3 RT-delayed regions. If this is the case, refine the clustering analysis (e.g., use three clusters) to better align with the three categories in Figure 2G.

- 19. Page 12, "We found that RPE1 and BJ cells also increased their karyotype heterogeneity upon aphidicolin treatment

(Supplementary Fig. 5A,B,C)".

Please provide a quantification to support this statement.

- 20. Page 12, "We identified aphidicolin-induced break regions from 329 single aphidicolin-treated RPE1 cells, with 284 having unique genomic positions. In BJ cells, 244 aphidicolin-induced break regions were identified from 173 single aphidicolin-treated cells, with 165 having unique genomic positions (Fig. 6A, Supplementary Table 1)".

Again, before discussing aphidicolin-induced break regions, describe the total number of breaks or regions identified in untreated versus aphidicolin-treated conditions for both RPE1 and BJ cells. This contextual information is critical for understanding the scope and specificity of aphidicolin-induced breaks.

- 21. Page 16, "We utilized one end of the paired-end sequenced reads".

It is unclear why not using the paired-end reads for analyses.

- 22. Page 20, "10-HMM RT was then computed from pseudo-bulk RT using RHmm v2.2.1".

Please provide more details about this computation. Specify the parameters used, the type of HMM model applied, and the matrix used for training or testing the HMM model.

- 23. Page 21, "HiC-Pro v3.1.0 was used to generate contact matrix from fastq files (Ref39) from which A/B compartment profile was calculated using Cooltools v0.5.4 (Ref 40)".

Please include details about the parameters used for both HiC-Pro and Cooltools.

- 24. Page 21, "Generation of randomized control regions".

Please clarify how the random simulations were performed. Specifically, describe how replication timing distribution was incorporated into the simulations, as this detail directly affects the robustness and interpretation of the results.

- 25. Page 22, "In all the boxplots, the whiskers (lines extending from the box) represent the minimum and maximum values in the dataset, excluding any outliers".

Explain how outliers were defined in the dataset. Include a clear definition or threshold to ensure transparency.

- 26. Page 22, "The computer codes are available from authors upon reasonable request".

In line with open science practices, please submit the codes to a publicly accessible repository.

Reviewer #3

(Remarks to the Author)
In this manuscript, the authors utilize a single-cell based DNA replication sequencing to map chromosomal breakage under replication stress. They categorize these identified breaks into different classes based on replication timing (RT) and location of CFSs, which further allows for a precise and comprehensive analysis of their genomic features, relative frequency, RT landscape and potential mechanisms. Overall, the findings in this paper are of interest and should offer valuable insights to further research in the field, but the mechanism is not clear enough. Nevertheless, several points and criticisms warrant attention and further clarification.

Major concerns:
1.Historically, fragile sites have been defined as site-specific gaps or breaks observed in mitotic chromosomes. However, in this study, the authors define chromosome breaks based on copy number variation in G1-phase cells under replication stress. Since they have not confirmed whether these breaks actually occur during mitosis, it seems premature to equate these breaks with fragile sites at this stage. I suggest further investigation to confirm the mitotic occurrence of these breakages.
2.The origin density and sequence characteristics of the different classes of break regions should be analyzed and discussed in more detail. The lack of replication origins and a high AT content are known to contribute significantly to CFS fragility. Addressing these aspects could strengthen the conclusions regarding the underlying mechanisms of breakage formation.
3.As class-5 break regions show early-replicating and no enrichment for aphidicolin-induced G2/M-seq signals, do they correspond to early replicating fragile sites? A correlative analysis may help to answer this question.

4.The authors suggest that class-5 breaks with advanced replication timing display characteristics of transcription-replication conflicts (TRC) based on public datasets. However, additional experimental validation is needed to determine whether TRC is the primary driver of these breaks. For example, regulating TRC levels through transcription inhibition and assessing breakage formation would provide more direct evidence.
5.It's been reported that CFS stability is compromised at TAD boundaries (PMID: 32680994), what's the relative location of TAD boundary with different classes of breakages? Did TAD organization affect breakage formation?

Minor comments:
1.Supplementary Figure 3: the authors analyze replication activity through BrdU-seq of G2/M-phase cells based on DAPI staining. However, this method cannot completely exclude late S-phase cells, which also exhibit G2/M DNA content. To be more precise, it would be better to describe this as "BrdU-IP of late S/G2/M-phase cells" to more accurately reflect the experimental approach.
2.The sorting strategy used in CBMS1 mouse embryonic stem cells for scRepli-seq and G2/M-seq should also been presented.

Reviewer #4

(Remarks to the Author)
I co-reviewed this manuscript with one of the reviewers who provided the listed reports. This is part of the Nature Communications initiative to facilitate training in peer review and to provide appropriate recognition for Early Career Researchers who co-review manuscripts.

Version 1:

Reviewer comments:

Reviewer #1

(Remarks to the Author)
The authors have mostly addressed the comments raised by the Reviewers.

From my side there were two main points: whether the classification of aphidicolin-induced breaks leads to more types than supported by the real biology and the limited information on mechanistic insights. The authors present 5 types, 3 of which have been described below (late S-replicating CFS; mid S-replicating CFS; early S-replicating non-CFS). The last type is shown in this revised manuscript to correspond to the early-replicating fragile sites (ERFS) described by Nussenzweig et al several years ago. The remaining 2 types (mid S-replicating non-CFS and late S-replicating non-CFS) appear to be novel.

Regarding mechanistic insights, the authors describe new experiments treating cells with DRB to inhibit transcription elongation. These results argue that early S-replicating non-CFS sites (Fig. 5C) are due to transcription-replication conflicts. The mid S-replicating non-CFS sites (Fig. 4B) behave very similar to the early S-replicating non-CFS sites (Fig. 5C) and may be mechanistically similar. The surprising observation relates to the late S-replicating non-CFS sites, where aphidicolin does not induce a DNA damage response, as would be expected; instead DRB induces DNA damage (Fig. 3D). I am sceptical of these data. The authors should discuss this finding. It could be that early S-replicating, mid S-replicating and late S-replicating non-CFS breaks are all due to transcription-replication conflicts. The data suggest that this is true for early S and mid S non-CFS breaks. For late S non-CFS breaks, the number of breaks was probably low (as suggested by Fig. 1G) and the collected data may not be accurate, as they do not make sense.

Overall, the manuscript has been improved and can be published. Ideally, the issue of Fig. 3D should be addressed. If the authors are not able to generate reliable data, they should not show these data.

The journal could be Nature Communications or Genome Biology as decided by the editors.

Reviewer #2

(Remarks to the Author)

Compared with the previous version, the revised manuscript by Elumalai and Hiratani has been significantly improved. The authors have added new analyses and experimental data, including a clearer comparison between late-S/G2/M-seq and MiDAS-seq, improved comparisons between 10-cell Repli-seq and conventional two-state BrdU-IP RT profiling, additional analyses of break types, and more systematic characterization of non-CFS classes relative to canonical CFSs. The manuscript also includes improved comparisons with previous studies and new experiments examining the effect of transcription inhibition. Overall, these additions strengthen the manuscript. However, several important issues still need to be addressed and clarified before the manuscript can be further considered.

1. Comparison between MiDAS-defined CFSs and Aph-induced breaks

Based on the revised analysis, only 16% of MiDAS-seq CFSs overlap with Aph-induced breaks identified in this study (Fig. S1F). This relatively low overlap requires further explanation.

On the other hand, the overlap between Aph-induced breaks identified in U2OS cells in this study and cytogenetically identified CFSs from fibroblasts appears to be higher (Fig. S1F). This is somewhat unexpected, as it is well established that CFSs are cell-type specific. In principle, datasets generated from the same cell type should show higher concordance.

A possible explanation could be differences in resolution between cytogenetic CFS mapping and sequencing-based datasets. However, this discrepancy is not discussed in the manuscript. The authors should explicitly address this issue and discuss potential explanations.

2. Potential bias introduced by excluding "inherent breakpoints"

The authors define aphidicolin-induced break regions as those that do not overlap with, or lie within ±1 Mb of, untreated break regions, in order to remove "inherent breakpoints".

However, it is unclear how this filtering step may affect downstream analyses. Importantly, inherent breakpoints present in U2OS cells are unlikely to be randomly distributed across the genome and may themselves correlate with replication timing or other genomic features. Therefore, excluding all regions within ±1 Mb of untreated breakpoints could potentially introduce biases into the analyses of aphidicolin-induced breaks.

As raised previously, the authors should perform a systematic characterization of inherent breakpoints, including their genomic distribution and their relationship to MiDAS-defined CFSs and RT profiles, before excluding them from downstream analyses. This would be necessary to demonstrate that the filtering strategy does not introduce unintended biases.

3. Clarification of whether and how RT was controlled in the randomized controls

In several analyses (e.g., Fig. S3F and Fig. 7F), randomized genomic regions are used as controls. However, it is not clearly stated whether the replication timing (RT) distribution of breakpoints was taken into account during the randomization procedure.

Because many genomic features analyzed in this study are strongly correlated with RT, it is critical that the randomized controls match the RT distribution of the observed breakpoints. Otherwise, apparent enrichments or depletions may simply reflect RT bias.

The authors should explicitly clarify whether RT was incorporated into the randomization strategy for these analyses. If RT was not taken into account, the relevant analyses should be repeated using randomized controls that preserve the RT distribution of the observed breakpoints.

A similar control analysis would also be required for the analyses involving the mESC datasets.

4. The current randomization strategy does not appear to adequately control RT

Related to the point above, the description of the randomization procedure in the Methods suggests that RT is not directly controlled during randomization.

According to the Methods, randomized regions were generated using bedtools shuffle, followed by classification of these regions into early/mid/late S-phase using pseudobulk RT profiles. However, this procedure represents post hoc categorization rather than RT-matched randomization.

In other words, the randomized regions appear to be generated genome-wide and only afterward assigned to RT categories, which does not ensure that the RT distribution of the randomized controls matches that of the observed breakpoints.

Because replication timing is a major determinant of many genomic features analyzed in this study, failure to properly control RT in the randomization procedure could substantially affect the interpretation of the results.

The authors should clarify this point and, if necessary, revise the randomization strategy to ensure that the RT distribution is properly controlled in analyses relying on these randomized controls.

5. Interpretation of transcription inhibition experiments

The newly added transcription inhibition experiments are interesting, while several aspects require further clarification.

In the Immuno-FISH analysis, the authors quantify the "logarithmic γH2AX signal per FISH spot per nucleus". However, the method used to calculate this value is not clearly described. It is unclear whether the logarithmic transformation was applied

to raw intensity values or to normalized intensities (e.g., relative to nuclear signal). This information should be clarified.

In addition, the rationale for applying a logarithmic transformation is not explained. While log transformation may be useful for handling skewed intensity distributions, it would be helpful to know whether the conclusions remain consistent when using the raw or normalized intensity values.
Given that most values shown in the figures (e.g., Fig. 3C/D, Fig. 4B/C, Fig. 5C) fall within a relatively narrow range (approximately −1 to 1 on the $\log_2$ scale for CFS-L and −0.5 to 0.5 for the other classes), it would also be informative to assess whether the observed differences remain evident when plotted using the original intensity values.

Additionally, in the Immuno-FISH analysis, DNA-FISH signals were identified using the “Find Maxima” function in Fiji. Because this approach detects local intensity maxima and can be sensitive to noise and background fluctuations, it would be helpful if the authors could provide additional details on the parameters used (e.g., noise tolerance) and how the robustness of spot detection was ensured.

6. Further clarification of transcription inhibition experiments

In the Immuno-FISH analysis, asynchronous U2OS cells were treated with aphidicolin for 24 h, with DRB added during the last 4 h to inhibit transcription. The results show that transcription inhibition has little effect on γH2AX levels at CFS-associated classes but strongly alters γH2AX levels at non-CFS-L regions.

Several aspects of this experiment require further clarification and discussion.

First, the cell-cycle distribution of the analyzed population after this treatment regime is not described. Given the prolonged aphidicolin treatment and the asynchronous cell population, the cell-cycle composition could strongly influence the observed γH2AX signals.

Second, the authors’ analyses indicate that non-CFS-L loci exhibit relatively low transcription levels (e.g., GRO-seq in Fig. 2F). It is therefore not immediately obvious why transcription inhibition would strongly affect γH2AX levels at these regions. This point should be further discussed.

Third, DRB treatment alone appears to increase γH2AX levels at non-CFS-L loci, raising the possibility that the observed effects may arise from indirect consequences of transcription inhibition, such as alterations in cell-cycle progression or replication programs. These possibilities should be carefully examined and discussed.

7. Interpretation of predictive performance

In Fig. 6C, the reported AUC values range from approximately 0.6 to 0.75. While these values suggest some degree of predictive signal, they generally indicate only modest discriminatory power. The current wording in the manuscript may therefore overstate the predictive performance of the model, and the corresponding claims should be moderated accordingly.

In addition, each model appears to be represented by a single AUC estimate without an associated confidence interval or measure of variability. Because AUC estimates can depend on sampling variation, it would be helpful to report uncertainty estimates (e.g., bootstrap-based 95% confidence intervals or cross-validation results). This would allow readers to better assess the robustness of the reported predictive performance and the significance of differences between models.

8. Use of the term “rare fragile site”

The term “rare fragile site” (used in the Abstract) already has a well-defined meaning in the fragile site field (e.g., FRAXA associated with fragile X syndrome). Using this term to describe the break classes identified in this study may lead to confusion. The authors should avoid reusing terminology that already has an established definition in the field.

9. Clarity of terminology related to scRepli-seq and scWGS

I understand that the authors may wish to emphasize the previously developed scRepli-seq framework. However, the current wording may create confusion regarding the method used in this study.

For example, the Introduction states: “To perform an unbiased, comprehensive mapping of fragile sites, we employed scRepli-seq for karyotyping…” In practice, break detection in this study is based on single-cell whole-genome sequencing (scWGS) of G1 cells, which is conceptually distinct from replication timing profiling. For clarity, this sentence could be revised to explicitly state that scWGS (implemented within the previously developed scRepli-seq framework) was used for karyotyping and break detection.

Similarly, the Results section title “Single-cell karyotyping by scRepli-seq” would be more accurately described as “Single-cell karyotyping by scWGS of G1 cells”.

The authors should revise related descriptions throughout the manuscript, including figure legends where appropriate.

I hope that these comments and suggestions will help further improve the rigor and reproducibility of the manuscript.

Reviewer #3

(Remarks to the Author)
Upon my initial evaluation, I recognized the technical rigor of this study, notably its application of scRepli-seq to comprehensively map and characterize chromosomal breakage under conditions of replication stress. While the findings hold relevance for the field, my central concern—namely, the absence of a clear mechanistic framework—has not been satisfactorily resolved in the current revision.

The revised version remains predominantly descriptive. It effectively delineates correlations between genomic features and classes of breaks, yet it does not advance beyond these associations to establish causality or to formulate and validate a novel regulatory model. Although the analyses are executed thoroughly, they principally serve to confirm or extend established paradigms connecting replication timing, transcription, and sequence features with genomic fragility.

The authors have included a comparative analysis of their APH-induced breakpoints with cytogenetic fragile site data from Wilson et al. (2015). While the reported 45% overlap is suggestive, this validation remains indirect and relies on cell-line mismatches. A more definitive approach would entail performing cytogenetic validation (e.g., metaphase spreads) specifically in U2OS cells under identical experimental conditions, or aligning the data with existing U2OS-specific cytogenetic maps. Direct evidence obtained in a matched cellular context is essential to robustly bridge the high-throughput sequencing findings with the classical cytogenetic definition of mitotic fragile sites. I recommend the authors address this point either through additional experiments or, if further cytogenetic validation is not feasible, through a more thorough discussion of this limitation.

Despite the authors' efforts during revision, the study still falls short of the required threshold for novelty and mechanistic depth. Therefore, I would not make a strong case for Nature Communications, though it may be more appropriately aligned with the scope of Genome Biology.

Reviewer #4

(Remarks to the Author)
I co-reviewed this manuscript with one of the reviewers who provided the listed reports. This is part of the Nature Communications initiative to facilitate training in peer review and to provide appropriate recognition for Early Career Researchers who co-review manuscripts.

Version 2:

Reviewer comments:

Reviewer #2

(Remarks to the Author)
The authors have made substantial efforts to address my previous comments and have provided additional analyses and experimental data that improve the manuscript. In particular, the inclusion of analyses on inherent breaks and additional Immuno-FISH FACS data is appreciated. However, several mechanistic interpretations remain speculative and are not yet directly supported experimentally. The discussion should more clearly distinguish between experimentally supported conclusions and proposed interpretations. The following important issues still require clarification before several of the central conclusions can be fully supported.

Major comments

1. Interpretation of aphidicolin-dependent break classes

I appreciate that the authors now included the analysis of inherent breaks from U2OS cells. Comparing the proportions of aph-induced breaks (N = 274) and inherent breaks (N = 3316) with randomized controls for different break classes, it is clear that only the CFS-L and CFS-M classes show stronger enrichment in aph-induced samples relative to inherent breaks, which is consistent with the established aphidicolin dependency of canonical CFSs.

However, the current analyses demonstrate the existence of multiple break classes among both aph-induced and inherent breaks, but they do not yet clearly establish that the non-CFS classes are preferentially induced by aphidicolin, rather than reflecting constitutive fragile regions with limited detection sensitivity in untreated samples because of the smaller sample size. Additional discussion is therefore needed to address this important point. In particular, the later Immuno-FISH

experiments do not sufficiently demonstrate that these classes represent bona fide aph-dependent break events (see next comment).

To facilitate comparison, the percentage of inherent breaks should be added to the bar plot in Supplementary Fig. 3H alongside aph-induced breaks and randomized controls. Similar analyses should also be included in Fig. 7F for RPE1 and BJ cells, as well as Supplementary Fig. 10D for mESCs.

2. Potential confounding effects of altered cell-cycle distribution in Immuno-FISH experiments

The newly added FACS profiles in Supplementary Fig. 5H are useful. I agree that DRB treatment itself does not appear to substantially alter the cell-cycle distribution in either aph-treated or untreated cells. However, aphidicolin treatment clearly changes the cell-cycle profile, with substantial accumulation of cells in S and G2/M phases. This issue should be carefully considered when interpreting the Immuno-FISH results. At minimum, the limitations introduced by altered cell-cycle composition should be more explicitly acknowledged when interpreting γH2AX enrichment as evidence of aphidicolin-dependent break formation.

For example, for the non-CFS-M RT-delayed breaks (Fig. 4B), the authors propose that these breaks arise from transcription-replication conflicts. If so, stronger γH2AX signals would be expected preferentially in S-phase cells. Therefore, it is unclear whether the increased γH2AX signals observed in aph-treated samples simply reflect the higher proportion of S-phase cells, rather than increased break formation specifically induced by aphidicolin. Without additional controls accounting for cell-cycle composition, it remains difficult to conclude whether these represent genuinely aphidicolin-induced break events.

A similar issue applies to the non-CFS-L RT-delayed breaks (Fig. 3D). If DRB-dependent breaks are enriched in S or G2/M cells, the stronger γH2AX signals observed in the Aph+DRB condition relative to DRB alone could potentially be explained by altered cell-cycle distribution rather than a direct effect on break formation.

3. Presentation of raw γH2AX signals

With the additional explanations, I now better understand how the Immuno-FISH data were processed. Since the authors have already performed these analyses during revision, inclusion of the raw intensity plots in the manuscript would substantially improve transparency and interpretability.

The raw data provide more direct and informative comparisons between conditions without requiring additional normalization procedures. Importantly, they also reveal biologically relevant features that are obscured in the normalized presentation. For example, raw γH2AX signals appear lower for BAC probes located in late-replicating regions (e.g., CFS-L_RT-delayed and nonCFS-L_RT-delayed regions) compared with probes in mid- or early-replicating regions (if we compare the untreated samples). This observation could reflect either biological differences or technical limitations, and the authors should comment on and discuss this point.

4. Overstatement of predictive power throughout the manuscript

There are still numerous instances where the manuscript overstates the predictive performance of the analyses. In several cases, the analyses are better described as demonstrating associations or partial discriminatory power rather than prediction. This issue should be carefully revised throughout the text.

For example, in the sections entitled “Genomic/epigenomic features can predict aphidicolin-induced RT changes” and “Late-S RT landscapes can predict the replication behavior in response to aphidicolin”, the presented analyses mainly demonstrate positive or negative correlations. However, no true predictive framework has been established, nor has any rigorous predictive modeling analysis been performed to demonstrate substantial predictive power for aphidicolin-induced RT changes. These analyses are largely correlative, and the current data do not yet establish predictive performance. Accordingly, the authors should revise the text throughout the manuscript to remove or substantially tone down wording such as “predict”, “predictive”, and “predictable” in both Results and Discussion.

In addition, strong statements such as:
• “Intriguingly, we found that the RT landscape could predict aphidicolin-induced RT behavior and break propensity” (Abstract)
• “Moreover, we demonstrated that the RT landscapes in untreated cells enable prediction of aphidicolin-induced RT behavior and chromosome break propensity” (End of the Introduction)
should either be removed or substantially revised. Based on Fig. 6C, the predictive performance is only modest even for the CFS-L class (and to a less extent for non-CFS-L_RT-delayed class), while predictive performance appears limited for the other classes.

Minor comments

1. Page 2: “Recently, using single-cell whole genome sequencing (scWGS) adapted from scRepli-seq technology…(Ref. 13)”
Please remove “adapted from scRepli-seq technology”, since the scWGS method used in Ref. 13 was not adapted from scRepli-seq technology.

2. The order of Supplementary Notes should be revised throughout the manuscript. For example, Supplementary Note 3 is cited before Supplementary Note 2, and Supplementary Note 7 appears before Supplementary Note 6.

3. Page 7: “They were present closer to long genes (>=300 kb) compared to randomized control with similar RT (Fig. 2B; see Methods)”, Please also cite Supplementary Fig. S5A here.

Reviewer #3

(Remarks to the Author)
The authors have addressed several points raised in the previous round, notably the concern regarding cell-type specificity of cytogenetic fragile sites. They have provided additional discussion and comparisons across multiple cell lines, which I find acceptable. Compared to the previous version, the study has been substantially improved, and the revised analyses sufficiently support the main conclusions. Considering the technical rigor of the work and the fact that the findings will be of interest to the genome stability community, I believe the manuscript can now be considered for publication.

Reviewer #4

(Remarks to the Author)
I co-reviewed this manuscript with one of the reviewers who provided the listed reports. This is part of the Nature Communications initiative to facilitate training in peer review and to provide appropriate recognition for Early Career Researchers who co-review manuscripts.

**Open Access** This Peer Review File is licensed under a Creative Commons Attribution 4.0 International License, which permits use, sharing, adaptation, distribution and reproduction in any medium or format, as long as you give appropriate credit to the original author(s) and the source, provide a link to the Creative Commons license, and indicate if changes were made.
In cases where reviewers are anonymous, credit should be given to 'Anonymous Referee' and the source.
The images or other third party material in this Peer Review File are included in the article’s Creative Commons license, unless indicated otherwise in a credit line to the material. If material is not included in the article’s Creative Commons license and your intended use is not permitted by statutory regulation or exceeds the permitted use, you will need to obtain permission directly from the copyright holder.
To view a copy of this license, visit https://creativecommons.org/licenses/by/4.0/

Thank you very much for handling our manuscript entitled "Unbiased genome-wide mapping and characterization of fragile sites in single mammalian cells" (please note that the title has been revised to "*Single-cell mapping of chromosome breaks identifies multiple fragile site classes with distinct DNA replication timing landscapes*"; see **Summary and Highlights, #3**). We appreciate the constructive and insightful feedback provided by the reviewers. We have carefully addressed all reviewer comments, incorporating additional analyses and experiments where required. As detailed below, the revised manuscript strengthens our conclusions regarding the newly identified categories of chromosomal breaks. It also demonstrates the mechanistic differences among these distinct break categories (see **Summary and Highlights, #1a and #2**). We note that several major concerns raised by Reviewer 2 stemmed from misunderstandings of prior literature or of our analytical framework; we have also clarified these points in detail in our response and believe that the issues can now be fully resolved. In addition, we have also extended our earlier finding regarding the relationship between replication timing (RT) landscapes and aphidicolin-induced RT changes; in particular, we made a fascinating discovery that local RT landscapes in untreated cells can predict aphidicolin-induced break-prone regions of the genome (see **Summary and Highlights, #1b**). In light of these substantial improvements, we respectfully request reconsideration of our manuscript for publication in *Nature Communications*. If it is deemed unsuitable, we would appreciate guidance on alternative transfer options (for example, *Genome Biology*) that best fit the scope and significance of the revised manuscript. If no such alternatives are recommended, we would be happy to accept the transfer offer to *Communications Biology*.

Below are the **Summary and Highlights** of our revised manuscript, followed by a point-by-point response to all comments raised by the reviewers (reviewer comments in black, our response in blue, and changes in the manuscript in red). We look forward to your response.

**Summary and Highlights** (common to all reviewers):
We thank the reviewers for their constructive feedback. As Dr. Hug described in her decision email, the major criticisms by the reviewers can be summarized in the following two points:
**(1) Lack of novelty and strength of our work and conclusions compared to earlier studies**
**(2) Lack of mechanistic insights into the break formation of various classes**
In response to these criticisms, we have conducted new experiments and analyses and have emphasized the following findings in our revised manuscript.

**Clarification of break class nomenclature**
Before presenting our new results, we acknowledge that the original numerical "class" nomenclature does not intuitively convey the biological characteristics of each break type. We therefore renamed class-1–5 breaks to reflect their relationship to common fragile sites (CFSs) and replication timing (RT). Specifically:

- Class-1: **CFS-L** (late-S replicating common fragile sites)
- Class-2: **CFS-M** (mid-S replicating common fragile sites)
- Class-3: **non-CFS-L** (late-S replicating non-common fragile sites)
- Class-4: **non-CFS-M** (mid-S replicating non-common fragile sites)
- Class-5: **non-CFS-E** (early-S replicating non-common fragile sites)

This revised nomenclature is more intuitive, consistent with prior literature, and used throughout the revised manuscript.

**(1) The novelty and strength of our conclusions compared to earlier studies are two-fold:**

**(a) We have successfully identified 'rare' fragile classes that are distinct from CFSs and ERFSs**

Single-cell mapping of aphidicolin (APH)-induced chromosome breaks allowed us to identify 'rare' fragile classes (non-CFS-L and non-CFS-M) that are distinct from common fragile sites (CFSs, corresponding to CFS-L and CFS-M) and early-replicating fragile sites (ERFSs; during the revision phase, we found that ERFSs were physically close to our non-CFS-E breaks). Non-CFS-L and non-CFS-M breaks did not exhibit features of CFSs such as being close to long genes (former Fig. 2D; now Fig. 2B), active transcription (Fig. 2F, break sub-classes are now plotted separately), or enrichment for FANCD2 binding (former Fig. 4A; now Fig. 5B). They also did not correspond to ERFSs, as they did not show early-S replicating timing (RT) or enrichment for R-loops and G4 repeats (former Fig. 4A; now Fig. 5B). We have added new data to show that non-CFS-L and non-CFS-M breaks are neither extensions of CFSs (Fig. 2E) nor associated with large CNVs (feature of CFSs, Supplementary Fig. 4A), which further support our claim that non-CFS-L and non-CFS-M breaks are distinct from CFS-L, CFS-M, and non-CFS-E breaks.

**(b) We have demonstrated that RT landscapes in untreated cells (i.e., before APH treatment) are predictive of APH-induced RT changes and break propensity**

Previous studies have primarily focused on the direction of RT changes following APH treatment, which was also one of our focuses. In addition, we have discovered that the shapes/landscapes of RT in untreated cells (i.e., the RT landscape before APH treatment) can allow one to predict RT changes and the propensity for break formation upon APH treatment. Surprisingly, this observation was largely overlooked by the reviewers in the initial round. Therefore, we would like to elaborate on our findings once again and describe the new data added during the revision phase. In late S-phase, we found that steep RT valleys are a feature of CFS-L breaks, which remain under-replicated in G2/M upon APH treatment (former Fig. 2G, Supplementary Fig. 3H; now Fig. 3E,F). In mid-S, RT transition regions (TTRs) are a feature of non-CFS-M RT-delayed break regions (former Fig. 3A,B; now Fig. 4A,D). In early S-phase, non-CFS-E RT-advanced break regions, which exhibited features of transcription-replication conflicts (TRCs), displayed shallow RT landscape (former Supplementary Fig. 4D; now Supplementary Fig. 7E). We have added new prediction models using RT slope and curvature in untreated cells to show that RT landscapes are predictive of APH-induced break classes. Furthermore, stratifying the genome into multiple RT fractions in untreated cells revealed that genomic regions undergoing APH-induced RT advance or delay already deviate from the average genomic/epigenomic features of the same RT fraction (former Fig. 5C; now Fig. 6F). Together, these observations demonstrate that RT landscapes of untreated cells enable the prediction of APH-induced RT behavior and break propensities.

**(2) Our new data suggest that CFS-type and novel break classes form via distinct mechanisms**

To gain mechanistic insights into the chromosomal break formation of different classes, we examined the dependence of these break classes on transcription. Specifically, we used the transcription inhibitor DRB and measured γH2AX signals (DNA damage) at DNA-FISH signals corresponding to five break classes by immuno-FISH (Fig. 3A). We have shown that CFS-L, CFS-M, and non-CFS-E RT-advanced break regions were enriched for APH-induced FANCD2 (former Fig. 4A and now Fig. 5B). Taking advantage of this observation, we also analyzed the degree of colocalization of γH2AX and FANCD2 in different cell cycle phases (Fig. 5D). As a result, we found the following:

(a) APH-induced γH2AX signal intensity at CFS-L and CFS-M break sites was unaffected by DRB (Figs. 3C and 4C). In addition, the colocalization frequency of APH-induced γH2AX with FANCD2 was unaffected by DRB (Fig. 5G, mid and late-S phases).

(b) Non-CFS-L and non-CFS-M RT-delayed breaks were distinct from each other and also from CFS-L and CFS-M breaks (see #2a above). That is, DRB treatment increased and decreased APH-induced γH2AX signal intensity at non-CFS-L and non-CFS-M RT-delayed breaks, respectively (Figs. 3D and 4B).

(c) APH-induced γH2AX signals at non-CFS-E RT-advanced break sites decreased by DRB (Fig. 5C). In addition, the colocalization frequency of APH-induced γH2AX with FANCD2 was decreased by DRB (Fig. 5G, early-S phase).

These data strongly suggest that non-CFS-L and non-CFS-M RT-delayed breaks represent novel classes of chromosome breaks that form in a manner distinct from CFS-L and CFS-M breaks, as their response to the transcription inhibitor DRB was clearly different. We also showed that non-CFS-E RT-advanced break formation is dependent on transcription, suggesting that they could indeed be due to transcription-replication conflicts (TRCs) (we thank Reviewer #3 for the valuable suggestion). While we fully acknowledge that additional experiments will be required to elucidate the precise mechanisms underlying break formation across different classes, we view this as important downstream work to be pursued by the field as a whole, including our own ongoing and future efforts. We described these results in the main text (on pages 8–9,11,14-15, lines 240–263,308–315,419–439) and in Methods (on pages 27–29, lines 799–868).

In addition, we emphasize the following two points:

**(3) Our RT profiling method is superior to those employed in prior CFS studies**

We found that the conventional early and late S-phase BrdU-IP RT profiling method, which has been used in almost all studies of CFSs to date, is not suitable for analyzing APH-induced RT delays in late S-phase. By contrast, our 10-cell Repli-seq pseudobulk RT profiling approach avoids this technical limitation associated with the conventional RT profiling method. For details, please refer to our response to Minor Comment 9 of Reviewer 2.

**(4) We have changed the title**

We changed our manuscript title to "**Single-cell mapping of chromosome breaks identifies multiple fragile site classes with distinct DNA replication timing landscapes"** to emphasize our findings regarding the informative value of RT landscape in untreated cells for replication behavior and propensity for break formation upon APH treatment.

Only the major changes are described above. We did not cover all the revision results in this section. For more details, please refer to the point-by-point response to the reviewers' comments below.

**Reviewer #1**

**Remarks to the Author:**

This manuscript maps aphidicolin-induced fragile sites in single mammalian cells. Previous studies have mapped fragile sites induced by aphidicolin in human and mouse cells, as well as fragile sites induced by driving entry of cells into S phase or in cells that are deficient for homologous recombination. The previous studies have studied populations of cells, meaning that rare fragile sites could not be identified. One exception is the study by Shaikh et al (Genome Biology 2022 = ref. 12 of this manuscript), which examined fragile sites in single cells using the same method as the one employed in the current manuscript. Shaikh et al studied non-transformed cells (RPE1 and BJ) and identified fragile sites, which were mostly associated with transcribed large genes.

In the current manuscript the authors focus on U2OS cells, but also study RPE1 and mouse ESCs. The analysis is more detailed than that performed by Shaikh et al and reveals, according to the authors, 5 types of fragile sites.

Types 1 and 2 are classical common fragile sites (CFSs) mapping to mid-S and late-S replicating regions. Mechanistically they are very similar. They could be even considered as one type.

Types 3 and 4 are not well-defined mechanistically. They map to mid-S and late-S replicating regions. They were not recurrent (unlike types 1 and 2), but it remains possible that fragility at these sites is due to the same mechanism as that leading to fragility at sites of types 1 and 2. This is perhaps the part of the manuscript that deserves greatest attention by the authors. To define too many classes of sites, when these are not very well defined is not helpful.

We thank the reviewer for raising an important point. To address whether non-CFS-L and non-CFS-M represent novel and mechanistically distinct break categories, we performed new experiments and

analyses as explained in the **Summary and Highlights** section (#1a and #2) at the beginning of this response letter. Briefly, we performed experiments to check the dependence of different break classes on transcription. The results are consistent with the idea that non-CFS-L and non-CFS-M RT-delayed breaks represent novel categories that form in a manner mechanistically distinct from each other and also from CFS break classes. This is all described in the main text (on pages 8–9,11,14-15, lines 240–263,308–315,419–439) and in Methods (on pages 27–29, lines 799–868).

We agree that defining too many classes is not necessarily the most effective approach. However, our results suggest that there are fragile site classes that have been overlooked, which is likely due to the lack of single-cell profiling studies (except for the study by Shaikh et al., *Genome Biol*, 2022). Reducing the existing categorization will potentially mask the 'rare' break classes and their unique properties identified in our single-cell study. As such, while we admit that we have a long way to go in defining the exact mechanisms, we strongly believe it is important to report the presence of previously unappreciated forms of chromosome fragility.

Type 5 sites are sites of transcription-replication conflicts. They tend to map to early-S replicating regions. I wonder if they overlap with the oncogene-induced regions defined by Macheret et al (Nature 2018).

Thank you for your valuable suggestion. Compared to randomized control with similar RT, non-CFS-E RT-advanced breaks were enriched for origins in normal condition, but not for oncogene-induced origins (Fig. 5B; Supplementary Fig. 7C). The results are discussed on page 14, lines 403–407.

Overall, I am not sure that the current study provides novel mechanistic insights on how fragile sites are generated in response to aphidicolin, but the study is carefully performed and the data will be useful to the community. Thus, I would recommend publication after some analysis reveals whether types 3 and 4 are distinct from types 1, 2 or 5. Regarding the publication venue, I would not make a strong case for Nature Communications versus a Genomics journal.

Thank you very much for acknowledging the quality of the work. We believe our study will serve as an important starting point for investigating chromosomal vulnerability beyond the CFS or other recurrent break sites. Although we have added new data to suggest that the novel break types we identified are mechanistically distinct from the CFS break classes, we realize that deeper mechanistic understanding is still lacking. However, we also think that a thorough mechanistic study is beyond the scope of this study, especially at this early stage, when little is known beyond the recurrent CFS breaks. Our data revealed the frequency, RT/compartment landscape, and features of different classes of breaks, thereby providing the first holistic view of sequences prone to breaks under replication stress. In addition, we have now added experiments to show that mechanistic differences exist among non-CFS-L, non-CFS-M, and CFS break classes (please refer to our response to your first comment above). We hope we have addressed the reviewer's concerns.

**Reviewer #2**
**Remarks to the Author:**
In this work, Elumalai and Hiratani performed a study to map and classify aphidicolin-induced chromosome breaks identified by single-cell Whole-Genome Sequencing (scWGS) in human (U2OS cells) and mouse (CBMS1 mESC) cell lines. Based on the replication timing (RT) data (early, mid, or late) and previously published aphidicolin-induced MiDAS-seq signals (or G2/M-seq data generated in the current study) that enrich within common fragile sites (CFSs), they classified chromosomal break regions into five classes. Two classes (Class-1 and Class-2), with recurrent breaks and mid or late RT, are associated with CFSs, and three other classes are not enriched with MiDAS-seq signals. The authors further perform additional bioinformatics analyses to try to identify the genomic and epigenomic features (including 3D genome organization) of different classes of breaks. They concluded that Class-4 is a novel class of breaks that coincided with RT transition regions (TTRs) and A/B compartment boundaries,

and Class-5 is another class that showed early RT and enrichment for R-loops and G quadruplexes that might be associated with transcription-replication conflicts. In addition, the authors re-analyzed the previously published data of aphidicolin-induced chromosome breaks mapped by scWGS in RPE1 epithelial cells and BJ fibroblast cells to show that these 5 classes of breaks also exist in other human cell lines.

While the topic aligns with an important area in the field of genome instability, the current manuscript falls short of the journal's standards for novelty, rigor, and mechanistic insights. Several of the findings described are either overlapping with or lack sufficient differentiation from previous seminal works, such as Sarni et al. (Nat. Commun., 2020) and Shaikh et al. (Genome Biol., 2022). Moreover, key conclusions, such as the identification of novel classes of breaks (e.g., Class-4 and Class-5), appear inadequately supported by the presented analyses (see below for detailed comments). The study also misses opportunities to leverage its dataset to address critical mechanistic questions in the field. Therefore, the current study is largely premature, and substantial revisions are required before this manuscript can be considered for publication.

Thank you very much for the thorough and accurate summary of our manuscript. We acknowledge that certain aspects of our study overlap with the work of Shaikh et al., *Genome Biol*, 2022, and indeed, we have utilized some of their data in our analysis. However, their study primarily focused on CFS-type recurrent breaks near long genes that exhibit mid/late-S RT and delayed RT upon aphidicolin (APH) treatment, which corresponded to our CFS-L and CFS-M breaks. In contrast, our study provides a more comprehensive view of genomic sequences susceptible to breakage under replication stress, extending beyond CFS break classes.

Sarni et al.'s (*Nat Commun*, 2020) work is also a well-executed study that we read with great interest. However, as we will describe below, it appears that Reviewer 2 has overlooked the fact that Sarni et al. did not comprehensively identify break sites using single-cell sequencing. Instead, they relied on the classical cytogenetics-based APH-induced CFS sites they had originally reported in 2015. There is, of course, nothing wrong with this approach; the Sarni et al. paper is, in our view, an important and valuable paper in its own right. Nonetheless, it should be emphasized that their work is specifically a study of CFSs and therefore has little relevance to the non-recurrent break sites identified in our study. We will elaborate on this point in our response below.

**Major comments:**

1. **Novelty and Contextualization**

   The findings presented here overlap extensively with prior publications, particularly Sarni et al. (Nat. Commun., 2020) and Shaikh et al. (Genome Biol., 2022). For example:

   a) The classification of chromosome breaks based on RT and genomic features is not new. Sarni et al. already established such classifications, which align closely with the classes described here. In study by Sarni et al., they classified aphidicolin-induced RT-variable regions into 5 different classes (called "cluster" or "signature") based on RT data (early, mid, or late) and the stage of alternation (i.e. advanced or delayed RT in aphidicolin-treated cells). The Class-1 and 2 identified in the current study are likely the Cluster/Signature 3 and 4 identified by Sarni et al. (mid/late regions associated with CFSs with delayed RT), and the Cluster/Signature 5 identified by Sarni et al. is similar to the Class-5 in this study (early region with advanced RT).

It appears that the reviewer may have misunderstood the scope and analytical focus of the Sarni et al. study. Let us clarify.

As can be seen from the first few sections of the Sarni et al. (*Nat Commun*, 2020) study, the authors did not classify chromosome breaks based on RT. Instead, they analyzed genome-wide RT profiles before and after APH treatment (Fig. 1 of Sarni et al.), identified RT-switching regions (which accounted for ~4% of the genome), and grouped these regions into five clusters. Thus, Sarni et al. classified RT-switching regions, rather than chromosome breaks, whereas our study directly classifies chromosome

break sites. Because these classifications are applied to fundamentally different entities, a direct comparison between the two studies is not appropriate.

In addition, Sarni et al. focused on 24 recurrent APH-induced CFS chromosomal bands that had been identified by classical cytogenetics in their previous study (Miron et al., *Nat Commun*, 2015). As a result, their analysis was restricted to recurrent CFSs, and, importantly, their resolution was at the megabase scale, as cytogenetic bands represent broad genomic intervals rather than precise genomic coordinates. They then compared these cytogenetically defined CFS bands with RT data and reported that 20 out of the 24 loci overlapped with APH-induced RT-delayed regions; however, they also noted that 7 of those 20 loci exhibited RT advancement (Fig. 1f of Sarni et al.). This apparent overlap is a natural consequence of the low-resolution approach, in which large cytogenetic bands can encompass both RT-delayed and RT-advanced subregions within the same interval. For two CFS bands (2q22 and 22q12), break positions were further refined using DNA-FISH analysis.

It is possible that the RT profiles associated with our CFS and non-CFS-E break classes partially overlap with the RT-delayed and RT-advanced regions reported by Sarni et al., respectively. However, this does not undermine our conclusions. As emphasized above, Sarni et al.'s RT clusters represent classifications of chromosomal domains based on changes in RT induced by APH treatment, whereas our break classes are defined by actual chromosome break positions identified from single-cell data, providing substantially higher resolution and coverage. Consequently, the two datasets and their respective classification schemes describe fundamentally different entities.

We would like to reiterate our appreciation for the important contributions of Sarni et al. However, while the two studies may appear similar at first glance, their scientific focus, experimental resolution, and analytical objectives are, in fact, quite distinct.

b) The association of Class-4 breaks with RT transition regions and A/B compartment boundaries is a well-documented feature of CFSs.

To the best of our knowledge, no study has reported replication timing transition regions (TTRs) as a hallmark of CFSs. In support of our statement, a new category of fragile sites, TTR fragile sites (TTRFSs), was only recently reported (Corrazi et al., *Nat Commun*, 2024) and these authors defined them as distinct from CFSs. However, when we compared the reported TTRFSs with our non-CFS-M (TTR) breaks, we found that 1/43 TTRFSs overlapped with our breaks (Supplementary Fig. 11C). In addition, TTRFS were frequently located near long genes (>= 300 Kb) and often corresponded to regions replicated by converging replication forks, both of which are characteristic features of CFSs (former Supplementary Fig. 8; now Supplementary Fig. 11A,B). Therefore, it is likely that many of the TTRFSs could actually correspond to CFS break classes in our definition, rather than non-CFS-M RT-delayed breaks, which frequently correspond to uni-directional TTRs. We described these results on page 22, lines 651–660.

c) Class-5's enrichment for transcription-replication conflict markers, such as R-loops and G quadruplexes, reflects known characteristics of early RT regions rather than a novel mechanistic insight.

We did not intend to suggest that this represents a novel mechanistic insight. Rather, our goal was to test the possibility that a subset of non-CFS-E break sites (i.e., non-CFS-E RT-advanced breaks) were frequently associated with transcription-replication conflicts (TRCs) compared to RT-matched controls. We confirmed this association by demonstrating the significant enrichment of these sites for APH-induced FANCD2 (known to resolve TRCs), R-loops (known to mark TRCs), G-quadruplex (G4) repeats (linked to R-loops), and replication initiation sites, compared to randomized controls with similar RT (former Fig. 4A; now Fig. 5B).

Because early-replicating fragile sites (ERFSs), oncogene-induced origins, and atypical-MiDAS regions

have all been reported to replicate in early S phase and to arise from TRCs, we also compared our non-CFS-E break regions to these datasets. Interestingly, non-CFS-E break regions were enriched only for ERFSs but not for oncogene-induced origins or atypical-MiDAS regions (Supplementary Fig. 7B–D). These observations strongly suggest that the genomic features associated with non-CFS-E break regions cannot be explained simply by their association with early-S RT. Instead, our correlative analyses indicate a more complex relationship with multiple genomic features characteristic of early RT regions. We described these points on pages 12–14, lines 360–417.

The manuscript fails to provide compelling evidence that these findings represent significant advancements beyond what is already known.

We respectfully disagree with this assessment, as it does not fully reflect the novelty and breadth of our work. As noted in our response to Comment 1a, the reviewer's comparison of our study with that of Sarni et al. is not appropriate. With regard to Shaikh et al. (*Genome Biol*, 2022), we fully acknowledge that they were the first to profile fragile sites in single cells using a similar approach, and we greatly appreciate their important contribution. However, their study primarily focused on recurrent CFS-type breaks near long genes with mid/late-S RT and delayed RT upon APH treatment, corresponding to our CFS-L and CFS-M breaks.

In contrast, our study identifies and characterizes additional classes of breaks that extend beyond the canonical CFS-type categories. This provides, for the first time, a comprehensive view of genomic regions prone to breakage under replication stress, as well as their relative frequencies within a single experimental framework. Moreover, we show that the RT/compartment landscape in the untreated condition can predict the replication behavior and break propensity induced by APH treatment. Taken together, our findings establish an important foundation for future investigations into chromosomal fragility beyond CFSs and other recurrent break sites.

2. **Insufficient Categorization of Chromosome Breaks**
   It has been well-documented that different replication stresses can generate different types of copy number alterations (CNAs) at different genomic contexts. Notably, in the study by Shaikh et al., separating CNAs identified by scWGSs into different categories allowed them to show that low doses of aphidicolin treatment favor large CN losses with recurrent breaks associated with cell type-specific CFSs. The analysis in the current study lacks granularity, as all chromosome breaks are grouped together without distinguishing break types (e.g., gains vs. losses, small vs. large CNAs). This approach obscures potential mechanistic differences and makes it difficult to contextualize the findings relative to prior studies. Without a more refined categorization, the results offer limited interpretative value. As already pointed out by the authors in the current manuscript, "Recently, using scRepli-seq technology, large (>20 Mb) and small (<20 Mb) replication stress-induced CNVs were identified in single mammalian cells (Ref 12). However, only large CNVs were found to display features of CFSs, suggesting there could be other yet-to-be defined categories of fragile sites". However, it is unclear how, without performing any distinction of breakpoints as did in the current manuscript, the authors can better address this question comparing the previous study.

We thank the reviewer for this important comment. To compare our study with Shaikh et al. (*Genome Biol*, 2022), the types (gains or losses) and the sizes of CNVs surrounding APH-induced breaks from U2OS, hTERT-RPE1, BJ-hTERT, and CBMS1 mESCs were determined. We did not observe an enrichment of losses or gains for any of our break classes in all the cell lines analyzed (Supplementary Fig. 4E-H; Supplementary Table 4 for statistics). Interestingly, the median sizes of both losses and gains associated with CFS breaks, especially CFS-L, were frequently larger compared to those of non-CFS breaks with similar RT (Supplementary Fig. 4A-D). However, due to the limited number of APH-induced breaks in some cell lines/break classes, statistical analysis remains uncertain. Nevertheless, this observation was consistent with Shaikh et al., suggesting that large CNVs are associated with CFSs. Furthermore, this highlights the uniqueness of our study, as we focused on the non-CFS break classes that are associated with smaller CNVs. We described these points on pages 7,18,19, lines 201–204,542–

548,570–572.

3. **Insufficient Evidence for Key Claims**
   Several major conclusions, including the identification of Class-4 and Class-5 as distinct categories, are inadequately supported:

   a) Class-4 breaks likely represent extensions of CFS regions rather than a novel break type. This interpretation aligns more closely with prior literature, as features such as delayed RT and association with TTRs are hallmarks of CFSs. Based on Figure 2F, Class-4 showed similar transcriptional activity as Class-2 with no significant difference. Based on Figure 3C, the RT slope of Class-4 (with delayed RT) is also similar to that of Class-2. Importantly, Figure 3F shows that the Class-4 breaks are located at the extremities of CFSs (based on the MiDAS signals). Therefore, it is more likely that Class-4 and Class-1/Class-2 breaks indeed result from the same phenomenon, i.e. the breaks associated with CFSs, which are large mid- to late-replicating regions poor in replication initiation induced by large active genes. The difference is that Class-1/Class-2 correspond to the core regions of CFSs (frequently around the gene centers), which show strong MiDAS signals (population average) and recurrent breaks. While the under-replicated regions within CFSs in aphidicolin-treated cells can display certain degrees of variation in the cell population, they can also extend outside of these CFS core regions (e.g. closer to gene extremities) and induce chromosome breaks, although with lower frequency. This can explain all the observed results shown in the current study. In agreement, Figure 3D shows that Class-4 (delayed RT) breaks show higher MiDAS signals than random control, although at a level much lower than that of Class-2. Additionally, Sarni et al. (Nat. Commun. 2020) have shown that an important feature of CFSs is comprised of a TAD boundary (frequently at gene extremities) overlapping a highly transcribed large gene with aphidicolin-induced RT-delay. Considering that TTRs are frequently associated with transition regions between A/B compartments, it is not surprising that the authors find an association of Class-4 breaks (frequently located at extremities of large active genes poor in replication initiation, therefore, TTRs) coinciding with A/B compartment boundaries.

Once again, we would like to re-emphasize that TTRs have never been reported as hallmarks of CFSs in the literature. Please refer to our response to Major Comment 1b for additional details. Contrary to the reviewer's interpretation, non-CFS-M breaks showed transcriptional activity similar to RT-matched random controls, whereas CFS-M showed higher transcriptional activity (Fig. 2F). In contrast to CFS-M, non-CFS-M RT-delayed break regions exhibited RT slopes that were significantly steeper than those of randomized controls with similar RT (Supplementary Fig. 6C). Furthermore, CFS-M, but not non-CFS-M breaks, showed a much greater degree of APH-induced RT delay (former Fig. 3B; now Fig. 4D). In addition, compared with CFS-M, non-CFS-M breaks were located much farther from long genes (former Fig. 2D; now Fig. 2B), displayed distinct RT and A/B compartment landscapes (former Fig. 3B; now Fig. 4D), were associated with smaller CNVs (Supplementary Fig. 4A; see Median size), and were not enriched for APH-induced FANCD2 (former Fig. 4A; now Fig. 5B).

The reviewer stated that "*it is more likely that Class-4 and Class-1/Class-2 breaks indeed result from the same phenomenon, i.e. the breaks associated with CFSs, which are large mid- to late-replicating regions poor in replication initiation induced by large active genes*." However, based on the series of distinct genomic features discussed in the previous paragraph—which clearly distinguish non-CFS-M (class-4) from CFS-M (class-2) breaks—we believe this is not the case.

The reviewer's next statement (in *italics* below) is also highly speculative and lacks supporting evidence. We did not find any indication that non-CFS-M breaks occur near CFS regions (Fig. 2E). Therefore, this explanation cannot account for the observations reported in our study:
*"While the under-replicated regions within CFSs in aphidicolin-treated cells can display certain degrees of variation in the cell population, they can also extend outside of these CFS core regions (e.g. closer to gene extremities) and induce chromosome breaks, although with lower frequency. This can explain all the observed results shown in the current study."*

We must also note that the reviewer's subsequent statement is not statistically supported (former Fig. 3D; now Supplementary Fig. 6D, p=0.443):
*"In agreement, Figure 3D shows that Class-4 (delayed RT) breaks show higher MiDAS signals than random control, although at a level much lower than that of Class-2."*

Furthermore, the reviewer's following comment is not consistent with the available evidence:
"*Additionally, Sarni et al. (Nat. Commun. 2020) have shown that an important feature of CFSs is comprised of a TAD boundary (frequently at gene extremities) overlapping a highly transcribed large gene with aphidicolin-induced RT-delay. Considering that TTRs are frequently associated with transition regions between A/B compartments, it is not surprising that the authors find an association of Class-4 breaks (frequently located at extremities of large active genes poor in replication initiation, therefore, TTRs) coinciding with A/B compartment boundaries."*
While Sarni et al. indeed demonstrated that some CFSs coincide with TAD boundaries and highly transcribed large genes, several key facts must be considered: (1) our non-CFS-M RT-delayed breaks showed transcriptional activity similar to RT-matched randomized control (Fig. 2F); (2) only a subset of TAD boundaries coincide with A/B compartment boundaries; and (3) we have now shown that non-CFS-M breaks were not closer to CFSs (Fig. 2E) and TAD boundaries (Supplementary Fig. 5C), compared to RT-matched random controls. Therefore, we do not see how our observations regarding non-CFS-M breaks could be considered expected or unsurprising.

Results showing distance to TAD boundaries and CFSs are explained on pages 8,54, lines 214–220,1540–1555.

b) The resolution of scWGS (500 kb) limits the ability to attribute transcription-replication conflict features specifically to Class-5 breaks. The observed associations appear to be driven by replication timing rather than a distinct mechanism. It is well known that both R-loops and G quadruplexes are enriched at early-replicating regions, Therefore, it is not surprising that the authors find an enrichment of these features at Class-5 breaks and not others. Although the authors showed a difference between the Advanced-RT and Delayed-RT groups of Class-5 breaks, it lacks a propre control analysis to evaluate the significance and interpretation of such difference. Based on Figure 4A, it is clear that the enrichment of R-loops and G quadruplexes is tightly linked to replication timing (Class-5 > Class-2/4 > Class-1/3). If Advanced-RT Class-5 breaks have an earlier RT than the Delayed-RT Class-5 ones, it can well explain the observed results shown in Figure 4A. The authors need to evaluate/analyze the RT distribution of each sub-group for comparison.

The resolution of our breaks is higher than 500 kb, as we used the 95% confidence interval to further narrow down the break site positions (median size: 270 kb). Regarding R-loops and G-quadruplexes, while we agree that these features are generally enriched in early RT regions, it is noteworthy that only non-CFS-E RT-advanced breaks regions, but not RT-delayed or RT-matched randomized control regions, showed an enrichment of R-loops and G-quadruplexes. We kindly remind the reviewer that we used randomized control regions with similar RT profiles to validate this conclusion. Please refer to our response to Major Comment 1c for additional details. In addition, we did not observe any difference in the untreated RT value distribution between non-CFS-E RT-advanced and RT-delayed breaks (Fig. 2C, 5B).

**4. Methodological and Statistical Rigor**

a) The manuscript relies heavily on descriptive observations and lacks the statistical rigor required to substantiate its claims. Key analyses (e.g., enrichment of R-loops and G quadruplexes in Class-5) do not include appropriate controls or statistical tests to evaluate significance. Randomization or permutation tests taking into account the RT distribution of each subgroup (to get a P-value to show that whether the observed Z-score is significant or not) would be necessary to rule out artifacts.

We kindly remind the reviewer that we used randomized control regions with similar RT to support our conclusion. Furthermore, as requested, we have now assessed the statistical significance and reported the corresponding p-values for the Z-score heatmaps in Supplementary Table 4.

b) Similar criticism applies to the results showed in Figure 5. Please provide the RT distribution for each subgroup. If in each RT fraction (i.e. from S2 to S9) the Advance-RT group has the earliest RT while the Delayed-RT group the latest RT, this can explain the results shown in Figure 5C, as all these features show higher signals at the earlier RT regions. Statistical evaluations and P-values, therefore, need to be provided for the figures.

We thank the reviewer for this comment. We have now added the mean pseudobulk RT values for each subgroup in each untreated HMM fraction (former Fig. 5C; now Fig. 6F). As pointed out by the reviewer, in each HMM, APH-induced RT-advanced and RT-delayed regions have earlier and later RT, respectively, in both untreated and APH-treated conditions (Fig. 6F; See supplementary table 4 for statistics). However, it was unclear why genomic regions that deviate from their mean untreated RT (earlier or later replicating) and other genomic/epigenomic features tend to further change their RT upon APH treatment, but this was also interesting to us. We aimed to convey that APH-induced RT-changing regions can be predicted solely by examining various genomic/epigenomic features in untreated conditions. We have rewritten the main text to make this clearer. Please refer to page 17, lines 496–508.

PS: it seems that the percentage of Advanced and Delayed RT regions are very high in each RT fraction (comparing to those reported by Sarni et al., 2020). I wonder whether this is due to the authors not setting any cutoff to define the Advanced and Delayed RT regions (Page 11, "we identified genomic regions that shifted fractions (either earlier or later) following aphidicolin treatment as advanced or delayed RT regions"). This might capture stochastic variations in RT data between the two conditions. If the authors restricted their analyses to regions with significant RT swifts, would they observe similar or different results?

By setting a global threshold (> |2*SD|), we found that, similar to Sarni et al., 5% of the genome changed their RT upon APH treatment (Supplementary Fig. 8B). Interestingly, similar to 10-HMM RT analysis, APH-induced advanced and delayed RT regions at early/mid/late-S fractions deviated from their mean genomic and epigenomic features in untreated conditions (Supplementary Fig. 8C). Furthermore, when 10-HMM RT was compared to 5-HMM RT analysis, we found that the types and locations of RT-changing regions were largely conserved between the two settings, suggesting that the RT changes identified by 10-HMM RT analysis are not stochastic variations in RT (Supplementary Fig. 8A). We described these points on pages 17,54–55 lines 491–494,1557–1573.

c) In Figure 6, the authors show the numbers and percentage of breaks in 5 Classes in RPE-1 cells (B,C), BJ cells (D,E) and mESC cells (G). However, without a control analysis, it is hard to evaluate whether the obtained results signify some biological meaning. The authors need to generate random simulation data with similar RT distribution to check whether the observed results are significantly different from random expectation. This is particularly important considering that the CFSs of U2OS cells were used in the figure, and we know that CFSs are cell type specific.

We thank the reviewer for the thoughtful comment. To assess whether the classification of breaks into RT–MiDAS categories reflects biologically meaningful patterns rather than random distribution, we performed a permutation-based control analysis. Specifically, for each cell type (U2OS, RPE1, BJ), we generated 1,000 sets of randomly located genomic loci, matched in number to the observed breaks, and classified each into the same RT–MiDAS categories, using the same pseudobulk RT and MiDAS-seq profiles as those used in the original analysis.

We then compared the observed frequency of breaks in each class to the distribution obtained from the randomized controls. This revealed that break frequency is significantly different from randomized

control frequency in all the cell lines analyzed. Specifically, recurrent CFS-L breaks were more frequent than expected by chance (Supplementary Fig. 3F and Fig. 7F). These results strongly suggest that APH-induced breaks are non-randomly distributed on the genome. We described these points on pages 7,18, lines 186–191,538–542.

**5. Limited Mechanistic Insights**
The study largely recapitulates known features of aphidicolin-induced replication stress without addressing critical unresolved questions in the field. For example:
What mechanisms drive the formation of specific break classes? For example, comparing data obtained in mutant cells (as in Shaikh et al., 2022) to support the role of a gene/pathway in the formation of specific break classes.
• How do these findings advance our understanding of replication stress-induced fragility beyond previous work?
The lack of mechanistic depth significantly undermines the manuscript's potential impact.

We agree that Shaikh et al. (*Genome Biol*, 2022) were the first to profile fragile sites in single cells using a similar method, and we greatly appreciate their pioneering work. However, their study primarily focused on recurrent CFS-type breaks near long genes exhibiting late-S RT and delayed RT upon APH treatment, which correspond to our CFS-L and CFS-M break classes. In contrast, our study examined the frequency, RT/compartment landscape, and molecular characteristics of multiple break classes including non-recurrent breaks, thereby providing the first comprehensive view of genomic regions prone to breakage under replication stress in a single experimental framework.

While we acknowledge the limited mechanistic insight, we have now added new experiments to investigate the mechanistic differences among break classes, which are described in the Summary and Highlights section (#2) at the beginning of this response letter. The results are consistent with the idea that non-CFS-L and non-CFS-M RT-delayed breaks are mechanistically distinct from each other and also from CFS break classes. In addition, we could validate that non-CFS-E RT-advanced breaks were dependent on transcription. We argue that these new data are sufficient to claim that the different classes of breaks we identified are generated by distinct mechanisms. While we fully acknowledge that additional experiments will be required to elucidate the precise mechanisms underlying break formation across different classes, we view this as important downstream work to be pursued by the field as a whole, including our own ongoing and future efforts. We hope we have addressed the reviewer's concerns. This is all described in the main text (on pages 8–9,11,14-15, lines 240–263,308–315,419–439) and in Methods (on pages 27–29, lines 799–868).

**Minor comments:**
- 1. "To determine the cell-cycle duration of U2OS cells, cells were released from a double thymidine block with or without low-dose aphidicolin,"
*Please indicate, in the main text, the concentration of aphidicolin used in the current study to avoid requiring readers to check the figure or method.*

Done (page 3, lines 68–69).

- 2. Page 3, "Based on the 2D cell-cycle FACS, single G1 cells were sorted and scRepli-seq was performed as described16 (Fig. 1A, Experiment)."
*Based on the experiment protocol, it's indeed a classical scWGS of G1 cells (exactly the same principle as Shaikh et al. 2022). Therefore, please modify the description of this section and the corresponding figures to avoid any confusion. scRepli-seq allows obtaining information on DNA replication timing, while here the scWGS data identify scCNAs and will not provide information on RT.*

As the reviewer correctly pointed out, scRepli-seq is, in principle, a scWGS method to detect DNA copy number. However, many scWGS methods exist. Since we precisely followed our homemade scRepli-seq

protocol (Takahashi et al, *Nat Genet* 2019; Miura et al., *Nat Protoc* 2020), we would like to use the same nomenclature to avoid unnecessary confusion. For better understanding, we have edited "scRepli-seq" to "scRepli-seq for karyotyping" on pages 2–3, lines 54–55,80.

- 3. Page 4, "karyotype heterogeneity increased as judged by visual inspection (Fig. 1B, compare top and bottom panels), which was statistically confirmed by analyzing the degree of cell-to-cell karyotype heterogeneity (Fig. 1C)."
*Please provide the P-values and the corresponding test to support the statement of "statically confirmed".*

We have already calculated the karyotype heterogeneity score and verified its statistical significance. Please see Fig. 1c. Details of the measurement of karyotype heterogeneity scores are described in the Methods section (page 25, lines 749–754).

- 4. Page 4, "We then refined the breakpoint position to be close to the actual breakpoint position using uniquely mapped reads and calculated a 95% confidence interval (CI) for each refined chromosome breakpoint, which we defined as a chromosome break region (or break region for short) (Fig. 1A, Data analysis)".
*It is unclear how this process was performed. Please provide more details in the Methods section. What is the resolution/precision of the refined breakpoint positions? Is it much better than 500-Kb (the bin size used for the breakpoint identification)?*

We have added more details on how we identified the chromosome break regions in the Methods section (page 25, lines 756–765). Since breakpoints need not necessarily be at the 500-kb bin junctions, we refined their positions using mapped reads and calculated the 95% confidence interval (CI) around each breakpoint. We refer to this range (95% CI) as chromosome break regions, which vary in size and have a median size of 0.27 Mb.

- 5. Page 5, "Aphidicolin-induced break regions had a median size of 0.27 Mb and 76% of the break regions were less than 0.5 Mb in size (Supplementary Fig. 1C)".
*If I understand correctly, this is the size of regions enriched with aphidicolin-induced specific breakpoints, and not the size of CNAs associated with aphidicolin-induced breaks. Therefore, it might better to avoid using "regions" to avoid confusion.*

Yes, your understanding is correct. As mentioned in minor comment #4, breaks are not a single base pair, but a 95% confidence interval (CI) around single bp breakpoints. While we used "breaks" for short, in some places it was necessary to use "break regions" to make sure it is not misleading. For your information, we defined them on pages 4,25, lines 99,761–762 .

- 6. Instead of focusing directly on Aphidicolin-induced breaks, the authors should first characterize and compare breaks identified in non-treated cells versus Aphidicolin-treated cells. For instance, details on the type and frequency of CNAs (gains vs. losses), their size distribution, and their RT distribution would provide a clearer context for understanding features specific for aphidicolin-induced breaks.

We respectfully disagree with the reviewer's suggestion. We prefer to maintain our focus on APH-induced breaks because U2OS cells possess a highly rearranged and complex karyotype, resulting in numerous pre-existing structural variants and breakpoints even under untreated conditions. A comprehensive characterization of these background events is beyond the scope of the present study.

For APH-induced breaks, we have already shown their distribution across the RT–MiDAS categories and the frequency of each break class. In addition, we have now included analyses of the types and sizes of CNVs associated with each break class. Please refer to our response to Major Comment 2 for further

details.

- 7. Page 4, "We found that 38% (103/274) of all break regions corresponded to CFSs, which was statistically significant (p = 0.001; vs. 1000 randomized control regions)."
*Did the generation of randomized control regions take into account the RT distribution as in some other randomized control analyses? If not, is this result still signification when considering RT distribution of the break regions?*

No, we did not take RT distribution into account in this analysis, because the breaks had not yet been categorized and RT had not been introduced at that point in the manuscript. However, our new analysis performed in response to your Major Comment 4c indicates that CFS break classes, specifically CFS-L, occur at a higher frequency than randomized controls within the same RT–MiDAS category (Supplementary Fig. 3F and Supplementary Table 4).

- 8. Page 4. "Even for this comparison, 39% (107/274) of our chromosome break regions corresponded to CFS break regions, indicating that our experimental and analytical setup using scRepli-seq safely captured known CFSs".
*What about the numbers and percentages of CFS regions (e.g. identified by MiDAS-seq) associated with chromosome breaks identified in the current study? Please provide and interpretate this analysis.*

We thank the reviewer for this comment. When the 324 CFSs identified by MiDAS-seq were compared with our break regions, we found that 16% (53/324) of all CFSs contained at least one break region (Supplementary Fig. 1F). Conversely, when starting from our 274 break regions, 39% (107/274) overlapped with MiDAS-seq-defined CFSs. This asymmetry arises because the relationship is not one-to-one: multiple break regions can fall within the boundaries of a single MiDAS-seq–defined CFS. We have now described this in the main text on page 4, lines 114–115.

- 9. Page 5. "The 10-cell Repli-seq log2repliscore RT values of all 14 S-phase fractions were averaged to obtain a 'pseudobulk' genome-wide RT profile (Fig. 1E, Supplementary Table 2),".
*If only pseudobulk RT data were used in this analysis, what additional information does it provide compared to population-based RT data (e.g. classical Repli-seq)? It seems that scRT data, generated using a more complicated protocol, was reduced to pseudobulk RT data in the final analysis. Does this imply scRT data added no advantages?*

We sincerely thank the reviewer for this important comment.

Unexpectedly, we noticed that APH-induced RT-delayed regions determined by pseudobulk RT comparison (APH – untreated) frequently corresponded to RT-advanced regions based on BrdU-IP RT comparison (Supplementary Fig. 3A, within dotted lines). Such discrepancy was frequently observed at the 324 CFSs identified as sites of G2/M DNA synthesis by APH-induced MiDAS-seq, especially late-S replicating CFSs (Supplementary Fig. 3B). APH-induced RT advance at CFSs was also reported recently using the conventional two-fraction BrdU-IP Repli-seq (Kodali et al., *Front Genet*, 2022). This was puzzling, as CFSs are known to remain under-replicated at the end of S-phase and undergo continued DNA synthesis in G2/M. Consistent with this, CFSs had fewer BrdU-IP reads in late S-phase of APH-treated cells compared to untreated cells (Supplementary Fig. 3C). This could bias the early-versus-late BrdU-IP ratio and lead to an artificial RT advance rather than a true biological advance. To test this possibility, we performed BrdU-IP of the very-late-S/G2/M fraction of U2OS cells treated with low-dose APH (Supplementary Fig. 3D). Consistent with the pseudobulk RT profile, CFSs frequently showed increased BrdU incorporation in the very-late-S/G2/M fraction upon APH treatment (Supplementary Fig. 3C,E), indicating that CFSs actually delay their RT, remain under-replicated at the end of S-phase, and continue to replicate during G2/M. This finding highlights the technical limitations of the early and late S BrdU-IP RT profiling method, as well as the superior temporal resolution of the 10-cell Repli-seq pseudobulk RT profiling approach.

As such, there is actually a huge advantage in using pseudobulk RT analysis based on 10-cell Repli-seq data, which avoids the issues with conventional RT analysis based on early vs. late-S Repli-seq (which was a method also employed in the Sarni et al. 2020 paper). For these reasons, we used 10-cell Repli-seq's pseudobulk RT and avoided the use of the conventional two-fraction early vs. late S-phase BrdU-IP RT profiling method. These points are described in the main text on page 5–6, lines 148–168.

- 10. Page 5, "Class 1, late-S RT CFS (30%); class 2, mid-S RT CFS (12%); class 3, late-S RT non-CFS (9%); class 4, mid-S RT non-CFS (20%); and class 5, early-S RT non-CFS (29%)".
*In addition to percentage, please include the actual count of breaks (n) in each class.*

Done (page 6–7, lines 178–181). Further we have changed the nomenclature of break classes. For more details, please refer to the Summary and Highlights section at the beginning of this response letter

- 11. Fig. 2B, the amount of Advance-RT (~ one third) group in Class-1 is much higher than that of Class-2 (only a few). Any explanation?

We speculate that it could be a limitation of 10-cell Repli-seq. While 10-cell Repli-seq pseudobulk RT is much better than two-fraction BrdU-IP Repli-seq (please refer to our response to minor comment 9 for more details), it is possible that it may have difficulties in identifying RT-delay in the very late-S phase, though not to the same extent as the two-fraction BrdU-IP Repli-seq. We described these points on pages 7,53, lines 206–207,1525–1530.

- 12. Fig. 2E, the numbers of break regions indicated in this figure agree with the numbers shown in Fig. 1G, however, they are different compared to the numbers shown in Fig. 2 (B-D,F-G). Please provide an explanation for these differences and ensure consistency across all figures.
*PS: move Fig. 2E into Fig. 1 since it seems that they belong to the same object.*

Fig. 2E is now moved to Fig. 1H.

Current Fig. 1G,H shows the total break number (including redundant), whereas other plots (former Figs. 2 B-D,F-G; now Figs. 2C,D,B,F, 3E) show the number of non-redundant breaks in each break class. We described these points in the main text on page 4, lines 115–117, and also in the respective figure legend.

- 13. Page 5, "The degree of RT delay is correlated well with aphidicolin-induced MiDAS-seq signal intensity (Supplementary Fig. 3A), suggesting that aphidicolin-induced RT changes can be used to predict CFSs".
*This observation aligns with numerous prior studies (e.g., Sarni et al., 2020). Please rephase it to better appreciate the exiting literatures in the field and avoid suggesting this is a novel finding.*

The fact that CFSs exhibit RT delay has been reported. However, to our knowledge, the correlation between the degree of RT delay and the fragility of CFSs has never been reported. This is particularly important, as our 10-cell Repli-seq pseudobulk RT demonstrated increased temporal resolution compared to BrdU-IP (please refer to our response to minor comment 9 for more details). The new Supplementary Fig. 5B (former Supplementary Fig. 3A) suggests that the degree of RT delay based on 10-cell Repli-seq could be used as a standalone metric to identify CFSs similar to MiDAS-seq. We described these points on page 7–8, lines 210–212.

- 14. Page 6. "we performed a more simple and straightforward experiment, namely BrdU-IP of G2/M-phase cells followed by sequencing, or G2/M-seq, which directly measured BrdU incorporation, i.e., DNA synthesis in G2/M (Supplementary Fig. 3B)".

Please provide additional details on the difference between G2/M-seq and MiDAS-seq. What are the advantage and limitations of each?

For MiDAS-seq, cells were arrested at G1/S with thymidine, released into S-phase with or without low-dose aphidicolin, and again arrested at the G2/M boundary using RO3306 (CDK1 inhibitor). Cells at the G2/M boundary were then released into fresh medium containing EdU to label DNA synthesis in early mitosis in the presence of both hydroxyurea (to prevent S-phase replication) and nocodazole (to arrest cells at prometaphase). Then, mitotic cells were collected using a mitotic shake-off, and EdU-labeled DNA was pulled down and sequenced. The advantage of MiDAS-seq is that the signal-to-noise ratio is high (i.e., it is very sensitive at detecting DNA replication in early mitosis). However, as one can imagine, the experiment suffers from multiple drug treatments and a long duration of RO3306 treatment. Another limitation is that it cannot detect DNA replication in the G2 phase.

On the other hand, our late-S/G2/M-seq involves 30-minute pulse labeling of asynchronous cells with BrdU, collecting the very late-S/G2/M fraction (i.e., the 4C DNA content population) from a simple PI-stained cell-cycle FACS plot, followed by immunoprecipitation of BrdU-labeled DNA and sequencing. The signal-to-noise ratio is somewhat low, as there is a possibility of contamination by late S-phase cells during cell sorting. However, this experiment is much simpler compared to MiDAS-seq and yet gives comparable results. We have described this on pages 6,52–53, lines 162,1494–1514.

Based on Fig. 2C, G2/M-seq signals are much stronger for Class-2 than Class-1, whereas MiDAS-seq signals are comparable for both (Fig. S3C). Could the authors explain this difference?

We speculate that this is due to a higher BrdU-IP signal in the untreated G2/M-seq for CFS-L compared to CFS-M, likely because of contaminating late S-phase cells (see response to your comment #14 above). Because overestimated untreated G2/M-seq signal values were used as the denominator for CFS-L in current Fig. 2D (former Fig. 2C), the CFS-L G2/M-seq log2(Aph/Untr) values were probably an underestimate. We described these points on pages 7,53, lines 209–210,1516–1522.

- 15. Page 6. "Moreover, they were frequently observed in multiple single cells, strongly suggesting that they are recurrent events (Fig. 2E)".
*Please provide a statical test to substantiate this claim.*

Thank you for pointing this. Now this is Fig. 1H. We have now performed the statistical test and added the corresponding p-values to Supplementary Table 4.

- 16. Page 7, "more so than class-3 RT-delayed breaks".
*Typo? Should be "more than…".*

The entire sentence was modified in the revised manuscript. Please check page 9, lines 265–266.

- 17. Page 7. "To test this hypothesis, we first identified the local minimum positions of RT (i.e., termination zones, TZs) in untreated conditions (Supplementary Fig. 3G), and TZs that replicated in late-S phase (RT < –0.25) were selected. Then, we performed a K-means clustering of RT surrounding these late-S TZs into two groups, which distinguished TZs with narrow RT valleys (cluster-1) from those with broad RT valleys (cluster-2) (Supplementary Fig. 3H)".
*Please provide additional details in the Methods section regarding how local minimum positions of RT were identified.*

We have now added information on the identification of the local minimum region (i.e., Termination Zones, TZs) on page 30, lines 910–916.

*Based on the data shown in Supplementary Fig. 3H, only a small number of TZs were identified (336 in*

*Cluster-1 and 395 in Cluster-2). Clarify whether this is expected. If this number is unusually low or high, explain the underlying reasons.*

Our analysis identified 1,771 TZs across the genome in untreated U2OS cells, accounting for approximately 0.06% of the genome. In our bioinformatics approach, TZs were defined as local minima along the RT profile. Specifically, a local minimum was identified as a TZ if it was flanked on both sides by at least three consecutive bins forming a descending ladder pattern toward the minimum. In addition, we further limited the analysis to those replicated in late S-phase. These stringent criteria may have limited the number of detected TZs. We have described this on page 54, lines 1532–1538.

- 18. Page 7. "In support of our hypothesis, cluster 1 but not 2 clearly showed enrichment for aphidicolin-induced G2/M replication, suggesting that narrow late-S RT valleys tend to remain under-replicated upon aphidicolin treatment (Supplementary Fig. 3H)".
*Based on Figure 2G, three classes of late regions were identified. Both Class-1 and Class-3 (RT-delayed) exhibit narrow late-S RT valleys, while Class-3 (RT-advanced) shows broad RT valleys.*
*Please clarify whether Cluster-2 in Supplementary Figure 3H corresponds to Class-3 RT-advanced regions, and whether Cluster-1 includes both Class-1 and Class-3 RT-delayed regions. If this is the case, refine the clustering analysis (e.g., use three clusters) to better align with the three categories in Figure 2G.*

We thank the reviewer for this comment. To address this point, we increased the number of k-means clusters from 2 to 5. This allowed us to identify cluster 1 with steep RT valleys, cluster 2 with moderately steep RT valleys, and cluster 5 with shallow and broad late-RT regions. These clusters closely resemble our CFS-L, non-CFS-L RT-delayed, and non-CFS-L RT-advanced break regions, respectively (Fig. 3E,F). Interestingly, we found that only cluster 1 with steep RT valleys remained under-replicated in G2/M upon aphidicolin treatment. Steepness of RT valleys reflects smaller distances covered by unidirectional forks within a given duration (i.e., slow fork movement). This suggest that slow fork movement could be yet another feature of CFSs and that the RT landscapes in untreated cells are predictive of APH-induced replication behavior and break propensity. We have described this on pages 9–10, lines 268–297.

- 19. Page 12, "We found that RPE1 and BJ cells also increased their karyotype heterogeneity upon aphidicolin treatment (Supplementary Fig. 5A,B,C)".
*Please provide a quantification to support this statement.*

We have already shown this in the former Supplementary Fig. 5C, now Supplementary Fig. 9C.

- 20. Page 12, "We identified aphidicolin-induced break regions from 329 single aphidicolin-treated RPE1 cells, with 284 having unique genomic positions. In BJ cells, 244 aphidicolin-induced break regions were identified from 173 single aphidicolin-treated cells, with 165 having unique genomic positions (Fig. 6A, Supplementary Table 1)".
*Again, before discussing aphidicolin-induced break regions, describe the total number of breaks or regions identified in untreated versus aphidicolin-treated conditions for both RPE1 and BJ cells. This contextual information is critical for understanding the scope and specificity of aphidicolin-induced breaks.*

Please refer to our response to your minor comment 6, as this is the same question for different cell lines.

- 21. Page 16, "We utilized one end of the paired-end sequenced reads".
*It is unclear why not using the paired-end reads for analyses.*

We noticed that most of the read pairs (150 bp) were overlapping, as our average DNA library size for sequencing is approximately 250 Kb. Furthermore, the paired-end sequencing cost was less expensive.

Therefore, to avoid redundant mapping and to save cost, we did paired-end sequencing but mapped only one end of the paired-end reads. We have added this detail in Methods on page 24, lines 719–720.

- 22. Page 20, "10-HMM RT was then computed from pseudo-bulk RT using RHmm v2.2.1".
*Please provide more details about this computation. Specify the parameters used, the type of HMM model applied, and the matrix used for training or testing the HMM model.*

We have now added a detailed description of the computations associated with 10-HMM RT. Please check page 26, lines 778–785.

- 23. Page 21, "HiC-Pro v3.1.0 was used to generate contact matrix from fastq files (Ref39) from which A/B compartment profile was calculated using Cooltools v0.5.4 (Ref 40)".
*Please include details about the parameters used for both HiC-Pro and Cooltools.*

We have now added the parameters used in HiC-Pro and Cooltools. Please check page 29, lines 879–884.

- 24. Page 21, "Generation of randomized control regions".
*Please clarify how the random simulations were performed. Specifically, describe how replication timing distribution was incorporated into the simulations, as this detail directly affects the robustness and interpretation of the results.*

We have now added a more detailed description on the generation of randomized controls. Please check page 30, lines 900–908.

- 25. Page 22, "In all the boxplots, the whiskers (lines extending from the box) represent the minimum and maximum values in the dataset, excluding any outliers".
*Explain how outliers were defined in the dataset. Include a clear definition or threshold to ensure transparency.*

We have now added the definition of outliers in boxplots. Please check page 32, line 961.

- 26. Page 22, "The computer codes are available from authors upon reasonable request".
*In line with open science practices, please submit the codes to a publicly accessible repository.*

We have now added a GitHub link containing scripts used in this manuscript. Please check page 33, lines 979–981.

**Reviewer #3 (Remarks to the Author):**
In this manuscript, the authors utilize a single-cell based DNA replication sequencing to map chromosomal breakage under replication stress. They categorize these identified breaks into different classes based on replication timing (RT) and location of CFSs, which further allows for a precise and comprehensive analysis of their genomic features, relative frequency, RT landscape and potential mechanisms. Overall, the findings in this paper are of interest and should offer valuable insights to further research in the field, but the mechanism is not clear enough. Nevertheless, several points and criticisms warrant attention and further clarification.

**Major concerns:**
1.Historically, fragile sites have been defined as site-specific gaps or breaks observed in mitotic chromosomes. However, in this study, the authors define chromosome breaks based on copy number variation in G1-phase cells under replication stress. Since they have not confirmed whether these breaks

actually occur during mitosis, it seems premature to equate these breaks with fragile sites at this stage. I suggest further investigation to confirm the mitotic occurrence of these breakages.

We thank the reviewer for this important comment. In our initially submitted manuscript, we showed that 39% of our aphidicolin (APH)-induced breaks in U2OS cells are enriched for APH-induced MiDAS-seq signals. MiDAS-seq is a technique to identify sites of G2/M DNA synthesis upon APH treatment, which frequently overlap with CFSs. Thus, we were confident that our APH-induced breaks correspond to CFSs. However, we understand that this may be viewed as indirect evidence. To address this concern, we have now confirmed that 45% (122/274) of our APH-induced breaks overlap with APH-induced mitotic breaks/gaps identified cytogenetically in Wilson et al., *Genome Res,* 2015 (Supplementary Fig. 1F). Thus, direct comparison of our breaks with an existing cytological CFS data strongly supports the idea that our copy-number junctions correspond to chromosome breakages. We have described this on page 5, lines 125–134.

2.The origin density and sequence characteristics of the different classes of break regions should be analyzed and discussed in more detail. The lack of replication origins and a high AT content are known to contribute significantly to CFS fragility. Addressing these aspects could strengthen the conclusions regarding the underlying mechanisms of breakage formation.

We thank the reviewer for this comment. We have reanalyzed publicly available DNA replication origin data for untreated U2OS (Wang et al., *NAR*, 2017) and found that they were enriched for our non-CFS-E RT-advanced break regions (Fig. 5B). Please check the main text on page 13, lines 391–396. As for the AT content, non-CFS-E RT-advanced and CFS-L breaks showed decreased and increased AT content, respectively ($p < 0.001$ compared to RT-matched control regions). However, since the median size of our breaks is 270 Kb, the interpretation of base composition analysis data seemed tricky, and therefore, we did not pursue this further.

3.As class-5 break regions show early-replicating and no enrichment for aphidicolin-induced G2/M-seq signals, do they correspond to early replicating fragile sites? A correlative analysis may help to answer this question.

We thank the reviewer for this comment. We found that both RT delayed and advanced non-CFS-E break regions are located close to ERFS sites (Supplementary Fig. 7D). This is described on page 14, lines 407–412.

4.The authors suggest that class-5 breaks with advanced replication timing display characteristics of transcription-replication conflicts (TRC) based on public datasets. However, additional experimental validation is needed to determine whether TRC is the primary driver of these breaks. For example, regulating TRC levels through transcription inhibition and assessing breakage formation would provide more direct evidence.

We thank the reviewer for this suggestion. We have now added experiments to show that DNA damage at non-CFS-E RT-advanced break regions decreases upon transcription inhibition by DRB (Fig. 5C–G). This supported our view that non-CFS-E RT-advanced breaks are likely due to TRCs. This is all described in the main text (on pages 14–15, lines 419–439) and in Methods (on pages 27–29, lines 799–868).

5.It's been reported that CFS stability is compromised at TAD boundaries (PMID: 32680994), what's the relative location of TAD boundary with different classes of breakages? Did TAD organization affect breakage formation?

Contrary to Sarni et al., 2020 (PMID: 32680994), our break classes were not significantly closer to TAD

boundaries compared to randomized controls with similar RT, except for non-CFS-E (Supplementary Fig. 5C). However, the median distance of non-CFS-E breaks to TAD boundary is 30 Kb compared to 10 Kb of RT matched controls, which seem like only a slight difference (Supplementary Fig. 5C).

Interestingly, we found that the A/B compartment landscape could help in predicting replication behavior and possibly break-prone regions. That is, non-CFS-M RT-delayed breaks were frequently present at A/B compartment boundaries (Fig. 4D), and non-CFS-E RT-advanced breaks corresponded to broader A-compartment compared to RT-delayed ones (Supplementary Fig. 7H). These observations suggest that the A/B compartment landscape could be more important than TAD boundaries. We have described this on pages 8,54, lines 214–220,1540–1555.

**Minor comments:**

1.Supplementary Figure 3: the authors analyze replication activity through BrdU-seq of G2/M-phase cells based on DAPI staining. However, this method cannot completely exclude late S-phase cells, which also exhibit G2/M DNA content. To be more precise, it would be better to describe this as "BrdU-IP of late S/G2/M-phase cells" to more accurately reflect the experimental approach.

Yes, it is correct. We have renamed it to late-S/G2/M-seq in our revised manuscript. Please check page 6, line 162.

2.The sorting strategy used in CBMS1 mouse embryonic stem cells for scRepli-seq and G2/M-seq should also been presented.

We have now added this information in supplementary Fig. 10A,B. Please check page 19, lines 559,564 for changes in the text.

**Reviewer #4 (Remarks to the Author):**

I co-reviewed this manuscript with one of the reviewers who provided the listed reports. This is part of the Nature Communications initiative to facilitate training in peer review and to provide appropriate recognition for Early Career Researchers who co-review manuscripts.

Thank you for your consideration. Please feel free to contact me with any concerns.

Sincerely yours,

Ichiro Hiratani, Ph.D.
Team Director (Principal Investigator)
Laboratory for Developmental Epigenetics
RIKEN Center for Biosystems Dynamics Research (RIKEN BDR)
2-2-3 Minatojima-minamimachi, Chuo-ku, Kobe, 650-0047 JAPAN
phone: +81-78-306-7179
Email: ichiro.hiratani@riken.jp
https://www.bdr.riken.jp/en/research/labs/hiratani-i/index.html

Below is the point-by-point response to all comments raised by the reviewers in the second round (reviewer comments in black, our response in blue, and changes in the manuscript in red). We look forward to your response.

**Reviewer #1 (Remarks to the Author):**

The authors have mostly addressed the comments raised by the Reviewers.

From my side there were two main points: whether the classification of aphidicolin-induced breaks leads to more types than supported by the real biology and the limited information on mechanistic insights. The authors present 5 types, 3 of which have been described below (late S-replicating CFS; mid S-replicating CFS; early S-replicating non-CFS). The last type is shown in this revised manuscript to correspond to the early-replicating fragile sites (ERFS) described by Nussenzweig et al several years ago. The remaining 2 types (mid S-replicating non-CFS and late S-replicating non-CFS) appear to be novel.

Regarding mechanistic insights, the authors describe new experiments treating cells with DRB to inhibit transcription elongation. These results argue that early S-replicating non-CFS sites (Fig. 5C) are due to transcription-replication conflicts. The mid S-replicating non-CFS sites (Fig. 4B) behave very similar to the early S-replicating non-CFS sites (Fig. 5C) and may be mechanistically similar.

While transcription-replication conflicts (TRCs) could be involved in both mid-S and early-S replicating non-CFS (non-CFS-E and non-CFS-M) sites, we presented several pieces of evidence suggesting that they could be mechanistically distinct. Unlike non-CFS-E RT advanced breaks, which are enriched for replication initiation activity, non-CFS-M RT delayed breaks are frequently present at replication timing transition regions (TTRs) that are devoid of initiation events (Fig. 4A,D, and 5B). In addition, non-CFS-M RT delayed breaks are not enriched for DNA secondary structures or aphidicolin (APH)-induced FANCD2 binding (Fig. 5B), and showed a slightly higher degree of under-replication in G2/M phases (Fig. 4A; and Supplementary Fig. 6D). Based on this series of observations, we believe there could be mechanistic differences between non-CFS-E and non-CFS-M classes. This is described on pages 12 and 20–21, lines 352–355 and 608–616.

The surprising observation relates to the late S-replicating non-CFS sites, where aphidicolin does not induce a DNA damage response, as would be expected; instead DRB induces DNA damage (Fig. 3D). I am sceptical of these data. The authors should discuss this finding. It could be that early S-replicating, mid S-replicating and late S-replicating non-CFS breaks are all due to transcription-replication conflicts. The data suggest that this is true for early S and mid S non-CFS breaks. For late S non-CFS breaks, the number of breaks was probably low (as suggested by Fig. 1G) and the collected data may not be

accurate, as they do not make sense.

Overall, the manuscript has been improved and can be published. Ideally, the issue of Fig. 3D should be addressed. If the authors are not able to generate reliable data, they should not show these data.

We thank the reviewer for raising this important point.

We performed Immuno-FISH experiments at two loci from different chromosomes, with independent biological replicates showing reproducible results. Based on this, we are confident of the observed results of late S-replicating non-CFS (non-CFS-L) RT-delayed breaks.

That said, we agree that the results of non-CFS-L sites are unexpected and warrant further discussion. Notably, DRB treatment increased γH2AX signal across multiple break classes, indicating that transcription inhibition alone can induce DNA damage. Under APH-induced replication stress, transcription inhibition appears to decrease DNA damage at non-CFS-E and non-CFS-M sites, consistent with a role for APH-induced TRCs in the formation of breaks at these sites. However, non-CFS-L sites do not follow this pattern. One possible explanation is that, at non-CFS-L sites, replication stress (by APH) and transcription-associated stress (by DRB) may be less directly coupled, with transcriptional stress as the dominant source of damage. We understand that this interpretation is speculative and have included this point in the discussion section on page 20, lines 605–608.

The journal could be Nature Communications or Genome Biology as decided by the editors.

Given its methodological innovation and broad implications for genome stability, DNA replication biology and 3D genome organization, we believe that our study is well-suited for *Nature Communications*, where it can reach a wider and more diverse readership than more specialized journals such as *Genome Biology*.

**Reviewer #2 (Remarks to the Author):**

Compared with the previous version, the revised manuscript by Elumalai and Hiratani has been significantly improved. The authors have added new analyses and experimental data, including a clearer comparison between late-S/G2/M-seq and MiDAS-seq, improved comparisons between 10-cell Repli-seq and conventional two-state BrdU-IP RT profiling, additional analyses of break types, and more systematic characterization of non-CFS classes relative to canonical CFSs. The manuscript also includes improved comparisons with previous studies and new experiments examining the effect of transcription inhibition. Overall, these additions strengthen the manuscript. However, several important issues still need to be addressed and clarified before the manuscript can be further considered.

We thank the reviewer for their positive evaluation of our revised manuscript and for acknowledging the additional analyses and experiments included in the revised manuscript.

1. Comparison between MiDAS-defined CFSs and Aph-induced breaks

Based on the revised analysis, only 16% of MiDAS-seq CFSs overlap with Aph-induced breaks identified in this study (Fig. S1F). This relatively low overlap requires further explanation.

We believe that the following factors likely contribute to this observation.

- Our chromosome break analysis is based on a limited number of single cells treated with aphidicolin (APH; n = 43), which may reduce the likelihood of detecting recurrent breaks and therefore underestimate the true overlap with MiDAS-defined CFSs.
- In our current analysis, we focused on APH-specific breaks by excluding regions proximal to

untreated breaks, potentially removing a subset of bona fide CFSs shared between untreated and APH-treated conditions. Consistent with our hypothesis, we found that 24% (76/324) of MiDAS-seq's CFSs overlapped with inherent breaks (defined as those shared between untreated and APH-treated). Together, we found that 40% (129/324) of MiDAS-seq CFSs overlapped with our breaks in APH-treated cells.

We have discussed these points in Supplementary Note 3 of the revised manuscript, on page 53, lines 1530–1538.

On the other hand, the overlap between Aph-induced breaks identified in U2OS cells in this study and cytogenetically identified CFSs from fibroblasts appears to be higher (Fig. S1F). This is somewhat unexpected, as it is well established that CFSs are cell-type specific. In principle, datasets generated from the same cell type should show higher concordance.

A possible explanation could be differences in resolution between cytogenetic CFS mapping and sequencing-based datasets. However, this discrepancy is not discussed in the manuscript. The authors should explicitly address this issue and discuss potential explanations.

As the reviewer rightly pointed out, the resolution of sequencing-based CFSs (average size = 0.6 Mb) was higher than that of cytogenetically-defined-based CFSs (average size = 11.5 Mb). This could have resulted in a greater overlap between our break regions and cytogenetically-defined-based CFSs than with sequencing-based CFSs.

In addition, although CFSs have often been described as cell-type-specific, many of these conclusions are based on cytogenetic analyses of a limited number of well-characterized CFS loci. More recent sequencing-based studies suggest that a substantial fraction of CFSs are conserved across cell types (e.g., Ji et al.; Macheret et al., *Cell Res.* 2020). These findings indicate that both conserved and cell-type-specific fragile regions exist.

Therefore, the greater overlap of CFSs across cell types may reflect a combination of resolution differences between cytogenetics and sequencing-based techniques, as well as the presence of conserved CFSs. We have discussed these points in Supplementary Note 3 of the revised manuscript on page 53, lines 1540–1550.

2. Potential bias introduced by excluding "inherent breakpoints"

The authors define aphidicolin-induced break regions as those that do not overlap with, or lie within ±1 Mb of, untreated break regions, in order to remove "inherent breakpoints".

However, it is unclear how this filtering step may affect downstream analyses. Importantly, inherent breakpoints present in U2OS cells are unlikely to be randomly distributed across the genome and may themselves correlate with replication timing or other genomic features. Therefore, excluding all regions within ±1 Mb of untreated breakpoints could potentially introduce biases into the analyses of aphidicolin-induced breaks.

As raised previously, the authors should perform a systematic characterization of inherent breakpoints, including their genomic distribution and their relationship to MiDAS-defined CFSs and RT profiles, before excluding them from downstream analyses. This would be necessary to demonstrate that the filtering strategy does not introduce unintended biases.

We thank the reviewer for this suggestion. To address this point, we identified inherent break regions of U2OS, defined as APH-induced breaks that overlapped with or were located within +/– 1 Mb of untreated breaks. This analysis resulted in the identification of 3,316 inherent breaks in 43 APH-treated cells, of which 260 break regions were non-redundant. Next, we categorized the inherent break regions

on the RT–MiDAS axes, as in the classification of APH-induced breaks, and found no specific enrichment for any particular break class (Supplementary Fig. 3F,G). These results suggest that, similar to APH-induced breaks, multiple break categories exist among inherent breaks, and that our definition of APH-induced breaks does not introduce any bias. This is described on pages 4, 7, and 26, lines 99–101, 181–183, and 773–775.

3. Clarification of whether and how RT was controlled in the randomized controls

In several analyses (e.g., Fig. S3F and Fig. 7F), randomized genomic regions are used as controls. However, it is not clearly stated whether the replication timing (RT) distribution of breakpoints was taken into account during the randomization procedure.

Because many genomic features analyzed in this study are strongly correlated with RT, it is critical that the randomized controls match the RT distribution of the observed breakpoints. Otherwise, apparent enrichments or depletions may simply reflect RT bias.

The authors should explicitly clarify whether RT was incorporated into the randomization strategy for these analyses. If RT was not taken into account, the relevant analyses should be repeated using randomized controls that preserve the RT distribution of the observed breakpoints.

A similar control analysis would also be required for the analyses involving the mESC datasets.

We have now added a similar break vs. random analysis for each RT–G2/M category in mESCs (Supplementary Fig. 10D). Please see page 19, lines 573–574.

For details on how RT distribution was controlled in the randomized controls, please refer to our response to your comment #4.

4. The current randomization strategy does not appear to adequately control RT

Related to the point above, the description of the randomization procedure in the Methods suggests that RT is not directly controlled during randomization.

According to the Methods, randomized regions were generated using bedtools shuffle, followed by classification of these regions into early/mid/late S-phase using pseudobulk RT profiles. However, this procedure represents post hoc categorization rather than RT-matched randomization.

In other words, the randomized regions appear to be generated genome-wide and only afterward assigned to RT categories, which does not ensure that the RT distribution of the randomized controls matches that of the observed breakpoints.

Because replication timing is a major determinant of many genomic features analyzed in this study, failure to properly control RT in the randomization procedure could substantially affect the interpretation of the results.

The authors should clarify this point and, if necessary, revise the randomization strategy to ensure that the RT distribution is properly controlled in analyses relying on these randomized controls.

We thank the reviewer for raising this important point. We agree that post hoc categorization alone does not, in principle, guarantee that the RT distribution of randomized regions matches that of the observed breaks. To address this, we examined the RT distributions of the observed breaks and the corresponding randomized control regions using continuous RT values in early-, mid-, and late-S phases. We found that the RT distributions of the randomized controls closely match those of the observed breaks, and do not show a statistically significant difference (Supplementary Fig. 5A), indicating that the current

randomization procedure does not introduce a detectable bias in RT. We have included this additional validation in the revised manuscript (page 31, lines 926–928).

While we acknowledge that controlling RT during randomization is an alternative approach, our results indicate that our current strategy provides adequate control for RT in the context of our analyses.

5. Interpretation of transcription inhibition experiments

The newly added transcription inhibition experiments are interesting, while several aspects require further clarification.

In the Immuno-FISH analysis, the authors quantify the "logarithmic γH2AX signal per FISH spot per nucleus". However, the method used to calculate this value is not clearly described. It is unclear whether the logarithmic transformation was applied to raw intensity values or to normalized intensities (e.g., relative to nuclear signal). This information should be clarified.

For the Immuno-FISH analysis, γH2AX signal intensities at each FISH spot were calculated and averaged per nucleus. Averaged γH2AX signals were normalized to the mean nuclear γH2AX intensity of untreated cells within the same experiment. The normalized values were then $\log_2$-transformed. The reported values, therefore, represent $\log_2$-transformed γH2AX intensities at FISH spots relative to the untreated baseline. We have clarified this in the Methods section on page 29, lines 881–885.

In addition, the rationale for applying a logarithmic transformation is not explained. While log transformation may be useful for handling skewed intensity distributions, it would be helpful to know whether the conclusions remain consistent when using the raw or normalized intensity values. Given that most values shown in the figures (e.g., Fig. 3C/D, Fig. 4B/C, Fig. 5C) fall within a relatively narrow range (approximately −1 to 1 on the $\log_2$ scale for CFS-L and −0.5 to 0.5 for the other classes), it would also be informative to assess whether the observed differences remain evident when plotted using the original intensity values.

The logarithmic transformation was applied to facilitate interpretation in terms of relative (fold) changes with respect to the untreated baseline. We have clarified this on page 29, lines 885–886.

In addition, to address the reviewer's concern, we repeated the analysis using both normalized and original intensity values without log transformation. We found that the overall trends and relative differences between conditions remain consistent, indicating that the conclusions are not dependent on the use of log transformation or normalization (**see the Figure on the next page**).

**Figure Legend (Figure on the next page):** Boxplots with jitters showing mean γH2AX signals (top), normalized γH2AX signals (middle), and logarithmic normalized γH2AX signals (bottom) at different break classes as shown at the top. Two representative break regions (BAC #1 and #2) are shown for each break class. *P*-values were obtained from a two-sided unpaired t-test. Treatment conditions: untreated, grey; DRB, black; aphidicolin, red; aphidicolin+DRB, crimson. *N*, number of nuclei.

**Representative BAC #1**

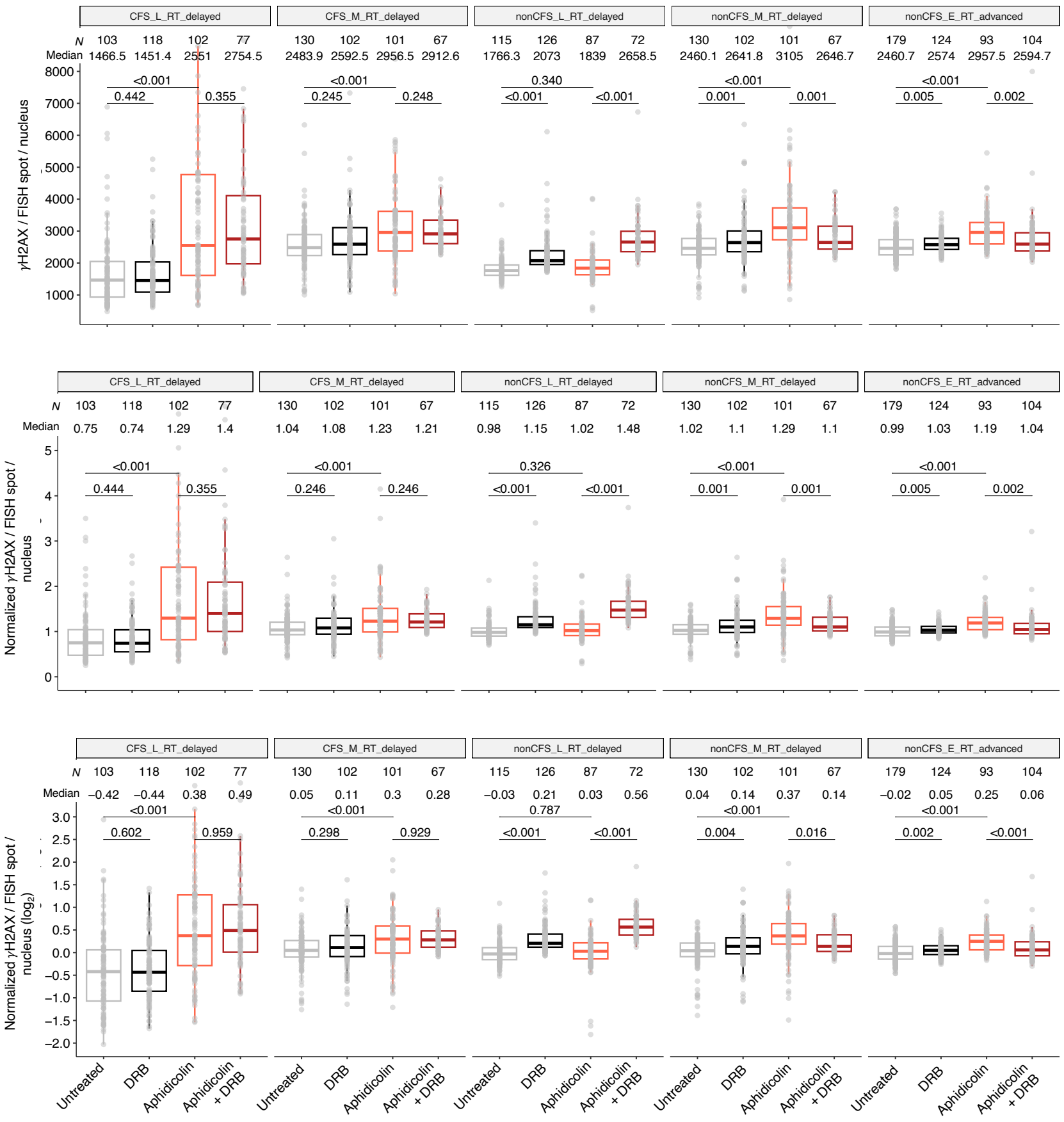


**Representative BAC #2**

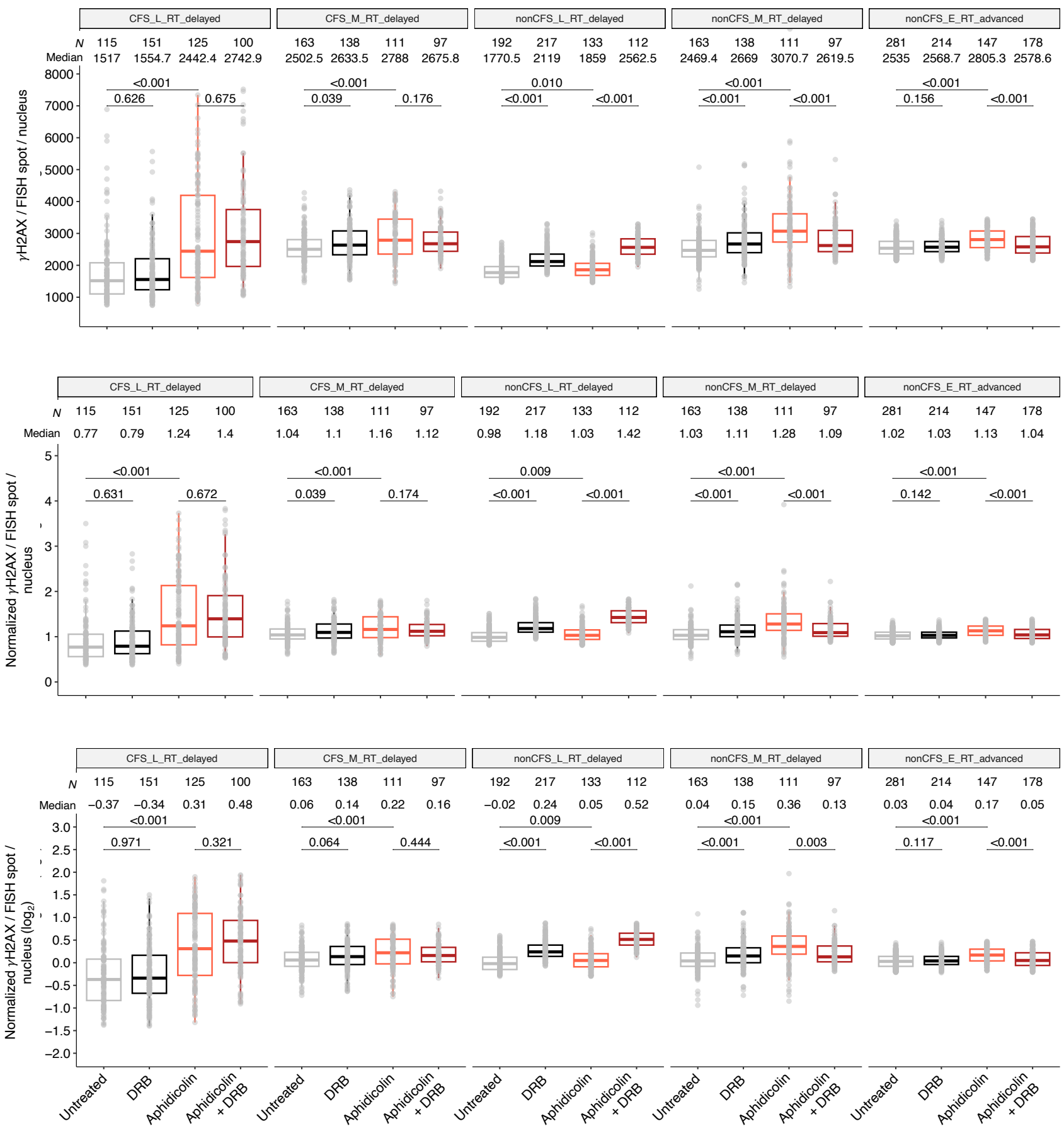

Additionally, in the Immuno-FISH analysis, DNA-FISH signals were identified using the "Find Maxima" function in Fiji. Because this approach detects local intensity maxima and can be sensitive to noise and background fluctuations, it would be helpful if the authors could provide additional details on the parameters used (e.g., noise tolerance) and how the robustness of spot detection was ensured.

DNA-FISH signals were identified using the "Find Maxima" function in Fiji with a noise tolerance threshold of 3,000, which was determined empirically to reliably detect true FISH foci while minimizing background noise. In addition, given the hypertriploid nature of U2OS cells, we applied a filtering step to include only nuclei with 2–8 FISH foci for downstream analysis. We have described this in the Methods section on page 29, lines 877–881.

6. Further clarification of transcription inhibition experiments

In the Immuno-FISH analysis, asynchronous U2OS cells were treated with aphidicolin for 24 h, with DRB added during the last 4 h to inhibit transcription. The results show that transcription inhibition has little effect on γH2AX levels at CFS-associated classes but strongly alters γH2AX levels at non-CFS-L regions.

Several aspects of this experiment require further clarification and discussion.

First, the cell-cycle distribution of the analyzed population after this treatment regime is not described. Given the prolonged aphidicolin treatment and the asynchronous cell population, the cell-cycle composition could strongly influence the observed γH2AX signals.

Immuno-FISH analysis was performed on asynchronous U2OS cells. To assess cell-cycle distribution, we performed FACS analysis and found little to no difference with or without DRB treatment under both untreated and APH-treated conditions (Supplementary Fig. 5H). We have described this on page 9, lines 246–248.

Second, the authors' analyses indicate that non-CFS-L loci exhibit relatively low transcription levels (e.g., GRO-seq in Fig. 2F). It is therefore not immediately obvious why transcription inhibition would strongly affect γH2AX levels at these regions. This point should be further discussed.

While non-CFS-L loci show lower transcriptional activity compared to CFS-L regions, their transcription levels are comparable to those of RT-matched randomized controls. Therefore, we cannot conclude that non-CFS-L loci are transcriptionally less active than other genomic regions.

Third, DRB treatment alone appears to increase γH2AX levels at non-CFS-L loci, raising the possibility that the observed effects may arise from indirect consequences of transcription inhibition, such as alterations in cell-cycle progression or replication programs. These possibilities should be carefully examined and discussed.

As mentioned earlier, we did not observe differences in cell-cycle progression. However, we agree that this point requires further clarification. Our response is largely consistent with that provided to Reviewer #1. DRB treatment increased γH2AX signals across multiple break classes, indicating that transcription inhibition (i.e., transcriptional stress) alone can induce DNA damage. Under APH-induced replication stress, transcription inhibition appears to reduce DNA damage at non-CFS-E and non-CFS-M sites, consistent with a role for APH-induced TRCs in break formation at these sites. In contrast, non-CFS-L sites do not follow this pattern. One possible explanation is that, at non-CFS-L sites, replication stress (induced by APH) and transcription-associated stress (induced by DRB) are less directly coupled, with transcriptional stress potentially serving as the dominant source of damage. We acknowledge that this interpretation is speculative and have included this point in the Discussion section on page 20, lines 605–608.

7. Interpretation of predictive performance

In Fig. 6C, the reported AUC values range from approximately 0.6 to 0.75. While these values suggest some degree of predictive signal, they generally indicate only modest discriminatory power. The current wording in the manuscript may therefore overstate the predictive performance of the model, and the corresponding claims should be moderated accordingly.

We have revised the text throughout the manuscript to more appropriately reflect the moderate predictive performance. Please see pages 16, and 21, lines 470–471, and 634.

In addition, each model appears to be represented by a single AUC estimate without an associated confidence interval or measure of variability. Because AUC estimates can depend on sampling variation, it would be helpful to report uncertainty estimates (e.g., bootstrap-based 95% confidence intervals or cross-validation results). This would allow readers to better assess the robustness of the reported predictive performance and the significance of differences between models.

We thank the reviewer for this suggestion. We have now incorporated bootstrap-based estimation of AUC variability and report 95% confidence intervals for all models (Fig. 6C). Importantly, these analyses indicate that the variability is small and does not affect the overall results or conclusions. We have described this on page 32, lines 963–966.

8. Use of the term “rare fragile site”

The term “rare fragile site” (used in the Abstract) already has a well-defined meaning in the fragile site field (e.g., FRAXA associated with fragile X syndrome). Using this term to describe the break classes identified in this study may lead to confusion. The authors should avoid reusing terminology that already has an established definition in the field.

We were careful not to use the term “rare fragile sites” for the same reasons noted by the reviewer; accordingly, we used the expression ‘rare’ fragile sites (with quotation marks) to avoid confusion with the established terminology. However, as this wording may still be confusing, we have now completely avoided this expression and replaced it with “low-frequency fragile sites (non-CFSs).” Please see page 1, line 14.

9. Clarity of terminology related to scRepli-seq and scWGS

I understand that the authors may wish to emphasize the previously developed scRepli-seq framework. However, the current wording may create confusion regarding the method used in this study.

For example, the Introduction states: “To perform an unbiased, comprehensive mapping of fragile sites, we employed scRepli-seq for karyotyping…” In practice, break detection in this study is based on single-cell whole-genome sequencing (scWGS) of G1 cells, which is conceptually distinct from replication timing profiling. For clarity, this sentence could be revised to explicitly state that scWGS (implemented within the previously developed scRepli-seq framework) was used for karyotyping and break detection.

Similarly, the Results section title “Single-cell karyotyping by scRepli-seq” would be more accurately described as “Single-cell karyotyping by scWGS of G1 cells”.

The authors should revise related descriptions throughout the manuscript, including figure legends where appropriate.

Done. Please see pages 2 and 3, lines 49–50, 54–55, 64, and 80, and Fig. 1A.

I hope that these comments and suggestions will help further improve the rigor and reproducibility of the manuscript.

**Reviewer #3 (Remarks to the Author):**

Upon my initial evaluation, I recognized the technical rigor of this study, notably its application of scRepli-seq to comprehensively map and characterize chromosomal breakage under conditions of replication stress. While the findings hold relevance for the field, my central concern—namely, the absence of a clear mechanistic framework—has not been satisfactorily resolved in the current revision.

The revised version remains predominantly descriptive. It effectively delineates correlations between genomic features and classes of breaks, yet it does not advance beyond these associations to establish causality or to formulate and validate a novel regulatory model. Although the analyses are executed thoroughly, they principally serve to confirm or extend established paradigms connecting replication timing, transcription, and sequence features with genomic fragility.

As discussed in the summary and highlights section on the first two pages of our previous response letter, the novelty of our study lies in the identification of new classes of fragile sites that are distinct from CFSs and ERFSs. In addition, we have demonstrated that RT landscapes in untreated cells can predict APH-induced RT changes and break propensity. Furthermore, our transcription inhibition experiments supported the conclusion that these newly identified break classes may be mechanistically distinct from previously reported CFS and ERFS break classes. While we fully acknowledge that additional experiments will be required to elucidate the precise mechanisms underlying break formation across different classes, we consider this an important direction for future investigation, both by our group and by the field more broadly.

The authors have included a comparative analysis of their APH-induced breakpoints with cytogenetic fragile site data from Wilson et al. (2015). While the reported 45% overlap is suggestive, this validation remains indirect and relies on cell-line mismatches. A more definitive approach would entail performing cytogenetic validation (e.g., metaphase spreads) specifically in U2OS cells under identical experimental conditions, or aligning the data with existing U2OS-specific cytogenetic maps. Direct evidence obtained in a matched cellular context is essential to robustly bridge the high-throughput sequencing findings with the classical cytogenetic definition of mitotic fragile sites. I recommend the authors address this point either through additional experiments or, if further cytogenetic validation is not feasible, through a more thorough discussion of this limitation.

CFSs are often described as cell-type-specific, and many reports are based on cytogenetic analyses focusing on a limited number of well-characterized CFS loci. However, more recent sequencing-based studies have suggested that a substantial fraction of CFSs are conserved across cell types (e.g., Ji et al.; Macheret et al., *Cell Research*, 2020). Therefore, to address potential concerns regarding cell-line differences, we compared cytogenetically-defined and sequencing-based chromosome break datasets across multiple cell lines. We have described this on page 53, lines 1540–1550.

Despite the authors' efforts during revision, the study still falls short of the required threshold for novelty and mechanistic depth. Therefore, I would not make a strong case for Nature Communications, though it may be more appropriately aligned with the scope of Genome Biology.

As noted above, we leveraged a cutting-edge technology that we developed to investigate chromosome breakage under mild replication stress. As such, we are uniquely well positioned for this analysis, with

the technical expertise to perform scRepli-seq with a high degree of control and precision. Based on our data, we identify previously unrecognized classes of fragile sites that are distinct from CFSs and ERFSs. Furthermore, we demonstrate that RT landscapes in untreated cells can predict both APH-induced RT changes and break propensity. While we fully acknowledge that additional experiments will be required to elucidate the precise mechanisms underlying break formation across different classes, we consider our work to provide an important conceptual and technical foundation for understanding the nature of low-frequency fragile sites.

Given its methodological innovation and broad implications for genome stability, replication biology, and 3D genome organization, we believe that our study is well-suited for *Nature Communications*, where it can reach a wider and more diverse readership than more specialized journals such as *Genome Biology*.

**Reviewer #4 (Remarks to the Author):**

I co-reviewed this manuscript with one of the reviewers who provided the listed reports. This is part of the Nature Communications initiative to facilitate training in peer review and to provide appropriate recognition for Early Career Researchers who co-review manuscripts.

We thank the reviewer for their time and valuable suggestions.

Thank you for your consideration. Please feel free to contact me with any concerns.

Sincerely yours,

Ichiro Hiratani, Ph.D.
Team Director (Principal Investigator)
Laboratory for Developmental Epigenetics
RIKEN Center for Biosystems Dynamics Research (RIKEN BDR)
2-2-3 Minatojima-minamimachi, Chuo-ku, Kobe, 650-0047 JAPAN
phone: +81-78-306-7179
Email: ichiro.hiratani@riken.jp
https://www.bdr.riken.jp/en/research/labs/hiratani-i/index.html

Thank you very much for handling our manuscript entitled “Single-cell mapping of chromosome breaks identifies multiple fragile site classes with distinct DNA replication timing landscapes”. Once again, we appreciate the constructive feedback provided by the reviewers. We have carefully addressed all reviewer comments with additional analyses where required.

Below, we provide a point-by-point response to all comments raised by the reviewers during the third round of review (reviewer comments in black, our response in blue, and changes in the manuscript in red). We look forward to your response.

**Reviewer #2 (Remarks to the Author):**

The authors have made substantial efforts to address my previous comments and have provided additional analyses and experimental data that improve the manuscript. In particular, the inclusion of analyses on inherent breaks and additional Immuno-FISH FACS data is appreciated. However, several mechanistic interpretations remain speculative and are not yet directly supported experimentally. The discussion should more clearly distinguish between experimentally supported conclusions and proposed interpretations. The following important issues still require clarification before several of the central conclusions can be fully supported.

We thank the reviewer for their positive evaluation of our revised manuscript and for acknowledging the additional analyses and experiments included in the revision. We also appreciate the reviewer's concern regarding the distinction between experimentally supported conclusions and proposed interpretations. In the revised manuscript, we have made every effort to more clearly separate data-supported conclusions from speculative mechanistic interpretations, as detailed below.

Major comments

1. Interpretation of aphidicolin-dependent break classes

I appreciate that the authors now included the analysis of inherent breaks from U2OS cells. Comparing the proportions of aph-induced breaks (N = 274) and inherent breaks (N = 3316) with randomized controls for different break classes, it is clear that only the CFS-L and CFS-M classes show stronger enrichment in aph-induced samples relative to inherent breaks, which is consistent with the established aphidicolin dependency of canonical CFSs.

However, the current analyses demonstrate the existence of multiple break classes among both aph-induced and inherent breaks, but they do not yet clearly establish that the non-CFS classes are preferentially induced by aphidicolin, rather than reflecting constitutive fragile regions with limited detection sensitivity in untreated samples because of the smaller sample size. Additional discussion is therefore needed to address this important point. In particular, the later Immuno-FISH experiments do not sufficiently demonstrate that these classes represent bona fide aph-dependent break events (see next comment).

To facilitate comparison, the percentage of inherent breaks should be added to the bar plot in Supplementary Fig. 3H alongside aph-induced breaks and randomized controls. Similar analyses should also be included in Fig. 7F for RPE1 and BJ cells, as well as Supplementary Fig. 10D for mESCs.

We thank the reviewer for this insightful comment. As the reviewer correctly pointed out, aphidicolin (APH)-induced non-CFS breaks are rare events (Fig. 1H). Thus, the absence of a break region in untreated cells does not necessarily lead to the conclusion that the region is uniquely induced by APH, as low-frequency break events, including constitutive fragile regions, may remain undetected because of finite sampling. Consequently, it is difficult to determine whether a given non-CFS break region is exclusively induced by APH.

Although we agree that our data do not allow us to unequivocally determine whether the break regions detected in APH-treated cells were newly generated by APH treatment or instead represent pre-existing low-frequency constitutive fragile regions, APH-dependent fragility should nevertheless be reflected by an increase in break frequency following APH treatment.

We therefore quantified break frequencies at all break regions identified in APH-treated cells, which are expected to include both break regions newly generated by APH treatment and pre-existing constitutive fragile regions with a range of baseline break frequencies. Specifically, break regions were categorized by their positions on the RT-MiDAS axes, and for each region, we calculated the percentage of single cells carrying a chromosome break within that region in untreated and APH-treated conditions. We found that all five break classes exhibited increased break frequencies following APH treatment in three human cell lines (Supplementary Fig. 4A–D, human U2OS, RPE1, and BJ cells; note that mouse ESCs showed a similar trend but did not reach statistical significance because of the limited sample size), indicating that APH-induced replication stress is associated with increased instability not only at canonical CFSs but also across all non-CFS classes.

Taken together, the observed increase in break frequency across all non-CFS classes following APH treatment indicates that the enrichment of non-CFS breaks in APH-treated cells is unlikely to be explained solely by the limited detection sensitivity of these breaks in untreated samples due to finite sampling. Rather, our results are consistent with the interpretation that replication stress enhances instability at both CFS and non-CFS regions. We have revised the manuscript to clarify this point and to emphasize that APH treatment enhances the manifestation of replication-stress-associated instability at susceptible genomic regions. **These points are now discussed on page 7, lines 188–194, and in Supplementary Note 5.**

Let us discuss your Immuno-FISH-related point in our response to your next comment.

Regarding your last point on bar plots, **we have added the percentage of inherent breaks to the relevant bar plots as requested (Supplementary Fig. 3H, Fig. 7F, and Supplementary Fig. 11D)** to facilitate direct comparison among inherent breaks, APH-induced breaks, and randomized control regions. We note that U2OS MiDAS-seq data were used for break categorization in RPE1 and BJ cells; therefore, this point should be considered when interpreting the relative ratio of break classes. We have already discussed this point in the discussion section of the manuscript. **Please see page 22, lines 667–673.**

2. Potential confounding effects of altered cell-cycle distribution in Immuno-FISH experiments

The newly added FACS profiles in Supplementary Fig. 5H are useful. I agree that DRB treatment itself does not appear to substantially alter the cell-cycle distribution in either aph-treated or untreated cells. However, aphidicolin treatment clearly changes the cell-cycle profile, with substantial accumulation of cells in S and G2/M phases. This issue should be carefully considered when interpreting the Immuno-FISH results. At minimum, the limitations introduced by altered cell-cycle composition should be more explicitly acknowledged when interpreting γH2AX enrichment as evidence of aphidicolin-dependent

break formation.

For example, for the non-CFS-M RT-delayed breaks (Fig. 4B), the authors propose that these breaks arise from transcription-replication conflicts. If so, stronger γH2AX signals would be expected preferentially in S-phase cells. Therefore, it is unclear whether the increased γH2AX signals observed in aph-treated samples simply reflect the higher proportion of S-phase cells, rather than increased break formation specifically induced by aphidicolin. Without additional controls accounting for cell-cycle composition, it remains difficult to conclude whether these represent genuinely aphidicolin-induced break events.

A similar issue applies to the non-CFS-L RT-delayed breaks (Fig. 3D). If DRB-dependent breaks are enriched in S or G2/M cells, the stronger γH2AX signals observed in the Aph+DRB condition relative to DRB alone could potentially be explained by altered cell-cycle distribution rather than a direct effect on break formation.

We thank the reviewer again for this insightful comment. We agree with the reviewer that APH alters cell-cycle distribution substantially and that this represents a potential confounding factor when interpreting γH2AX signals at representative non-CFS loci in asynchronous cell populations. There is indeed a possibility that γH2AX signal enrichment might simply reflect altered cell-cycle composition.

To directly assess whether different cell-cycle phases are associated with different γH2AX levels, we categorized nuclei into distinct cell-cycle phases based on their DAPI and EdU imaging signals and examined whether S- or G2/M-phase cells exhibited elevated γH2AX signals relative to the asynchronous population mean (Fig. 5D and Supplementary Fig. 8H–J). Interestingly, we did not observe a substantial increase in γH2AX signals in S- or G2/M-phase cells under either untreated or DRB-treated conditions (Supplementary Fig. 8I–J). Thus, our data suggest that enrichment of S- or G2/M-phase cells alone is unlikely to fully explain the increased γH2AX signals observed at the analyzed loci in APH- or APH+DRB-treated cells. These points are now discussed **on page 15, lines 449–454 and Supplementary Note 9.**

3. Presentation of raw γH2AX signals

With the additional explanations, I now better understand how the Immuno-FISH data were processed. Since the authors have already performed these analyses during revision, inclusion of the raw intensity plots in the manuscript would substantially improve transparency and interpretability.

The raw data provide more direct and informative comparisons between conditions without requiring additional normalization procedures. Importantly, they also reveal biologically relevant features that are obscured in the normalized presentation. For example, raw γH2AX signals appear lower for BAC probes located in late-replicating regions (e.g., CFS-L_RT-delayed and nonCFS-L_RT-delayed regions) compared with probes in mid- or early-replicating regions (if we compare the untreated samples). This observation could reflect either biological differences or technical limitations, and the authors should comment on and discuss this point.

We think that the differences in raw γH2AX signals are likely due to batch effects, as all break classes (i.e., BAC probes) were not processed and imaged on the same day because of the large number of BAC probes. Therefore, we normalized the values using the mean nuclear γH2AX intensity of untreated cells within the same experiment. Plots using normalized γH2AX values showed similar distributions and median values across break classes except for CFS-L RT-delayed breaks (please see the figure in our previous response letter, untreated condition). As such, we believe that using plots with normalized values in the manuscript will enable accurate comparison. To improve transparency, we have provided the source data file which includes both raw and normalized values for different break classes (Supplementary Table 5). We have added these points to the methods section on page 30, line 902.

4. Overstatement of predictive power throughout the manuscript

There are still numerous instances where the manuscript overstates the predictive performance of the analyses. In several cases, the analyses are better described as demonstrating associations or partial discriminatory power rather than prediction. This issue should be carefully revised throughout the text.

For example, in the sections entitled "Genomic/epigenomic features can predict aphidicolin-induced RT changes" and "Late-S RT landscapes can predict the replication behavior in response to aphidicolin", the presented analyses mainly demonstrate positive or negative correlations. However, no true predictive framework has been established, nor has any rigorous predictive modeling analysis been performed to demonstrate substantial predictive power for aphidicolin-induced RT changes. These analyses are largely correlative, and the current data do not yet establish predictive performance. Accordingly, the authors should revise the text throughout the manuscript to remove or substantially tone down wording such as "predict", "predictive", and "predictable" in both Results and Discussion.

We thank the reviewer for pointing this out. We have revised the manuscript to more accurately reflect the correlative nature of our analysis. Please see pages 8, 10–12, 15–16, 18, 20–22, 44 and 59, lines 220, 278, 305–307, 359–361, 458, 489, 523–525, 613, 645–647, 655, 664, 1279, and 1707.

In addition, strong statements such as:

• "Intriguingly, we found that the RT landscape could predict aphidicolin-induced RT behavior and break propensity" (Abstract)

• "Moreover, we demonstrated that the RT landscapes in untreated cells enable prediction of aphidicolin-induced RT behavior and chromosome break propensity" (End of the Introduction)

should either be removed or substantially revised. Based on Fig. 6C, the predictive performance is only modest even for the CFS-L class (and to a less extent for non-CFS-L_RT-delayed class), while predictive performance appears limited for the other classes.

We have edited the aforementioned statements in the manuscript to tone down the predictive performance. Please see pages 1 and 3, lines 22–23 and 59–61.

Minor comments

1. Page 2: "Recently, using single-cell whole genome sequencing (scWGS) adapted from scRepli-seq technology…(Ref. 13)"
Please remove "adapted from scRepli-seq technology", since the scWGS method used in Ref. 13 was not adapted from scRepli-seq technology.

Done. Please see page 2, line 49.

2. The order of Supplementary Notes should be revised throughout the manuscript. For example, Supplementary Note 3 is cited before Supplementary Note 2, and Supplementary Note 7 appears before Supplementary Note 6.

We have revised the order of Supplementary Notes throughout the manuscript. For Supplementary Note 2 and 3, please see pages 4–5, lines 115, 130 and 141. Since additional Supplementary Notes were added during this revision, the remaining Supplementary Notes were renamed and cited accordingly.

3. Page 7: "They were present closer to long genes (>=300 kb) compared to randomized control with similar RT (Fig. 2B; see Methods)", Please also cite Supplementary Fig. S5A here.

Done. Please see page 8, line 213.

**Reviewer #3 (Remarks to the Author):**

The authors have addressed several points raised in the previous round, notably the concern regarding cell-type specificity of cytogenetic fragile sites. They have provided additional discussion and comparisons across multiple cell lines, which I find acceptable. Compared to the previous version, the study has been substantially improved, and the revised analyses sufficiently support the main conclusions. Considering the technical rigor of the work and the fact that the findings will be of interest to the genome stability community, I believe the manuscript can now be considered for publication.

We thank the reviewer for their positive evaluation of our revised manuscript and for their thoughtful contributions throughout the review process.

**Reviewer #4 (Remarks to the Author):**

I co-reviewed this manuscript with one of the reviewers who provided the listed reports. This is part of the Nature Communications initiative to facilitate training in peer review and to provide appropriate recognition for Early Career Researchers who co-review manuscripts.

We thank the reviewer for their thoughtful contribution throughout the review process.

Thank you for your consideration. Please feel free to contact me with any concerns.

Sincerely yours,

Ichiro Hiratani, Ph.D.
Team Director (Principal Investigator)
Laboratory for Developmental Epigenetics
RIKEN Center for Biosystems Dynamics Research (RIKEN BDR)
2-2-3 Minatojima-minamimachi, Chuo-ku, Kobe, 650-0047 JAPAN
phone: +81-78-306-7179
Email: ichiro.hiratani@riken.jp
https://www.bdr.riken.jp/en/research/labs/hiratani-i/index.html
